# Cuticular wax biosynthesis in blueberries (*Vaccinium corymbosum* L.): Transcript and metabolite changes during ripening and storage affect key fruit quality traits

**DOI:** 10.1093/hr/uhae004

**Published:** 2024-01-09

**Authors:** Yifan Yan, Kristina K Gagalova, Eric M Gerbrandt, Simone D Castellarin

**Affiliations:** Wine Research Centre, Faculty of Land and Food Systems, The University of British Columbia, 2205 East Mall, Vancouver, BC V6T 1Z4, Canada; Canada’s Michael Smith Genome Sciences Centre, 570 W 7th Ave, Vancouver, BC V5Z 4S6, Canada; British Columbia Blueberry Council, 32160 South Fraser Way #275, Abbotsford, BC V2T 1W5, Canada; Wine Research Centre, Faculty of Land and Food Systems, The University of British Columbia, 2205 East Mall, Vancouver, BC V6T 1Z4, Canada

**Keywords:** Triterpenoids, β-diketones, transcriptomics, abscisic acid, fruit water loss, fruit color

## Abstract

In fruits, cuticular waxes affect fruit quality traits such as surface color at harvest and water loss during postharvest storage. This study investigated the transcriptional regulation of cuticular wax deposition in northern highbush blueberries (*Vaccinium corymbosum* L.) in relation to fruit water loss and surface color during ripening and postharvest storage, as well as the effects of abscisic acid (ABA)-mediated changes in cuticular wax deposition on these fruit quality traits. Total cuticular wax content (μg∙cm^−2^) decreased during fruit ripening and increased during postharvest storage. Transcriptome analysis revealed a transcript network for cuticular wax deposition in blueberries. Particularly, five *OSC-Likes* were identified as putative genes for triterpene alcohol production, with *OSC-Like1* and *OSC-Like2* encoding mixed amyrin synthases, *OSC-Like3* encoding a lupeol synthase, and *OSC-Like4* and *OSC-Like5* encoding cycloartenol synthases. The expression of three *CYP716A-like* genes correlated to the accumulation of two triterpene acids oleanolic acid and ursolic acid, the major wax compounds in blueberries. Exogenous ABA application induced the expression of triterpenoid biosynthetic genes and the accumulation of β-amyrin and oleanolic acid, as well as increased the ratio of oleanolic acid to ursolic acid. These changes were associated with reduced fruit water loss. The content of β-diketones was also increased by ABA application, and this increase was associated with increased fruit lightness (measured as L* using CIELAB Color Space by a colorimeter). This study provided key insights on the molecular basis of cuticular wax deposition and its implications on fruit quality traits in blueberries.

## 1. Introduction

Waxes are one of the major components of the cuticle, forming a protective whitish coating on fruit surface during fruit development. Cuticular waxes play critical roles in the restriction of fruit dehydration, prevention of pathogen infection, and modulation of fruit surface color [1–3]. Fruit cuticular waxes are a mixture of very-long-chain (VLC) aliphatic (e.g. fatty acids, alcohols, aldehydes, alkanes, and β-diketones) and cyclic (e.g. triterpenoids and sterols) compounds. The relative abundance of each wax group varies among fruit species and varieties [4], but cuticular wax biosynthesis is conserved among species, with aliphatic and cyclic compounds being synthesized through two major pathways [5, 6].

Biosynthesis of most aliphatic compounds (excluding β-diketones) has been extensively characterized in *Arabidopsis thaliana*, and initiates from C16 and C18 fatty acyl-coenzyme A (CoA) [6]. VLC fatty acids are then produced by fatty acid elongase (FAE) complexes consisting of β-ketoacyl-CoA synthase (KCS), β-ketoacyl-CoA reductase (KCR), β-hydroxyacyl-CoA dehydratase (HCD), and trans-∆^2^-enoyl-CoA reductase (ECR). Fatty acid derivatives are subsequently produced through two branches: i) the acyl reduction branch that produces primary alcohols and esters; and ii) the decarbonylation branch that produces aldehydes, alkanes, secondary alcohols, and ketones [6]. The biosynthesis of β-diketones—mostly studied in wheat *(Triticum aestivum* L.) and barley (*Hordeum vulgare* L.)—is independent from fatty acids and their derivatives, and is controlled by three genes localized in the so-called *CER-cqu* region [7, 8]. However, the β-diketone biosynthetic pathway has not been fully elucidated.

Cyclic compounds are synthesized via the mevalonate pathway, with 2,3-oxidosqualene being the last common precursor [5, 9]. Triterpene alcohols (e.g. α-amyrin, β-amyrin, and lupeol) are direct products of 2,3-oxidosqualene cyclases (OSCs). Most OSCs were identified to produce multiple triterpene alcohols. For instance, in apples (*Malus domestica* L.), MdOSC1 and MdOSC3 produce primarily α-amyrin (80%) and some β-amyrin (20%), while MdOSC5 produces predominantly lupeol (95%) and a small proportion of β-amyrin (5%) [10, 11]. Further, triterpene acids (e.g. ursolic acid, oleanolic acid, and betulinic acid) are synthesized from triterpene alcohols by CYP716A subfamily cytochrome P450 monooxygenases [9, 12].

In blueberries (*Vaccinium* spp*.*), triterpenoids are the most abundant wax compounds in all investigated species and varieties. Interestingly, β-diketones—not commonly detected in the waxes of other fruits—are the second largest group in blueberry waxes, whereas other VLC aliphatic compounds contribute smaller proportions [2, 3, 13]. The biosynthetic pathways underlying the unique and complex profile of blueberry waxes have never been investigated despite the relevance to blueberry fruit quality [14, 15]. Due to the polyploid nature and genome size of blueberries [16], identifying genes responsible for wax deposition can be more challenging in this fruit crop than in model species such as Arabidopsis or tomato (*Solanum lycopersicum* L.). A recent study identified differentially expressed genes (DEGs) in waxy and non-waxy rabbiteye blueberries (*V. ashei* Reade) and suggested that the *FATB* gene—encoding a fatty acyl-ACP thioesterase involved in C16 and C18 fatty acid biosynthesis—determined the waxy phenotype in blueberries [17].

Abscisic acid (ABA)—a key ripening-related hormone that affects fruit pigmentation in non-climacteric fruits including blueberries [18, 19]—was recently found to stimulate cuticular wax deposition in non-climacteric fruits such as navel oranges (*Citrus sinensis* L.) and sweet cherries (*Prunus avium* L.) [20, 21]. For instance, an ABA-deficient mutant of navel orange had impaired biosynthesis and altered morphology of cuticular waxes, which further led to increased cuticle permeability during fruit ripening [20]. Exogenous ABA application to sweet cherries increased cuticular wax content and upregulated expression of key wax-related genes at harvest [21]. Whether ABA modulates wax deposition in blueberries remains unknown. Exogenous ABA application could potentially be used to modify wax deposition and fruit quality traits (e.g. water loss and surface color) affected by waxes in blueberries.

The objectives of this study were: i) to characterize wax deposition in blueberries during fruit ripening and postharvest storage and identify genes involved in wax biosynthesis during this period; ii) to determine how changes in cuticular wax deposition—during fruit ripening and postharvest storage as well as in response to exogenous ABA application—affect fruit water loss and surface color. According to previous studies in blueberries and other fruit species [2, 3, 13, 21], we hypothesized that: i) the expression of wax-related genes change during fruit development and postharvest storage, and these changes affect the cuticular wax content and profile; ii) exogenous ABA application stimulates cuticular wax deposition by inducing both aliphatic and cyclic compounds; and iii) such changes in wax deposition affect fruit water loss and surface color during fruit ripening and postharvest storage. To test these hypotheses, we first characterized the wax content and profile of blueberries during ripening and postharvest storage. Then we conducted *in silico* analyses to identify candidate genes related to blueberry wax production and performed the blueberry transcriptome analysis to characterize the expression profiles of these genes during ripening and postharvest storage and transcript–metabolite relationships. We also applied exogenous ABA to perturbate wax biosynthesis in blueberries and assess the effect of changes in wax content and composition on fruit water loss and surface color.

## 2. Results

### 2.1. Cuticular wax profile in blueberries

Cuticular wax profiling identified a total of 54 wax compounds during fruit ripening and postharvest storage in the northern highbush blueberry cultivars ‘Calypso’ and ‘Last Call’, including eleven fatty acids, seven primary alcohols (referred to as ‘alcohols’ hereafter since no secondary alcohols were detected), five esters, seven aldehydes, twelve alkanes, two β-diketones, seven triterpenoids, and two other cyclic compounds (Tables S1–S2).

In ‘Calypso’, triterpenoids were the most abundant group, comprising an average of 59.6% of total cuticular waxes across all stages; β-diketones contributed 27.5% to total waxes on average; and other aliphatic compounds (i.e. fatty acids, alcohols, esters, aldehydes, and alkanes) were 12.9% on average (Table S1). Oleanolic acid, ursolic acid, and C31 β-diketone were the predominant wax compounds, together accounting for 70.9% of total waxes. The wax profile of ‘Last Call’ was similar to that of ‘Calypso’, except that ursolic acid was more abundant than oleanolic acid in ‘Last Call’ while the opposite was true for ‘Calypso’ (Table S2).

### 2.2. Time-course changes in cuticular wax deposition and berry quality traits

**Figure 1:**
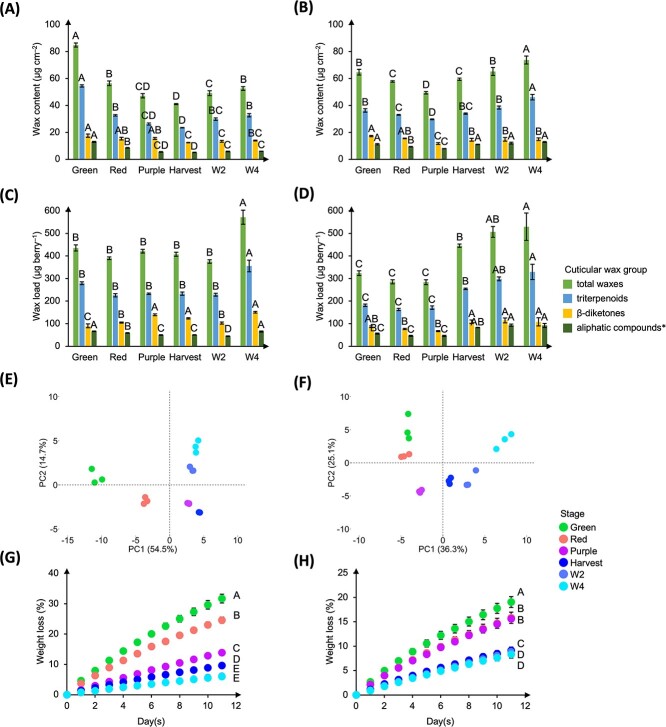
Cuticular wax deposition and its effect on fruit weight loss (%) during fruit ripening and postharvest storage in ‘Calypso’ **(A, C, E, and G)** and ‘Last Call’ **(B, D, F, and H)** blueberries. **(A–B)** Changes in cuticular wax content (μg cm^−2^); **(C–D)** changes in cuticular wax load (μg berry^−1^); **(E–F)** principal component analysis (PCA) on individual wax contents; **(G–H)** fruit weight loss (%) during storage at 20°C and 35% relative humidity (RH) for eleven days. Data presented in **(A–D)** and **(G–H)** are means ± standard error (SE, n = 3), and in **(E–F)** are the means of three replicates. One-way ANOVA was performed in **(A–D)**, different letters identify significant differences of each wax group among stages. Repeated measures one-way ANOVA was performed in **(G–H)**, different letters identify significant differences in fruit weight loss among stages. Means were separated according to LSD tests (*p* < 0.05). The acronyms W2 and W4 stand for two and four weeks after postharvest storage, respectively; ‘^*^’ indicates aliphatic compounds that did not include β-diketones (i.e. fatty acids, alcohols, esters, aldehydes, and alkanes).

Cuticular wax content and profile changed during fruit ripening and postharvest storage (Figure 1A–F, Tables S1–S2). In ‘Calypso’, total wax content (μg∙cm^−2^) decreased (−51.7%) during ripening and increased (+28.4%) during postharvest storage. The highest total wax content was found at Green stage (84.9 μg∙cm^−2^) and the lowest at Harvest stage (41.0 μg∙cm^−2^). However, when expressed on a per-berry-basis, total wax load (μg berry^−1^) remained stable during ripening and increased during storage (Figure 1A and C, Table S1). Similar trends were observed in ‘Last Call’, except that total wax content and total wax load increased from Purple to Harvest stage. The highest total wax content was found at W4 stage (73.7 μg∙cm^−2^) and the lowest at Purple stage (49.4 μg∙cm^−2^) (Figure 1B and D, Table S2).

**Figure 2:**
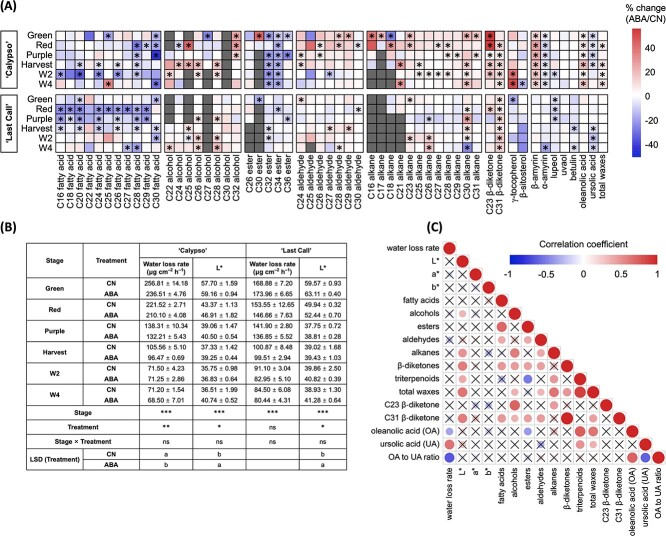
Cuticular wax deposition and berry phenotypic traits in response to exogenous abscisic acid (ABA) application during fruit ripening and postharvest storage in ‘Calypso’ and ‘Last Call’. **(A)** Heatmaps displaying percentage change (%) of total and individual wax contents between ABA and control (CN) treatments in ‘Calypso’ (top) and ‘Last Call’ (bottom); the asterisks identify significant (*p* < 0.05) changes between ABA and CN treatments according to Student’s t-test. **(B)** Table displaying fruit water loss rate (μg cm^−2^ h^−1^) and lightness level (measured as L* by a colorimeter); two-way ANOVA was performed, and no interactions were found between stages and treatments; ‘^***^’ identifies *p* < 0.001, ‘^**^’ identifies *p* < 0.01, ‘^*^’ identifies *p* < 0.05, ‘ns’ identifies a non-significant effect (*p* > 0.05); different letters identify significant differences (*p* < 0.05) between treatments, means were separated according to LSD tests. **(C)** Pearson correlation map (n = 36) displaying the relationships between percentage changes (ABA/CN) in cuticular waxes and percentage changes (ABA/CN) in berry phenotypic traits in ‘Calypso’ and ‘Last Call’ during fruit ripening and postharvest storage; blue circles identify significant (*p* < 0.05) negative correlations, red circles identify significant (*p* < 0.05) positive correlations. Larger and darker circles identify stronger correlations. Crosses identify non-significant (*p* > 0.05) correlations. Data presented in **(A)** and **(C)** are the means of three biological replicates, data presented in **(B)** are the means ± standard error (SE, n = 3).

In both cultivars, the trends of most wax compounds mirrored those of total wax content (Tables S1–S2). In ‘Calypso’, total triterpenoid content decreased (−57.0%) during ripening and increased (+39.5%) during postharvest storage. Total β-diketone content decreased (−29.1%) during ripening and remained stable during postharvest storage (Figure 1A, Table S1). Except for total alcohols that increased, other aliphatic compounds decreased in their contents during ripening. Postharvest changes in these aliphatic compounds varied among chemical groups; total fatty acids and total alcohols increased, total esters and total alkanes remained stable, and total aldehydes decreased (Table S1). In ‘Last Call’, total triterpenoid content decreased (−18.0%) from Green to Purple stage and increased (+55.0%) from Purple to W4 stage. Total β-diketone content decreased (−16.3%) from Green to Purple stage and remained stable from Purple to W4 stage (Figure 1B, Table S2). The contents of other aliphatic compounds mostly decreased from Green to Purple stage and increased from Purple to W4 stage, with two exceptions: i) total alcohol content remained stable from Green to Purple stage; and ii) total ester content decreased from Purple to W4 stage (Table S2).

We highlighted the roles of cuticular waxes in fruit water loss and surface color in our previous studies [2, 3]. Here, we assessed the changes in these phenotypic traits during fruit ripening and postharvest storage. Fruit weight loss (%) was lower for berries approaching Harvest stage. The highest (31.7% in ‘Calypso’ and 19.0% in ‘Last Call’) weight loss was observed at Green stage and the lowest (6.2% in ‘Calypso’ and 8.3% in ‘Last Call’) at W4 stage (Figure 1G–H). Fruit surface color was measured based on CIELAB Color Space. The greenness-redness (a^*^) level increased from Green to Red (‘Calypso’) or Purple (‘Last Call’) stage followed by a decrease until Harvest stage. The lightness (L^*^) and blueness-yellowness (b^*^) levels greatly decreased during ripening. Fruit surface color (L^*^, a^*^, and b^*^) did not change during storage, except for a slight increase in L* from W2 to W4 stage in ‘Calypso’ (Table S3).

### 2.3. Cuticular wax deposition and berry quality traits in response to exogenous ABA application

Exogenous ABA application induced color changes but reduced sugar content during fruit ripening. Percentage of purple and blue fruit was increased by an average of 10.2% and 11.1% in ABA-treated ‘Calypso’ and ‘Last Call’ berries, respectively, across ripening stages. Total soluble solids (TSS) were decreased by an average of 5.2% and 6.3% in ‘Calypso’ and ‘Last Call’, respectively (Figure S1).

Exogenous ABA application affected cuticular wax deposition during ripening and storage in ‘Calypso’ and ‘Last Call’, but only stimulated total cuticular wax content in the former cultivar (Figure 2A, Tables S1–S2). In ‘Calypso’, ABA application increased total triterpenoid content by 6.6% during ripening and storage, being mostly due to increases in β-amyrin (+40.2%) and oleanolic acid (+22.5%). ABA application also increased total β-diketones (+22.4%), total alcohols (25.5%), and total alkanes (13.8%) during ripening and storage. Total aldehydes were unaffected, while total fatty acids (−21.9%) and total esters (−33.2%) were reduced at Green, Red, and Purple stages but unaffected at later stages (Figure 2A, Table S1). In ‘Last Call’, ABA application reduced total triterpenoid content due to decreases in lupeol (−10.4%), betulin (−10.9%), and ursolic acid (−9.8%). Aliphatic compounds generally showed similar responses to ABA application as in ‘Calypso’, except for total alkanes that were unaffected (Figure 2A, Table S2).

**Figure 3:**
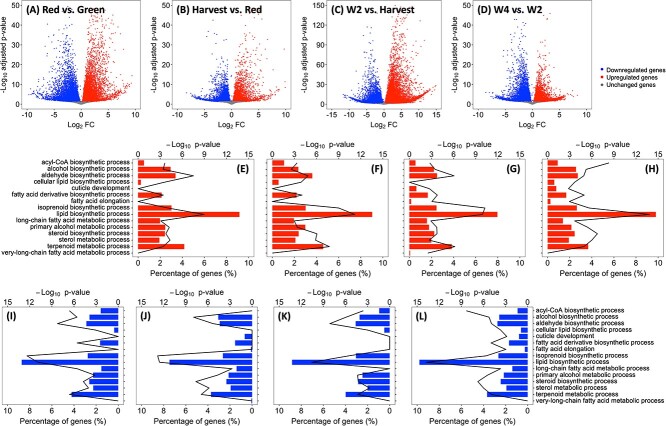
Differential gene expression analysis **(A–D)** and enrichment analysis **(E–L)** in control (CN) ‘Calypso’ blueberries during fruit ripening and postharvest storage. In each row from left to right, each panel represents the comparison between Red and Green stage, Harvest and Red stage, W2 and Harvest stage, and W4 and W2 stage, respectively. **(A–D)** Volcano plots for differentially expressed genes (DEGs, adjusted *p*-value < 0.05 or log_2_ fold change [FC] < −2 or log_2_ FC > 2) between each comparison; blue dots refer to downregulated genes, red dots refer to upregulated genes, and grey dots refer to unaffected genes. **(E–L)** Enriched (false discovery rate [FDR] < 0.01) wax-related gene ontology (GO) terms between each comparison within upregulated (**E–H)** and downregulated **(I–L)** genes; line graph indicates the **–**log_10_*p* value of each GO term, bar graph indicates the percentage of upregulated or downregulated genes in each GO term; **−**log_10_*p*-value = 0 and percentage of upregulated or downregulated genes = 0 identify non-enriched GO terms (FDR > 0.01).

Exogenous ABA application reduced fruit water loss in ‘Calypso’ but not in ‘Last Call’, whereas it increased the lightness level in both cultivars during ripening and storage (Figure 2B, Table S3). Pearson correlation analysis was used to understand the relationships between cuticular waxes and berry phenotypic traits (i.e. water loss and surface color). The analysis was based on percentage changes between ABA and control (CN) treatments to eliminate any changes related to fruit development *per se* and highlight ABA-induced changes (Figure 2C). Increased oleanolic acid content and reduced ursolic acid content were associated with reduced water loss in response to ABA application. As a result, a higher ratio of oleanolic acid to ursolic acid was related to lower water loss (Figure 2C). Cuticular waxes affected fruit surface color mainly by modulating the lightness level. Increased total alcohol, alkane, β-diketones, and triterpenoid contents were associated with increased lightness level in response to ABA application (Figure 2C).

### 2.4. Transcriptomic analysis in ‘Calypso’ blueberries and identification of wax-related genes during fruit ripening and postharvest storage

‘Calypso’ was selected for transcriptome analysis. Purple stage was excluded due to similar wax contents and profiles between Purple and Harvest stages (Figure 1E).

The high throughput sequencing analysis generated 39.3–47.8 millions of raw reads from each blueberry library (Table S4). After removing low-quality reads, a total of 5.8–7.0 Gbp of high-quality clean bases were obtained from each library with Q30 (Phred score) being 94.0% and guanine-cytosine (GC) content being 46.3% on average. The reads were mapped to the genome of *V. corymbosum* cv. ‘Draper’ v1.0 [16]; total mapped reads ranged from 97.3% to 98.0%, and around 60% were uniquely mapped (Table S4). The uniquely mapped reads were used for downstream analyses. A total of 128 559 protein-coding genes were predicted from the blueberry genome [16] and 77 047 transcripts were quantified from ‘Calypso’ blueberries after filtering out those with insufficient levels of expression (i.e. sum of raw counts from all libraries < 10). The hierarchical clustering of transcript expression (Figure S2A) and the Euclidean distances among samples (Figure S2B) revealed a stage-specific transcriptome profile. Particularly, samples collected during ripening were clearly separated from those stored during postharvest.

The differential expression (DE) analysis identified 15 791, 7419, 22 807, and 11 255 differentially expressed transcripts between Green and Red, Red and Harvest, Harvest and W2, and W2 and W4 stages, respectively, in CN berries (Figures 3A-D and S3). A total of 22 307 and 23 878 transcripts were upregulated and downregulated, respectively; 44 and 36 transcripts were consistently upregulated and downregulated, respectively, in all four comparisons (Figure S3).

Enrichment analysis was performed based on DEGs during fruit ripening and postharvest storage. Only wax-related gene ontology (GO) terms are discussed (Figure 3E-L). The GO terms “cuticle development”, “fatty acid elongation”, and “very-long-chain fatty acid metabolic process” were enriched among the downregulated genes from Red to Harvest stage and among the upregulated genes from Harvest to W2 stage (Figure 3E-L). This coincided with decreased contents of fatty acids and other aliphatic compounds during ripening and increased contents during postharvest storage (Table S1).

We searched the literature and databases and created a “cuticular wax reference database” containing 154 protein records related to wax production in different plant species (Table S5). These protein sequences were blasted to the blueberry database and the hits were selected if they met the criteria described in **Supplemental **m**aterial 1**. The selected hits were then checked for Pfam domains, subjected to reciprocal best hit blast (RBHB) analysis, blasted back to the blueberry database, and checked for their phylogeny (**Supplemental **m**aterial 1**). Via these analyses, we identified 205 transcripts related to wax production. Among these, 52 were involved in the synthesis of fatty acid precursors, 34 in fatty acid elongation, 18 in the synthesis of fatty acid derivatives (i.e. alcohols, esters, aldehydes, and alkanes), 15 in β-diketone biosynthesis, and 22 in cyclic compound biosynthesis. Other transcripts included 9 for wax transport and secretion, 40 transcription factors (TFs), and 15 other regulators for wax deposition (Figure S4, Table S6).

### 2.5. Time-course changes in wax-related gene expression and their relationships with cuticular wax contents

Gene co-expression network analysis revealed five major expression patterns (indicated by color modules in Figure S5) for wax-related transcripts during fruit ripening and postharvest storage: i) 49 transcripts (turquoise module) decreased in their expression during ripening and storage, especially from Harvest to W2 stage; ii) 26 (blue module) increased in their expression during ripening and storage; iii) 17 (black module) showed overall low expression during ripening and storage except for a peak at Harvest stage; iv) 16 (brown module) decreased in their expression from Green to Red stage and remained low afterwards; and v) 15 (yellow module) increased in their expression from Green to Red stage and remained stable afterwards (Figure S5, Table S6).

**Figure 4:**
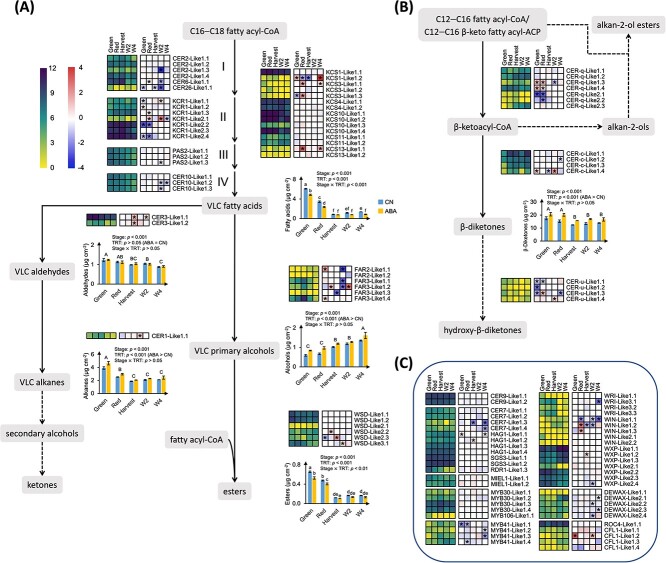
Transcriptional regulation of aliphatic compounds during fruit ripening and postharvest storage and in response to exogenous ABA application in ‘Calypso’. **(A)** Biosynthetic pathway for very-long-chain (VLC) aliphatic compounds (except for β-diketones); **(B)** biosynthetic pathway for β-diketones and derivatives; **(C)** transcription factors (TFs) and other regulatory genes involved in aliphatic compound biosynthesis (presented in the dark blue frame box). Dashed arrows indicate biosynthetic steps producing compounds that are not detected in this study. DESeq2 normalized expression counts in control (CN) treatment are presented at Green, Red, Harvest, W2, and W4 stages (from left to right) in the left heatmap; yellow and purple boxes indicate low and high expression levels, respectively. Log_2_ fold change (FC) (ABA/CN) of expression levels are presented at Green, Red, Harvest, W2, and W4 stages (from left to right) in the right heatmap; blue and red boxes indicate downregulation and upregulation of genes, respectively, by exogenous abscisic acid (ABA) application; asterisks indicate significant differences (adjusted *p*-values < 0.05) according to the differential expression analysis. Data presented in the heatmaps are the means of three biological replicates. Expression of genes involved in each step are presented at the right side of the corresponding arrow, except for the first step (from C16 and C18 fatty acyl-CoA to VLC fatty acids), where I, II, III, and IV stand for the four enzymatic reactions involved in fatty acid elongation, catalyzed by β-ketoacyl-CoA synthase (*CER2*, *CER6*, and *CER26*), β-ketoacyl-CoA reductase (*KCR1*), β-hydroxyacyl-CoA dehydrogenase (*PAS2*), and trans-Δ^2^-enoyl-CoA reductase (*CER10*), respectively. The expression of major genes involved in each reaction are presented at the left side of corresponding arrow, expression of additional *KCS* genes (*KCS1*, *KCS3*, *KCS4*, *KCS10*, *KCS11*, and *KCS13*) are presented at the right side. The contents of individual wax groups at Green, Red, Harvest, W2, and W4 stages in CN (blue bar) and ABA (yellow bar) treatments are presented in bar graphs on the right side of the corresponding wax groups (in grey frames). Data presented in bar graphs are the means ± standard error (SE, n = 3); significant effects of stages, treatments, and their interaction were tested by two-way ANOVA; capital letters identify significantly different (*p* < 0.05) wax contents among stages, when no interaction was found between stages and treatments. One-way ANOVA was further performed to identify significant effects of stages and treatments when a significant interaction was found between stages and treatments; lower-case letters identify significantly different (*p* < 0.05) wax contents among stages and between treatments; means were separated according to LSD tests.

**Figure 5:**
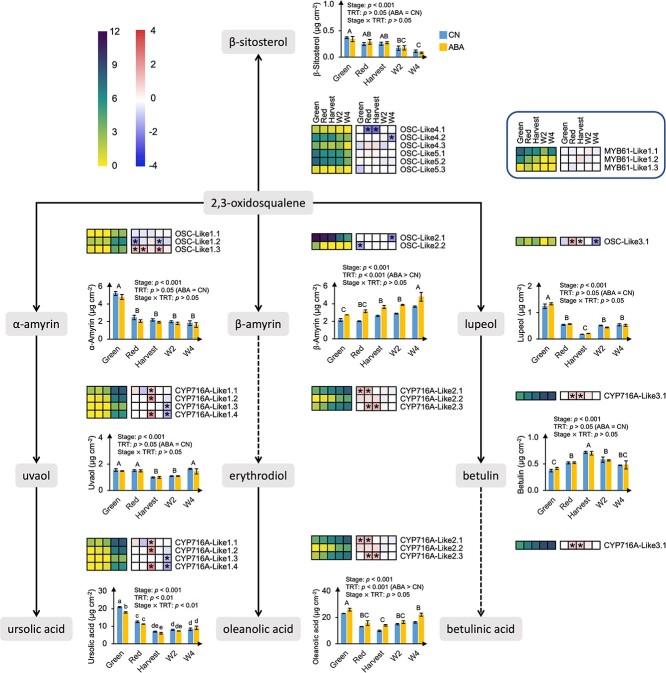
Transcriptional regulation of cyclic compounds during fruit ripening and postharvest storage and in response to exogenous abscisic acid (ABA) application in ‘Calypso’. Dashed arrows indicate biosynthetic steps producing compounds that are not detected in this study. DESeq2 normalized expression counts in control (CN) treatment are presented at Green, Red, Harvest, W2, and W4 stages (from left to right) in the left heatmap; yellow and purple boxes indicate low and high expression levels, respectively. Log_2_ fold change (FC) (ABA/CN) of expression levels are presented at Green, Red, Harvest, W2, and W4 stages (from left to right) in the right heatmap; blue and red boxes indicate downregulation and upregulation of genes, respectively, by exogenous abscisic acid (ABA) application; asterisks indicate significant differences (adjusted *p*-values < 0.05) according to the differential expression analysis. Data presented in the heatmaps are the means of three biological replicates. Expression of genes involved in each step are presented at the right side of the corresponding arrow (*OSC* and *CYP716A* subfamily monooxygenases are potentially multifunctional); expression of transcription factors (TFs, *MYB61*) is highlighted in the dark blue frame box at the top right corner. The contents of individual cyclic compounds at Green, Red, Harvest, W2, and W4 stages in CN (blue bar) and ABA (yellow bar) treatments are presented in bar graphs on the right side of the corresponding cyclic compounds (in grey frames). Data presented in bar graphs are the means ± standard error (SE, n = 3); significant effects of stages, treatments, and their interaction were tested by two-way ANOVA; capital letters identify significantly different (*p* < 0.05) wax contents among stages, when no interaction was found between stages and treatments; one-way ANOVA was further performed to identify significant effects of stages and treatments when a significant interaction was found between stages and treatments; lower-case letters identify significantly different (*p* < 0.05) wax contents among stages and between treatments; means were separated according to LSD tests.

**Figure 6:**
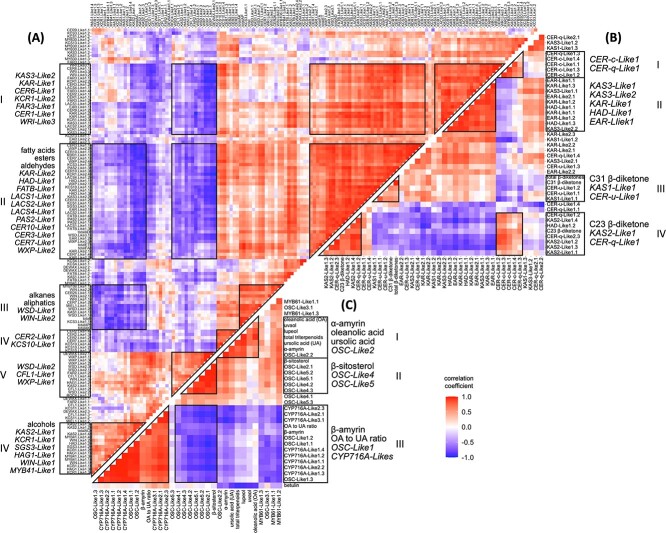
Pearson correlation heatmap (n = 15) between the contents of cuticular waxes and the expression levels of wax-related genes in ‘Calypso’ blueberries. **(A)** Correlation between aliphatic compounds and related genes; **(B)** correlation between β-diketones and related genes; **(C)** correlation between cyclic compounds and related genes. Cuticular wax contents and DESeq2 normalized expression counts were log_10_ (x + 1) transformed; data were re-ordered by hierarchical clustering; the row names and correlation coefficient of panels **(A–C)** could be referred to Tables S7–S9, respectively.

The expression of wax-related genes and the contents of cuticular waxes during fruit ripening and postharvest storage were presented in Figures 4–5. Pearson correlation analyses were performed (Figure 6, Tables S7–S9) to identify the roles of putative wax-related genes in blueberry wax production during ripening and storage.

The expression of fatty acid biosynthetic genes (*KAS1-Like1*, *KAR-Like2*, *EAR-Like1*, and *FATB-Like1*, sequence IDs are presented in Table S6) and fatty acid elongation genes (*KCS1-Like1.1*, *KCS3-Like1*, *PAS2-Like1,* and *CER10-Like1*) decreased during ripening and storage, corresponding with decreased total fatty acid content during this period. Similarly, the expression of an aldehyde- and alkane-forming gene (*CER3-Like1.1*) decreased during ripening and postharvest storage and positively correlated with total aldehyde and alkane contents. In contrast, the expression of an alcohol-forming gene (*FAR2-Like1.2*) increased during ripening and postharvest storage, coinciding with increased total alcohol content (Figures 4A and 6A, Tables S6–S7). The expression of several TFs and aliphatic compound biosynthetic genes also correlated. *WIN-Like2* and *WXP-Like2* showed expression patterns consistent with fatty acid and alkane biosynthetic genes (e.g. *PAS2-Like1*, *CER10-Like1*, and *CER3-Like1.1*), and *WIN1-Like1* and *WXP-Like1* showed expression patterns consistent with an alcohol-forming gene (*FAR2-Like1.2*) (Figure 6A, Tables S6–S7).

The expression of *CER-c-Like1*—putatively involved in β-diketone production—increased during ripening and remained high during postharvest storage; this positively correlated with C23 β-diketone content and negatively correlated with C31 β-diketone content. The expression of *CER-q-Like1* and *CER-q-Like2.3*—putatively involved in β-diketone precursor production—increased during ripening and postharvest storage and positively correlated with C23 β-diketone content. The expression of *CER-u-Like1*—putatively involved in hydroxy-β-diketone production—remained low during ripening and postharvest storage; this may explain the absence of hydroxy-β-diketones in blueberry waxes (Figures 4B and 6B, Tables S6 and S8).

**Figure 7:**
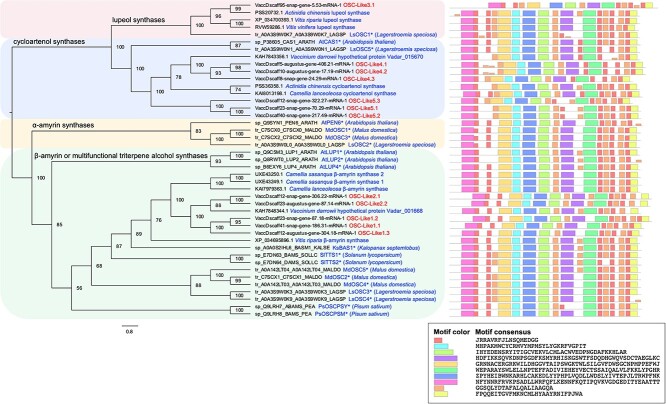
Phylogenetic relationships of putative oxidosqualene cyclases (OSCs) in blueberries (highlighted by red text) and homologs (highlighted by blue text) in other species. The catalytic specificities of OSCs are highlighted by different color frames (pink: lupeol synthases; light blue: cycloartenol synthases; light yellow: α-amyrin synthases; light green: β-amyrin or multifunctional triterpene alcohol synthases). Numbers represent the bootstrap values for each node (1000 replicates). The asterisks indicate functionally characterized genes in other species. Protein motifs of each OSC are displayed in differentially colored boxes. The sequence information of each motif is provided in the legend.

Five OSC genes (*OSC-Like1–5*) were identified as putatively involved in various triterpene alcohol production. In general, protein structure analysis (presented by protein motifs) identified that at least seven protein motifs were shared among blueberry *OSC-Like1–5* and all *OSC* genes from other species (Figure 7). Phylogenetic analysis revealed that the identified *OSC* genes from blueberries and other species were mainly clustered and classified into four groups: lupeol synthases, cycloartenol synthases, α-amyrin synthases, and β-amyrin or multifunctional triterpene alcohol synthases (Figure 7). *OSC-Like1* and *OSC-Like2* clustered in the same clade as *AtLUP1*, *AtLUP4*, *SlTTS1*, and *SlTTS2* which were characterized as β-amyrin (*AtLUP4* and *SlTTS1*) or multifunctional triterpene alcohol (*AtLUP1* and *SlTTS2*) synthases [22–24]. The expression of *OSC-Like1* increased during ripening and storage, positively correlating with β-amyrin content, while the expression of *OSC-Like2* decreased during the same period, positively correlating with α-amyrin content (Figures 5 and 6C, Tables S6 and S9). *OSC-Like3.1* is located in the same clade with *LsOSC1* (Figure 7) which was characterized as a lupeol synthase [25]. The expression of *OSC-Like3.1* remained constant during ripening and decreased from Harvest to W2 stage, showing no relationship with lupeol content (Figures 5 and 6C, Tables S6 and S9). *OSC-Like4* and *OSC-Like5* are located in the same clade with *AtCAS1* and *LsOSC5* (Figure 7) which were characterized as cycloartenol synthases producing the precursor of β-sitosterol, cycloartenol [25, 26]. The expression of *OSC-Like4* and *OSC-Like5* decreased during ripening and postharvest storage, positively correlating with β-sitosterol content. The expression of all *CYP716A-Like* genes—putatively involved in triterpene acid production—increased during ripening and postharvest storage; this negatively correlated with the content of ursolic acid and positively correlated with the ratio of oleanolic acid to ursolic acid (Figures 5 and 6C, Tables S6 and S9). *MYB61-Like1* was identified as a homolog of the TF *BpMYB61* involved in triterpenoid production in birch (*Betula pendula* Roth) [27]; *MYB61-Like1.1* showed an expression pattern consistent with *OSC-Like3.1*, and *MYB61-Like1.2* showed an expression pattern consistent with *OSC-Like2*, *OSC-Like4*, and *OSC-Like5* (Figure 6C, Table S9).

### 2.6. Global changes in blueberry transcriptome and expression patterns of wax-related genes in response to exogenous ABA application

The DE analysis identified 3431, 2456, 4399, 5062, and 4018 differentially expressed transcripts between CN and ABA treatments at Green, Red, Harvest, W2, and W4 stages, respectively (Figures S6 and S7A-E). A total of 9604 and 8319 transcripts were upregulated and downregulated by ABA application, respectively; no transcripts were consistently upregulated or downregulated at all stages (Figure S6).

Enrichment analysis was performed based on DEGs between ABA and CN treatments; only ABA- and wax-related GO terms are discussed in this section (Figure S7F-O). The ABA-related GO term “response to abscisic acid” was the most enriched among upregulated and downregulated genes at each stage (Figure S7F-O). Among downregulated genes, the wax-related GO terms “fatty acid elongation” and “very-long-chain fatty acid metabolic process” were enriched at Green stage and “cuticle development” was enriched at Green, Red, and Harvest stages. These changes coincided with decreased fatty acid content in ABA-treated berries during fruit ripening (Figures 4A and S7F-O). The wax-related GO term “fatty acid derivative biosynthetic process” was enriched among upregulated and downregulated genes, consistent with the different responses of fatty acid derivatives to ABA treatment. For instance, total alcohol content was increased and total ester content was decreased in ABA-treated berries (Figures 4A and S7F-O).

Exogenous ABA application affected the expression of genes related to aliphatic compound production in a stage-specific manner. For instance, *KCR1-Like* genes were downregulated mainly during ripening, and *PAS2-Like1* and *CER10-Like1* were downregulated during postharvest storage (Figure 4A, Table S6). ABA application affected the expression of TFs for aliphatic compound production mainly during postharvest storage. *WIN-Like1*, *WXP-Like2.4*, and *DEWAX-Like2* were downregulated at W2 stage, and *MYB41-Like1*, *WRI-Like3.1*, *WIN-Like1.1*, and *DEWAX-Like2* were downregulated at W4 stage (Figure 4C, Table S6). Exogenous ABA application had no effects on the expression of genes related to β-diketone production, with three exceptions: i) *CER-c-Like1.2* was downregulated at W4 stage; ii) *CER-c-Like1.4* was upregulated at Green and W2 stages and downregulated at Harvest stage; and iii) *CER-q-Like1* was upregulated and *CER-q-Like2* was downregulated at Green and Red stages (Figure 4B, Table S6). Exogenous ABA application affected cyclic compound accumulation mainly by regulating *CYP716A-Like* gene expression. *CYP716A-Like1*, *CYP716A-Like2*, and *CYP716A-Like3.1* were upregulated at one or two stages during ripening, and *CYP716A-Like1* was downregulated at W4 stage. ABA application also affected *OSC-Like* genes in a stage-specific manner. For instance, *OSC-Like1.2* was downregulated at Green and W2 stages, and *OSC-Like1.3* was upregulated at Green, Red, and W2 stages (Figure 5, Table S6).

### 2.7. Transcript–metabolite networks related to cyclic compound biosynthesis

**Figure 8:**
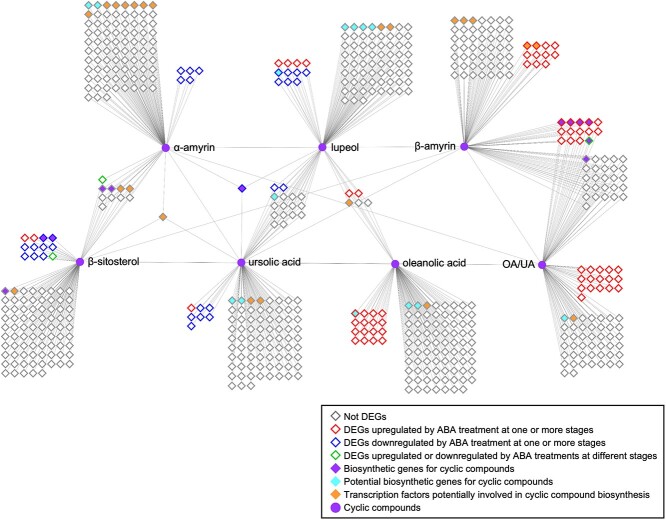
Predicted transcript–metabolite networks related to cyclic compounds in blueberries during fruit ripening and postharvest storage, and in response to exogenous ABA application. Transcripts and metabolites are represented by diamond and circle nodes, respectively. Edges represent associations (r > 0.8 and *p* < 0.05) between transcripts and metabolites. The top 100 correlators for each metabolite are shown. Node borders in grey represent transcripts that are not deferentially expressed by ABA treatment at any stage; node borders in red represent transcripts that are upregulated by ABA treatments at one or more stages; node borders in blue represent transcripts that are downregulated by ABA treatments at one or more stages; node borders in green represent transcripts that are upregulated or downregulated by ABA treatments at different stages. Node colors in purple represent transcripts involved in cyclic compound biosynthesis (*OSC-Like* and *CYP716A-Like* genes identified in section 2.4); node colors in cyan represent transcripts potentially involved in cyclic compound biosynthesis (protein sequences of these potential transcripts share the same Pfam with OSC and CYP716A proteins); node colors in orange represent transcription factors (TFs, protein sequences of these potential transcripts contain at least one Pfam for transcriptional regulation) potentially involved in cyclic compound biosynthesis.

A transcript–metabolite network analysis was performed to better understand the relationships between transcriptome changes and cyclic compound production, and identify putative genes controlling the accumulation of these compounds in blueberries (Figure 8). The analysis focused on major cyclic compounds (α-amyrin, β-amyrin, lupeol, oleanolic acid, ursolic acid, β-sitosterol, and the ratio of oleanolic acid to ursolic acid) and their top-100-correlated transcripts (based on Pearson correlation coefficient) (Figure 8).

Eleven out of 20 transcripts identified as triterpenoid biosynthetic genes (in section 2.4) were amongst the top-100-correlated transcripts, further confirming their putative roles in determining the production of cyclic compounds. Particularly, *OSC-Like2.1* was a common top correlator for α-amyrin, lupeol, and ursolic acid, and *MYB61-Like1.2* was a common top correlator for α-amyrin, ursolic acid, and β-sitosterol. Among the top-100-correlated transcripts, 40 transcripts, including *CYP716A-Like* genes, were shared by β-amyrin and the ratio of oleanolic acid to ursolic acid (Tables S10).

The integrated network analysis also identified other 14 transcripts (green diamonds) potentially involved in triterpenoid biosynthesis, as the protein sequence of these top correlating transcripts shared the same Pfam domains with characterized OSC and CYP716A proteins (Tables S6 and S10). These genes include two transcripts correlating to α-amyrin biosynthesis, five transcripts correlating to lupeol biosynthesis, two transcripts correlating to ursolic acid biosynthesis, three transcripts correlating to oleanolic acid biosynthesis, one transcript correlating to both ursolic acid and lupeol biosynthesis, and one transcript correlating to the ratio of oleanolic acid to ursolic acid). The integrated network analysis also identified 23 TFs (orange diamonds) that are potentially involved in cyclic compound regulation, according to their Pfam domains (Figure 8, Table S10).

## 3. Discussion

### 3.1. Time-course changes in cuticular wax deposition

The extensive profiling of cuticular waxes during fruit ripening and postharvest storage in this study suggests that decreased total wax content (μg cm^−2^) observed during ripening is mainly attributed to fruit expansion, since the total wax load (μg berry^−1^) remained constant across ripening stages. In contrast, increased total wax content and total wax load during storage suggested *de novo* biosynthesis of cuticular waxes during this period. This is the first study determining the comprehensive time-course changes in cuticular wax content and profile in northern highbush blueberries. Chu et al. [13] investigated the deposition of cuticular waxes during blueberry ripening and postharvest storage in a southern highbush cultivar (*V. corymbosum* L. × *Vaccinium darrowii* Camp. cv. ‘Legacy’) and a rabbiteye cultivar (*V. ashei* Reade cv. ‘Brightwell’) and reported an opposite trend to the one found in our study. In addition, Yang et al. [28] found that cuticular waxes thickened during fruit ripening in northern highbush blueberries cv. ‘Elliott’.

Various trends in cuticular wax deposition have been documented in different species and varieties during ripening and storage, depending on the genetic background and storage conditions. For instance, the total wax content in grapes (*Vitis vinifera* L.) and sweet cherries peaked at or slightly before the onset of ripening and decreased during ripening as fruit expanded [29, 30], consistent with our study. In tomatoes, increased total wax content during early fruit development was followed by either a plateau (cv. ‘MicroTom’) or a continuous increase (cv. ‘Ailsa Craig’) during ripening [31, 32]. During postharvest storage, total wax content increased in apples (cv. ‘Starkrimson’) and pears (cv. ‘Korla’) stored at 0–1°C [33, 34], which were similar conditions as those in our study. In contrast, total wax content decreased in apples, pears, and blueberries when stored at temperatures of 3–4°C or higher [13, 35].

Despite these various trends in wax deposition among fruit species and varieties, active wax biosynthesis commonly initiates from early fruit development [13, 29–32]. For instance, in Chu et al. [13] and our study, total wax content averaged 104.2 and 74.8 μg cm^−2^, respectively, at the green-fruit stage when the first sampling occurred. This high content at early stages might be related to the roles of cuticular waxes in the restriction of fruit water loss and inhibition of pathogen infection [30, 36]. In general, younger fruits are more susceptible to abiotic and biotic stresses due to their specific surface structure (e.g. presence of stomata and lenticels that might favor water loss) and fruit composition (e.g. low sugar levels that might be preferred by pathogens such as *Uncinula necator* and *Colletotrichum acutatum* causing powdery mildew in grapes and anthracnose fruit rot in blueberries, respectively) (also discussed in section 3.3) [37–39], and early deposition of cuticular waxes may protect the fruits from water loss and pathogen infection during these stages [40, 41].

### 3.2. Transcriptional regulation of cuticular wax biosynthesis and deposition

This study identified for the first time the genes involved in cuticular wax biosynthesis in northern highbush blueberries and their regulation on cuticular wax deposition during fruit ripening and postharvest storage as well as in response to exogenous ABA application.

The decreased total wax content during ripening and the increased total content during storage was mainly driven by changes in triterpenoids—the most abundant wax group in blueberry waxes. The major enzymes involved in triterpenoid biosynthesis include OSCs that catalyze the rate-limiting step and produce triterpene alcohols, and CYP716A subfamily monooxygenases that catalyze a three-step oxidation and produce triterpene acids [12]. According to phylogenetic analysis (Figure 7), *OSC-Like1* and *OSC-Like2* are putative β-amyrin or multifunctional triterpene alcohol synthases involved in the production of β-amyrin and/or other triterpene alcohols [10, 11, 25]. Based on our correlation analysis and network analysis (Figures 6 and 8), it is likely that β-amyrin and α-amyrin are the major products from *OSC-Like1* and *OSC-Like2*, respectively, in blueberries. The expression of *OSC-Like3* and the content of lupeol did not correlate in our analysis, although *OSC-Like3* is located in the same clade as *LsOSC1* that encodes a lupeol synthase in *Lagerstroemia speciosa* L. [25]. Protein motif identification revealed slight difference between *OSC-Like3* and *LsOSC1* (Figure 7). Considering the potential multifunctionality of OCS enzymes, it is likely that other *OSC-Like* genes (e.g. *OSC-Like1* and *OSC-Like2*) might coordinate with *OSC-Like3* for lupeol production in blueberries. Shibuya et al. [42] proposed that during the evolution of higher plants, two branches of lupeol synthases were generated: one is known as authentic lupeol synthase branch that includes *LsOSC1*, and the other as multifunctional triterpene alcohol synthase branch that includes *AtLUP1* (Figure 7). Previous studies also revealed that some multifunctional triterpene alcohol synthase (e.g. AtLUP1, MdOSC4, and MdOSC5) can function to produce both β-amyrin and lupeol [10, 43]. In this regard, *OSC-Like1* and *OSC-Like2*, as putative β-amyrin or multifunctional triterpene alcohol synthases, might also be involved in lupeol production in blueberries. Phylogenetic analysis showed that *OSC-Like4* and *OSC-Like5* are located in the same clade as *AtCAS1* and *LsOSC5*, which are responsible for cycloartenol biosynthesis in Arabidopsis and *L. speciosa* L., respectively [25, 26]. Although cycloartenol was not detected in blueberry waxes, the positive correlation between the expression of *OSC-Like4* and *OSC-Like5* and the content of β-sitosterol suggested a potential role of these two genes in sterol production. We observed a strong positive correlation between the expression of *CYP716A-Likes* and the ratio of oleanolic acid to ursolic acid. *CYP716A* subfamily monooxygenases have been functionally characterized in several species such as grapes and sweet basil (*Ocimum basilicum* L.), but the substrate preferences have not been reported for any of these *CYP716A* subfamily genes [9, 12]. Here, we speculate that in blueberries *CYP716A-Like* genes lead to ursolic acid or oleanolic acid production depending on the availability of their precursors (i.e. α-amyrin and β-amyrin). This study identified candidate genes for cyclic compound biosynthesis in blueberries based on genomic analysis and highlighted their roles based on phylogenetic analysis and association networks. The integrated network analysis between transcripts and metabolites (Figure 8) also suggested transcripts that share the same Pfams with putative cyclic compound biosynthetic genes identified based on genomic analysis. These transcripts might also play a critical role in cyclic compound production. However, functional characterization (e.g. by transforming blueberry candidate genes into yeast or plant models and testing wax profiles) is necessary to validate the specific roles of these candidate genes in cyclic compound biosynthesis.

β-diketones are the second most abundant group of blueberry waxes, with their contents decreasing during ripening and remaining stable during storage. Although *HvCER-c* and *HvCER-q* were demonstrated to control β-diketone biosynthesis in barley, the expression of *CER-c-Like* and *CER-q-Like* genes identified in this study did not correlate with the content of total β-diketones or C31 β-diketone—the predominant β-diketone. This suggests a limited role of *CER-c-Like* and *CER-q-Like* genes in blueberry β-diketone production. Hen-Avivi et al. [7] and Alberto (2020) [44] recently proposed that β-diketone biosynthesis initiates from C12 to C16 β-keto fatty acyl-acyl carrier protein (ACP) rather than C12 to C16 β-keto fatty acyl-CoA that was proposed by Von Wettstein-Knowles (1979) [8]. The C12 to C16 β-keto fatty acyl-ACP are intermediates during fatty acid biosynthesis and are produced by β-ketoacyl-ACP synthase I (KASI)—the first and rate-limiting enzyme in fatty acid synthase (FAS) complexes [6, 45]. We identified *KAS1-Like* genes in blueberries and found a positive correlation between *KAS1-Like1.1* expression and C31 β-diketone content, highlighting its potential role in β-diketone biosynthesis. In addition, we also found potential *CER-q-Like3* genes that were filtered out during wax gene identification but showed a strong correlation with C31 β-diketone content (Figure S4P). Further functional characterization is required to verify the role of potential *CER-q-Like3* genes in β-diketone biosynthesis.

Other aliphatic compounds (i.e. fatty acids, alcohols, esters, aldehydes, and alkanes) contributed to small proportions of the total waxes in blueberries, and most of them decreased in their contents during fruit ripening. This might be related to the insufficient fatty acid precursors associated with the decreased expression of fatty acid biosynthetic and elongation genes (e.g. *FAS-Like1*, *FATB-Like1*, *PAS2-Like1*, and *CER10-Like1*) during ripening and postharvest storage. In contrast to fatty acids and alkanes, total alcohols increased in their content during ripening; this was consistent with Dimopoulos et al. [29] where decreased contents of most aliphatic compounds (e.g. fatty acids and alkanes) and increased content of total alcohols were observed during ripening in grapes. According to our transcript–metabolite correlation analysis, increased total alcohol content during ripening might be related to *FAR3-Like1*, which is homologous to the major alcohol-forming gene *AtCER4* in Arabidopsis [46]. *WXP-Like1* and *WXP-Like2* are homologous to *MtWXP1*, which regulates cuticular wax deposition in *Medicago truncatula*, mainly by activating alcohol-forming genes [47]. Our analysis also suggested that *WXP-Like1* and *WXP-Like2* regulate aliphatic compound production in blueberries, the former regulating alcohol-forming genes and the latter regulating fatty acid biosynthesis and elongation genes.

In this study, the ripening-related hormone ABA was applied to two blueberry cultivars at the beginning of fruit ripening to modulate wax production and subsequently determine the impacts on fruit water loss and surface color in blueberries (discussed below in sections 3.3 and 3.4). Exogenous ABA application affected wax profile in both cultivars but stimulated wax production only in ‘Calypso’. Differences in wax production between the two cultivars were mainly related to differential changes in triterpenoids. Particularly, β-amyrin and oleanolic acid were induced by ABA application in ‘Calypso’ but reduced or unaffected in ‘Last Call’. In ‘Calypso’, ABA application activated triterpenoid biosynthesis by stimulating the transcription of *OSC-Like* and *CYP716A-Like* genes. In particular, *OSC-Like1.3*—putatively involved in β-amyrin production—was upregulated by ABA application during ripening and storage, suggesting its potential role in promoting β-amyrin accumulation and providing precursors for oleanolic acid production in blueberries. Similarly, in birch stems and leaves, ABA application upregulated the expression of triterpenoid biosynthetic genes (e.g. *BpW* for lupeol synthesis, *BpY* for β-amyrin synthesis, and *BpCYP97B62* and *BpCYP89S1* for betulinic acid and oleanolic acid synthesis) and stimulated the production of triterpenoids (e.g. lupeol, betulinic acid, and oleanolic acid) [48].

### 3.3. Relationships between cuticular waxes and fruit water loss

We observed a dramatic decrease in water loss as blueberry ripened and during postharvest storage. Fruit water loss is influenced by various factors, and the contribution of each factor might change during fruit development. Sugar accumulation during fruit ripening leads to significantly decreased osmotic potential, which is commonly associated with reduced water loss in fruits [49]. In addition, immature blueberries might have stomata on their surfaces [50]. These stomata account for a third possible pathway for water loss in blueberries, in addition to the cuticle and stem scar [3, 14]. Therefore, decreased water loss during fruit ripening might be more related to increases in sugar levels and dysfunctionality of stomata rather than changes in cuticular waxes [50, 51]. On the contrary, our results indicate that cuticular waxes may play a more critical role in restricting the water loss during postharvest storage when sugar levels remain relatively stable and no active stomata are present.

Exogenous ABA application reduced fruit water loss in ‘Calypso’. In many other species, ABA was found to play a critical role in drought resistance by activating wax biosynthesis and reducing cuticle permeability [52, 53]. Our correlation analysis focused on ABA-induced changes in cuticular waxes and fruit water loss to diminish the effect of potential non-causal relationships determined by developmental changes. Based on this analysis, we found that ABA application reduced fruit water loss mainly by increasing the ratio of oleanolic acid to ursolic acid. While we cannot prove causality, the relationships observed suggest a potential role of the ratio of oleanolic acid to ursolic acid in blueberry water loss.

Triterpenoids are commonly reported to increase water permeability of cuticles in various plant species, whereas aliphatic compounds decrease water permeability [36, 54]. For instance, correlation analysis revealed that a higher triterpenoid proportion was associated with a higher water loss rate among 14 pepper genotypes, whereas a higher aliphatic compound (especially alkane) proportion was associated with a lower water loss rate [36]. In Arabidopsis leaves, the ectopic expression of *AtLUP4* (involved in β-amyrin biosynthesis) led to the accumulation of β-amyrin and increased water permeability [54]. Vogg et al. [55] proposed that the water barrier is largely formed by the intracuticular wax layer that is mainly determined by aliphatic compounds and further modified by triterpenoids located in this layer. Our study is the first rendering the potential role of the ratio of oleanolic acid to ursolic acid in affecting fruit water loss. This is consistent with what previously found in both blueberries and grapes [3, 56]. It is worth mentioning that triterpenoids dominate both blueberry and grape waxes, whereas contribute to small or negligible amounts in Arabidopsis and pepper waxes. We postulated that aliphatic compounds—commonly observed to negatively affect water loss in plant species [36, 55]—might have a limited role in restricting the water loss in blueberries and grapes, considering the small amounts of these compounds in the waxes of both species. Consistent with our results, Moggia et al. [14] found that a higher content of ursolic acid correlate with higher blueberry water loss. The role of individual triterpenoids in affecting water loss in fruits require further investigation. It is plausible that some triterpenoids (e.g. oleanolic acid) might be more effective in reducing water loss than others (e.g. ursolic acid) [57], and these compounds may function as a secondary barrier following aliphatic compounds in restricting water loss, especially in species whose waxes are predominated by triterpenoids rather than aliphatic compounds.

### 3.4. Relationships between cuticular waxes and fruit surface color

Time-course changes in fruit surface color during blueberry ripening are primarily determined by anthocyanin accumulation [19], which was not the main focus of this study and hence not investigated. Although the percentage of purple and blue fruit was increased by ABA application in this study, the redness (a*) and blueness (b*) levels were unaffected. Instead, exogenous ABA application affected fruit surface color by increasing lightness (L*), which was associated with the increased total alkane and total β-diketone contents according to our correlation analysis. The role of β-diketones in determining fruit lightness (also described as glaucousness) was demonstrated in wheat leaves by ectopic study [7] and was highlighted in blueberries [2]. In cucumbers, where the synthesis of β-diketones is absent, the glaucous phenotype showed higher expression of alkane-forming genes (e.g. *CsCER1*, *CsWAX2*, and *CsCER7*) and produced more alkanes compared to the non-glaucous phenotype [58, 59]. These observations suggest that both alkanes and β-diketones could play key roles in determining the glaucous phenotype in different plant and fruit species. In blueberries, aliphatic compounds comprise predominantly β-diketones that are 5–10 times higher in concentration compared to alkanes; therefore, the role of alkanes in affecting glaucousness might have been masked by β-diketones. A recent transcriptomic study found that the expression of *FATB* was closely related to the waxy coating in rabbiteye blueberries [17]. We identified putative *FATB-Like1* in this study, and the expression of *FATB-Like1* coincided with the trend in fruit lightness during ripening and storage. However, ABA application had no effects on *FATB-Like1* expression, indicating a limited role of *FATB-Like1* in determining fruit lightness in blueberries in response to ABA application.

The functional characterization of the genes involved in triterpenoid and β-diketone biosynthesis identified in this study will be of assistance to breeding programs aiming to improve blueberry quality traits such as shelf-life and surface color. For example, new breeding selections with an increased ratio of oleanolic acid to ursolic acid and a higher β-diketone content will potentially have fruits with lower water loss during storage and lighter surfaces at harvest and during postharvest storage, respectively. These improved quality traits will lead to better shelf-life and marketability of blueberries.

## 4. Material and methods

### 4.1. Plant material and experimental design

This study was conducted in a commercial blueberry planting (near 49°N, 122°W) in Abbotsford, BC Canada in 2021, using ‘Calypso’ and ‘Last Call’ (*V. corymbosum* L.). The experiments were conducted independently for the two cultivars due to their phenological differences. Bushes were planted in north-to-south oriented rows in 2016 and managed according to standard industry recommendations (https://www2.gov.bc.ca/gov/content/industry/agriservice-bc/production-guides/berries/blueberries). The experimental row was divided into three blocks with a complete randomized block design; within each block, three bushes comprised a single replicate for each treatment (CN and ABA) with two bushes as a buffer between treatments. Exogenous ABA (1000 ppm, in 1% ethanol with 0.1% Tween 20) was applied one week before ripening and at the beginning of ripening (i.e. color break) (June 30 and July 8 for ‘Calypso’; July 26 and August 3 for ‘Last Call’), while 1% ethanol with 0.1% Tween 20 was applied to CN bushes. The treatments were applied by spraying until run-off after sunset to avoid ABA degradation [18].

Berry samples were collected at Green, Red, Purple, and Harvest stages at one-week intervals (Figure S8). The percentage of purple and blue fruit was recorded on each sampling date. Berries were harvested at the timing of first commercial pick (i.e. 50–60% purple and blue fruit). At harvest, additional berries were collected and stored for two (W2) or four (W4) weeks at 0.5°C and 95% relative humidity (RH) (Figure S8).

At each stage, a total of 55 berries were collected for each replicate and used for the following analyses: i) 15 berries for water loss assessment (section 4.2); ii) ten berries for fruit surface color measurement (section 4.2); iii) 15 berries for wax extraction and analysis (section 4.3) and for total soluble solids (TSS) analysis using a digital refractometer (Reichert A2R200, Reichert GmbH, Seefeld, Germany); and iv) 15 berries for transcriptomic analysis (sections 4.4–4.6). Berry samples for transcriptomic analysis were flash-frozen in the field using dry ice. All other samples were transferred within 3 hours of sampling in portable coolers at 4°C to a refrigerator at 0–1°C in the laboratory at the University of British Columbia (Vancouver, BC, Canada).

### 4.2. Berry quality assessment

Fruit water loss was assessed using individual berries stored in sealed desiccators at 20°C and 35% RH according to Yan and Castellarin (2022) [3]. Berries were stored for eleven days, and individual berry weight was recorded every 24 h. Fruit water loss was calculated as percent of weight loss (%) and amount of weight loss per unit surface area per hour (μg cm^−2^ h^−1^). Berry surface area was calculated according to Yan and Castellarin (2022) [3].

Fruit surface color was measured at three random positions on the surface of each berry (excluding the stem scar and calyx area) using a benchtop colorimeter (LabScan XE Reflected Color Spectrophotometer, HunterLab, Reston, VA, USA) according to Yan et al. [2].

### 4.3. Cuticular wax extraction and analysis

Cuticular waxes were extracted and derivatized according to Yan and Castellarin (2022) [3]. Cuticular wax profile was determined and wax content was quantified by gas chromatography–mass spectrometry (GC–MS) analysis using the same instruments and conditions as in Yan and Castellarin (2022) [3]. Cuticular wax content was expressed as wax amount per unit surface area (μg cm^−2^) and wax amount per berry (μg berry^−1^).

### 4.4. RNA extraction

According to the preliminary results from metabolite analysis (section 3.1), ‘Calypso’ berries from all but the Purple stage were selected for molecular analysis. Blueberry RNA was extracted using a Spectrum™ Plant Total RNA Kit (Sigma Aldrich, Oakville, ON, Canada), according to manufacturers’ instructions with some modifications. Different amounts of blueberry powder were used for samples collected at different stages (i.e. 100 mg for Green samples, 200 mg for Red samples, and 500 mg for Harvest, W2, and W4 samples). Another modification was using 700 μL instead of 500 μL of lysis buffer in the first lysing step. The quality and quantity of RNA were assessed by gel electrophoresis and NanoDrop-1000 (Thermo Fisher Scientific, Waltham, MA, USA).

### 4.5. RNA-sequencing and read mapping

The extracted RNA sample was purified to obtain mRNA, broken into small fragments, and synthesized for cDNA for library construction. The quality of cDNA library was evaluated using a bioanalyzer (Agilent Technologies Inc., Santa Clara, CA, USA). RNA sequencing was carried out at Novogene (Sacramento, CA, USA) using the Illumina NovaSeq 6000 platform according to manufacturer’s instructions; paired-end reads of 150 bp with approximately 20 million reads per sample were generated.

The reads were inspected for their sequencing quality through Fastqc v0.11.9 and combined in MultiQC v1.14 for comparison. The reads were filtered with FastP v0.20.1 and read pairs were removed if the percentage of bases lower than Q15 was higher than 40% in at least one read (**Supplemental material 2A**). The resulting reads were screened for contamination with BioBloomTools v2.3.3, using a series of Bloom filters containing k-mers from viruses, bacteria, fungi, mites and aphids (**Supplemental material 2B**).

We aligned the reads against the genome of *V. corymbosum* cv. ‘Draper’ v1.0 [16] using STAR v2.7.10a with customized parameters (**Supplemental material 2C**). The genome was indexed using the annotated genes on the reference genome. The reads were aligned with a two-pass mode where the genomic splice junction coordinates from the first pass were included in the second pass for a refined mapping. Only uniquely mapped reads were used for downstream analyses. The reads per gene were quantified using STAR (—quantMode GeneCounts) (Table S11) and subsequently used in DE analysis.

We then performed DE analysis and GO enrichment analysis to understand transcriptome expression during fruit ripening and postharvest storage as well as in response to ABA application. DE analysis was performed and visualized using R packages DESeq2 v1.36.0 and ggVennDiagram v1.2.2, respectively; a gene was considered to be differentially expressed if the adjusted *p*-value (padj) < 0.05 or log_2_ fold change (FC) < −2 or > 2. GO enrichment analysis was performed based on Pfam domains of DEGs using dcGO (http://supfam.org/SUPERFAMILY/cgi-bin/dcenrichment.cgi) with a false discovery rate (FDR) threshold of 0.01. Gene co-expression network analysis was further performed using R package WGCNA v1.71.

### 4.6. Cuticular wax gene identification

Candidate genes for cuticular wax biosynthesis and regulation were identified according to the steps described in **Supplemental material 1**. Protein domains were identified by InterProScan v5.53–87.0. RBHB analysis was performed by MMseqs2 v12.113e3. Phylogenetic analysis was performed to check the phylogeny of putative transcripts; protein sequences were aligned with Clustal Omega (https://www.ebi.ac.uk/Tools/msa/clustalo/) using default parameters, protein distances were calculated and trees constructed by IQTREE (http://iqtree.cibiv.univie.ac.at) using a neighbor-joining method, and trees were visualized by FigTree v1.4.4.

### 4.7. Statistical analysis

All data presented are the mean of three replicates. All statistical analyses were performed using IBM SPSS Statistics v24.0 (Chicago, IL, USA) and R v3.6.2. Student’s t-test was performed to detect treatment effects on wax deposition at each stage. One-way analysis of variance (ANOVA) was used to detect developmental effect on wax deposition and berry phenotypic traits in CN berries. Two-way ANOVA was performed to detect developmental and treatment effects as well as their interaction on wax deposition and berry phenotypic traits. Repeated measures one-way ANOVA was performed to detect developmental effect on fruit water loss in CN berries during eleven days of storage. Means were separated according to least significant difference (LSD) tests. Pearson correlations were calculated to determine the relationships between wax content and berry phenotypic traits and ascertain the relationships between wax-related gene expression and wax content. Integrated transcript–metabolite network analysis was performed between major cyclic compounds and their top-100-correlated transcripts using Cytoscape (version 3.10.1). The top-100-correlated transcripts were identified based on Pearson correlation coefficient. The types of statistical analysis, data transformation, and replication are specified in the corresponding figure legends and table captions. All packages and software used for data processing and analysis in this study are presented in Table S12.

## Acknowledgements

This project was supported by the Canadian Agricultural Partnership, a federal-provincial-territorial initiative, under the Canada-BC Agri-Innovation Program. The program is delivered by the Investment Agriculture Foundation of BC (Grant Number INV098). Authors would like to acknowledge the British Columbia Blueberry Council (BCBC) and MITACS Program (Grant number IT14908) for their financial support. Authors would also like to thank Chengxi (Stephen) Li and Chelsea Harris for their assistance in sample collection and preparation.

## Supplementary Data

Supplementary data is available at *Horticulture Research* online.

## Conflict of interest statement

No potential conflict of interest was reported by the authors.

## Data Availability Statement

Data is openly available in a public repository that issues datasets with DOIs. All raw sequences reads have been deposited in NCBI Sequence Read Archive (https://www.ncbi.nlm.nih.gov/sra) under BioProject PRJNA995759. The BioSample accessions are from SAMN36503517 to SAMN36503546.

## Supplementary Material

Web_Material_uhae004
