# Supplementary material for: Cuticular wax biosynthesis in blueberries (Vaccinium corymbosum L.): Transcript and metabolite changes during ripening and storage affect key fruit quality traits
Source: Hortic Res. 2024 Jan 9;11(3):uhae004. doi: 10.1093/hr/uhae004 (PMC10923646; doi:10.1093/hr/uhae004)
Supplement: Web_Material_uhae004 [file web_material_uhae004.zip › Supplemental figures.docx]

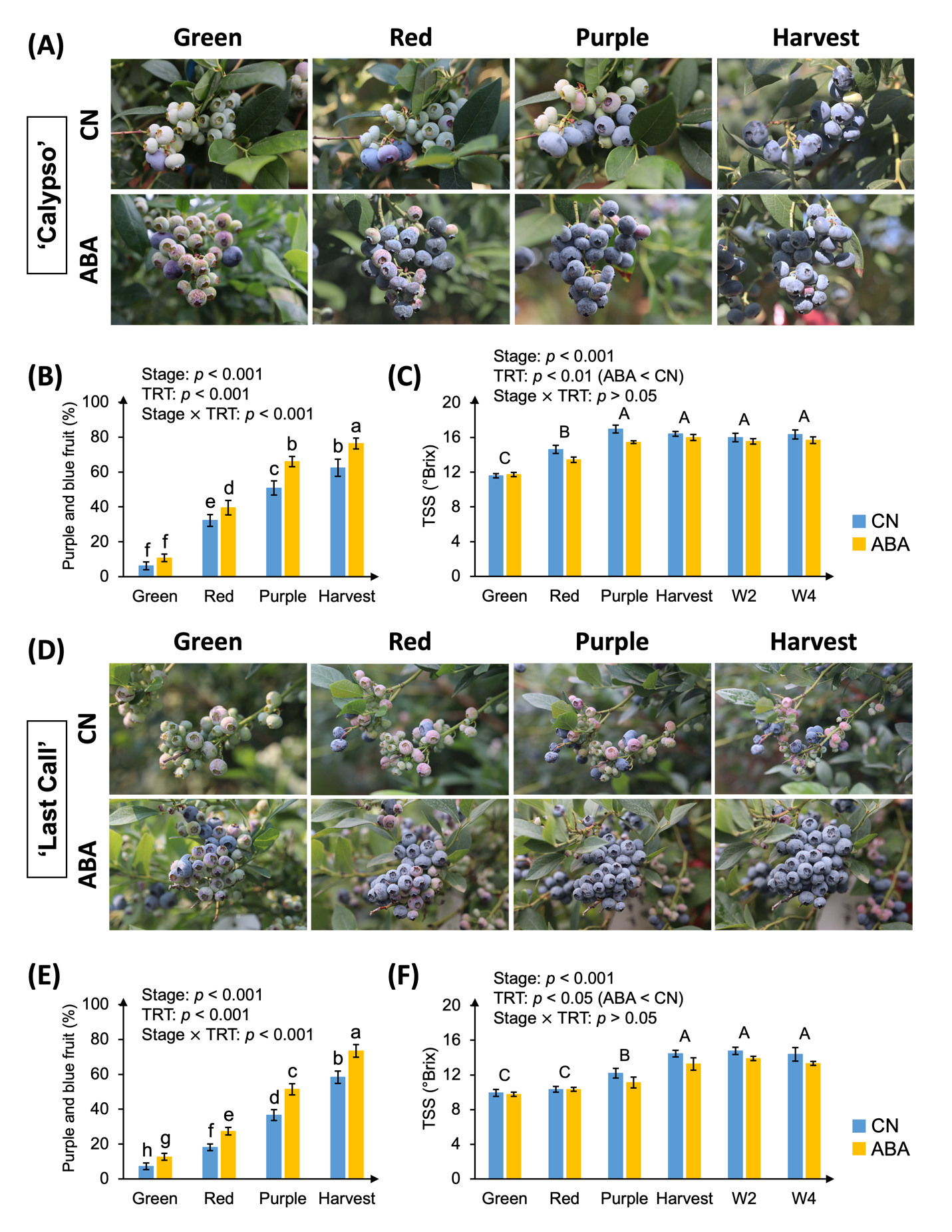


**Figure S1.** Blueberry quality during fruit ripening and postharvest storage in ‘Calypso’ **(A–C)** and ‘Last Call’ **(B–D)**. **(A)** Field blueberries during fruit ripening in ‘Calypso’; **(B)** percentage (%) of purple and blue fruit during fruit in ‘Calypso’; **(C)** total soluble solids (TSS) during fruit ripening and postharvest storage in ‘Calypso’; **(D)** field blueberries during fruit ripening in ‘Last Call; **(E)** percentage (%) of purple and blue fruit during ripening in ‘Last Call; **(F)** TSS during fruit ripening and postharvest storage in ‘Last Call. Data presented in **(B–C)** and **(E–F)** are the means ± standard error (SE, n = 3). Two-way ANOVA was performed in **(B–C)** and **(E–F)**; one-way ANOVA was further performed in **(B)** and **(E)** as a significant interaction was found between stages and treatments, different lower-case letters identify significant differences among stages and between treatments; no interaction was found between stages and treatments in **(C)** and **(F)**, different upper-case letters identify significant differences among stages. Means were separated according to LSD tests. The acronyms W2 and W4 in **(C)** and **(F)** stand for two and four weeks after postharvest storage, respectively.


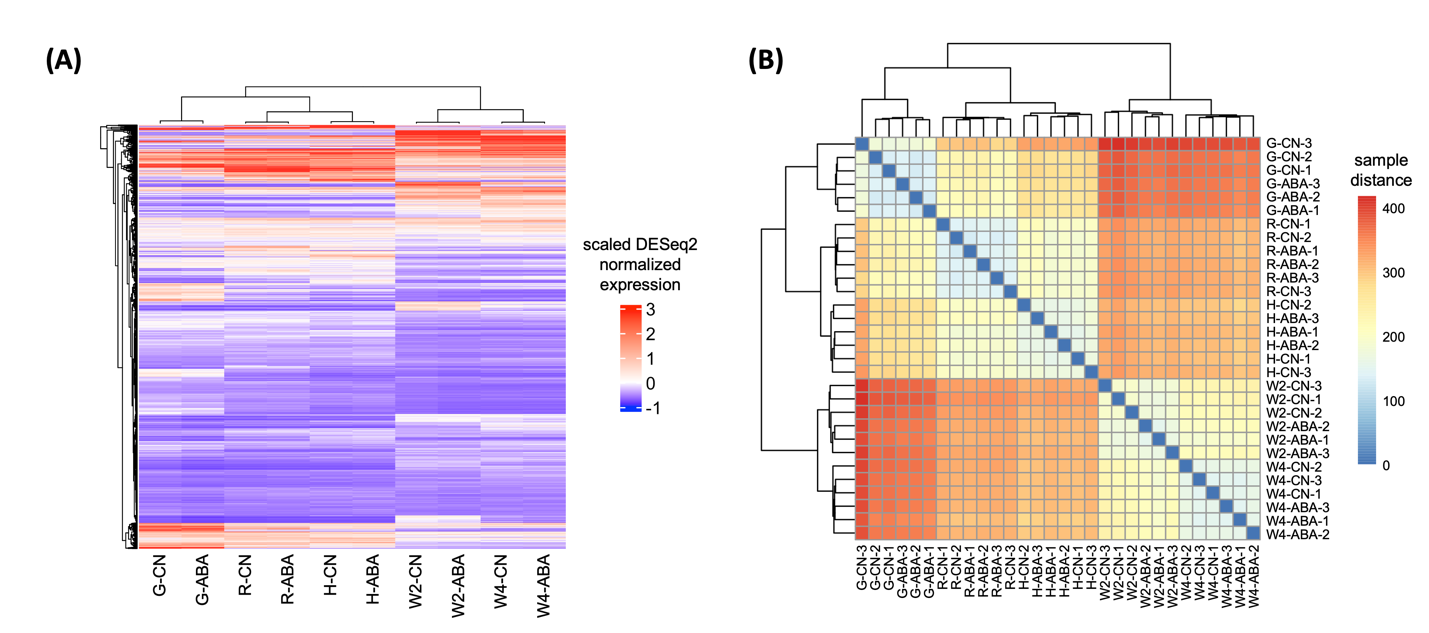


**Figure S2.** Heatmap showing overall expression of the transcriptome **(A)** and sample distances based on the transcriptome **(B)**. **(A)** Expression pattern of 77047 transcripts in ‘Calypso’ blueberries; data presented were normalized by DESeq2 and scaled to mean = 0 and standard deviation = 1. **(B)** Euclidean distances among samples based on transcriptomic profile after regularized log (rlog) transformation. The acronyms G, R, H, W2, and W4 stand for Green, Red, Harvest, W2 (two weeks after postharvest storage), and W4 (four weeks after postharvest storage) stages, respectively.


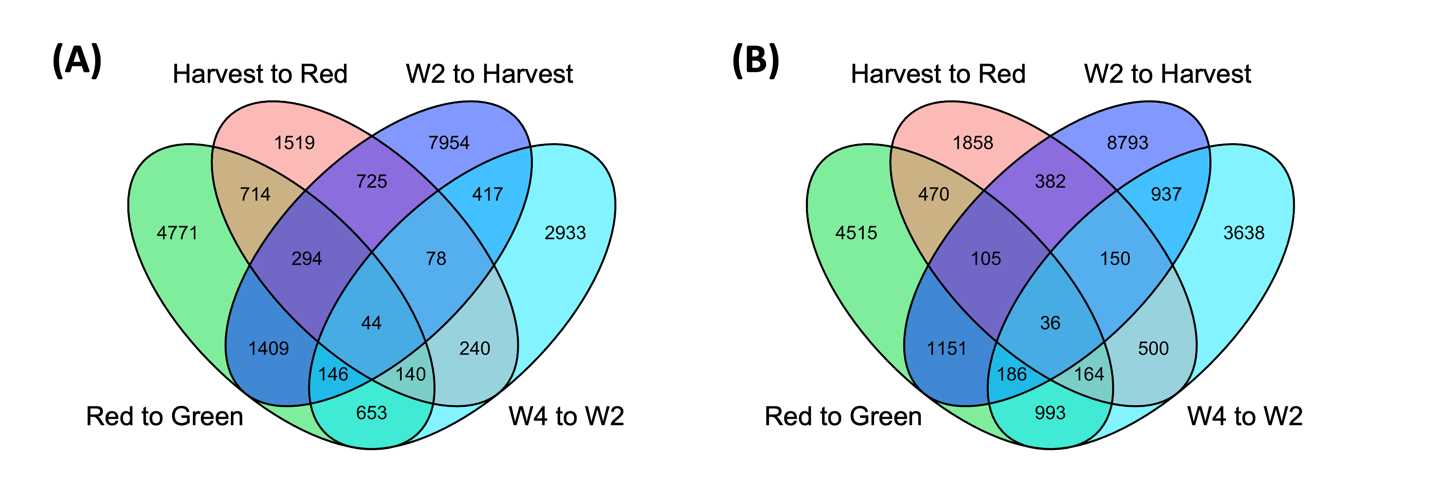


**Figure S3.** Venn diagrams displaying the number of differentially expressed genes (DEGs) during fruit ripening and postharvest storage in ‘Calypso’ blueberries according to DEseq2 analysis. **(A)** Upregulated genes; **(B)** downregulated genes.


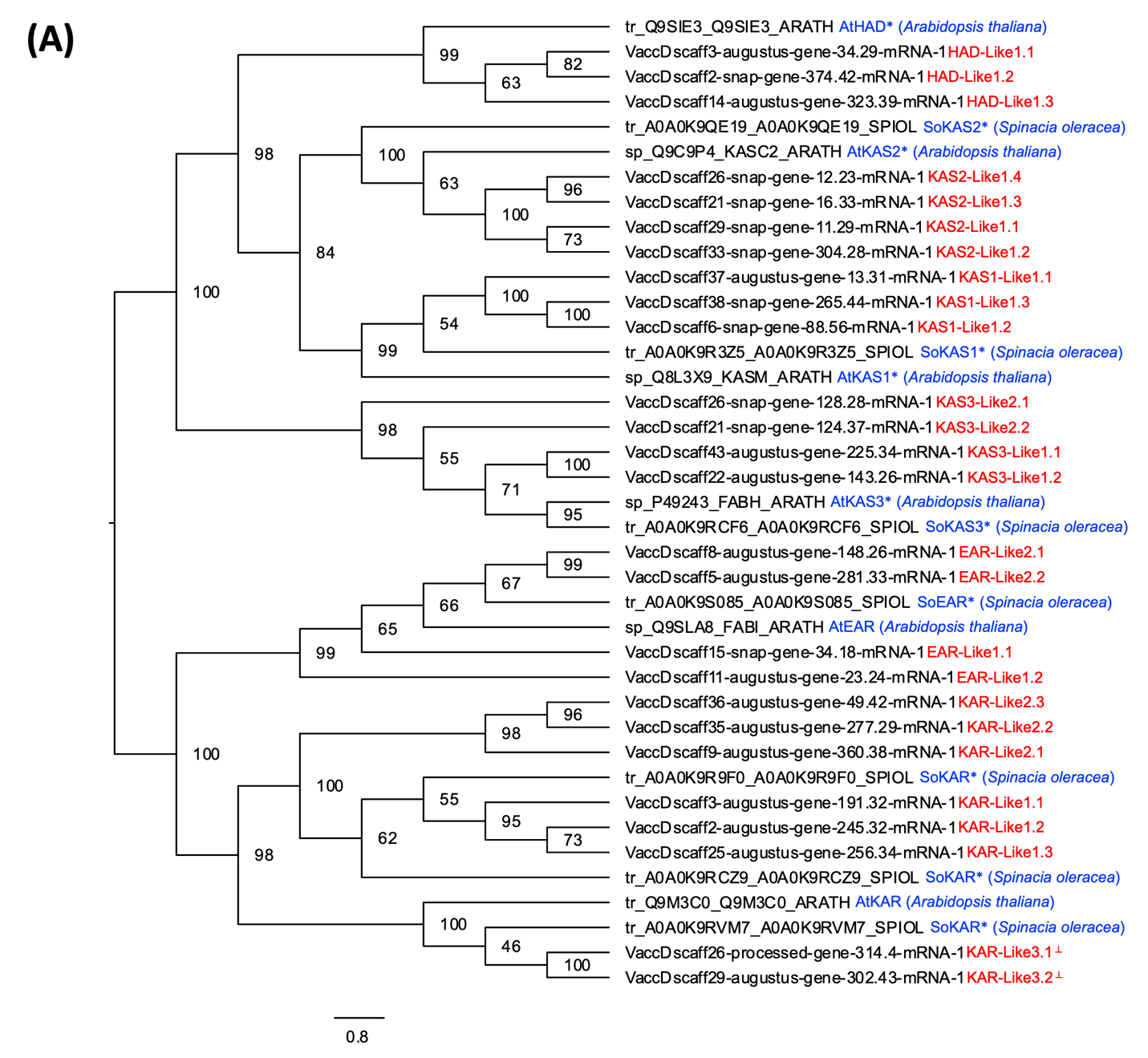

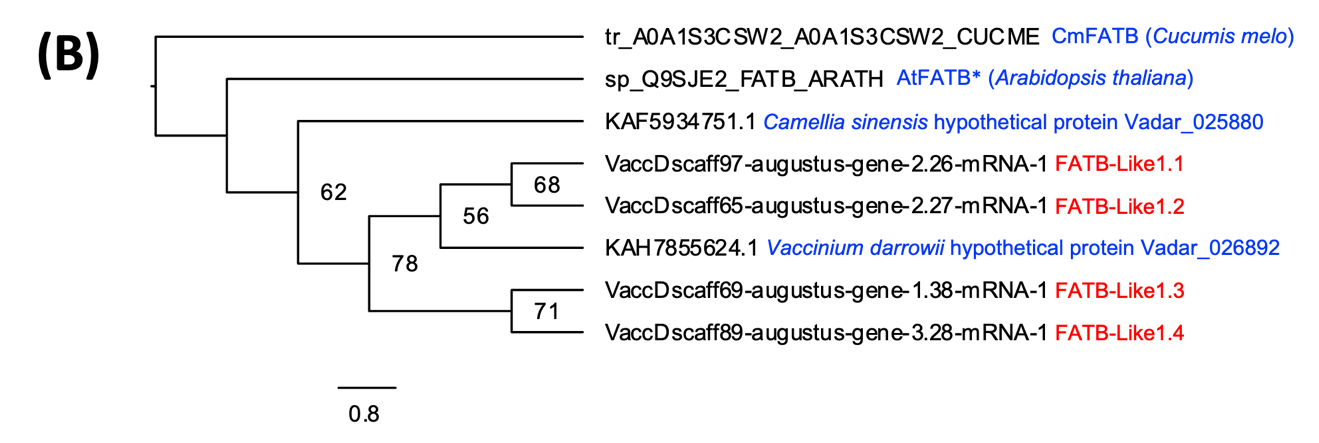

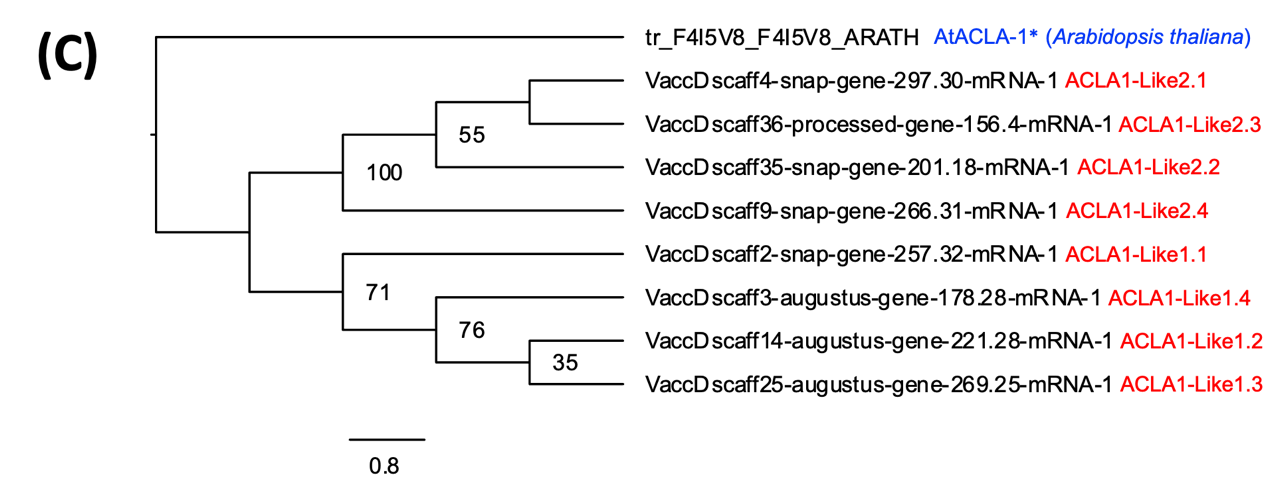

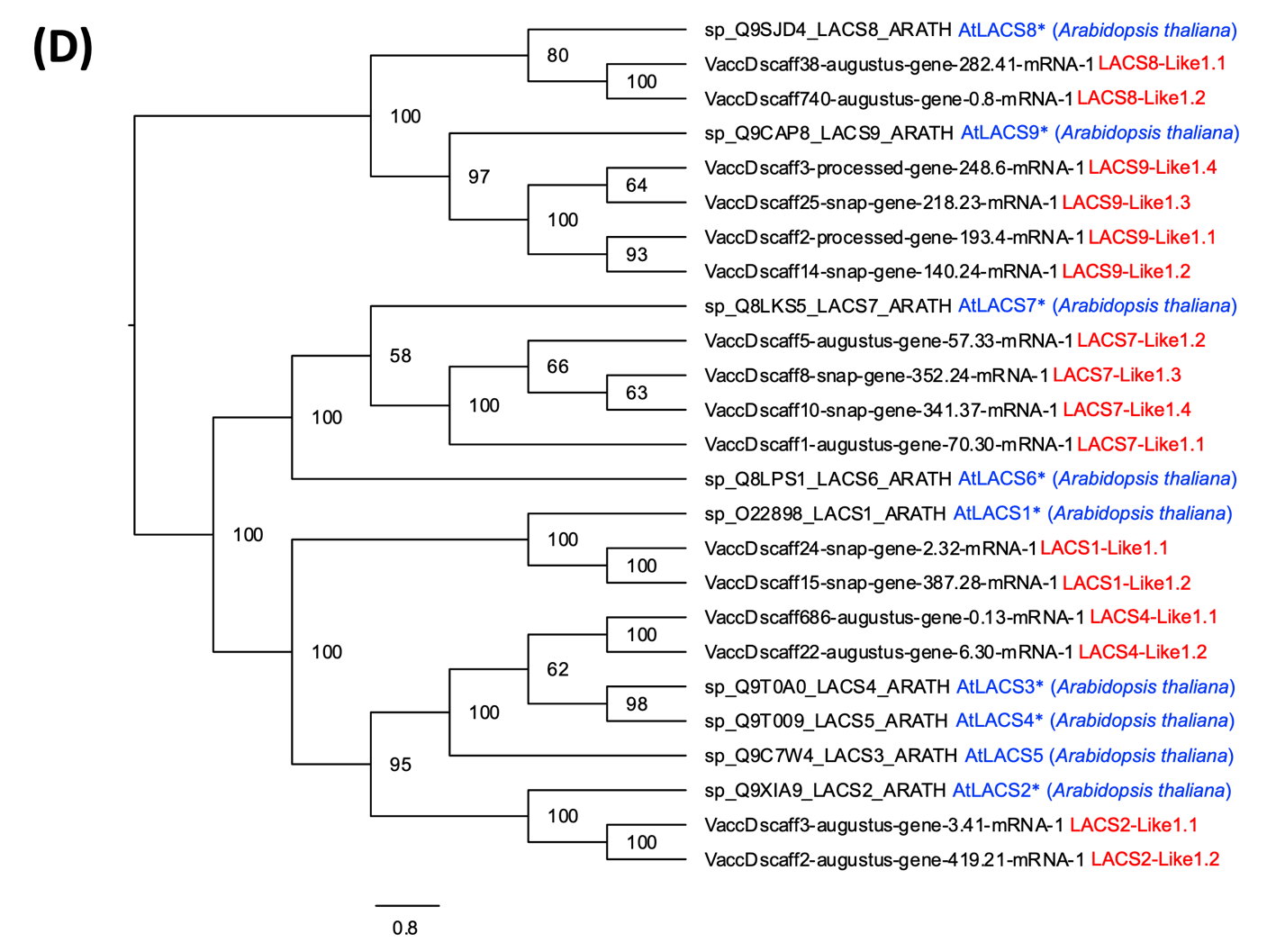

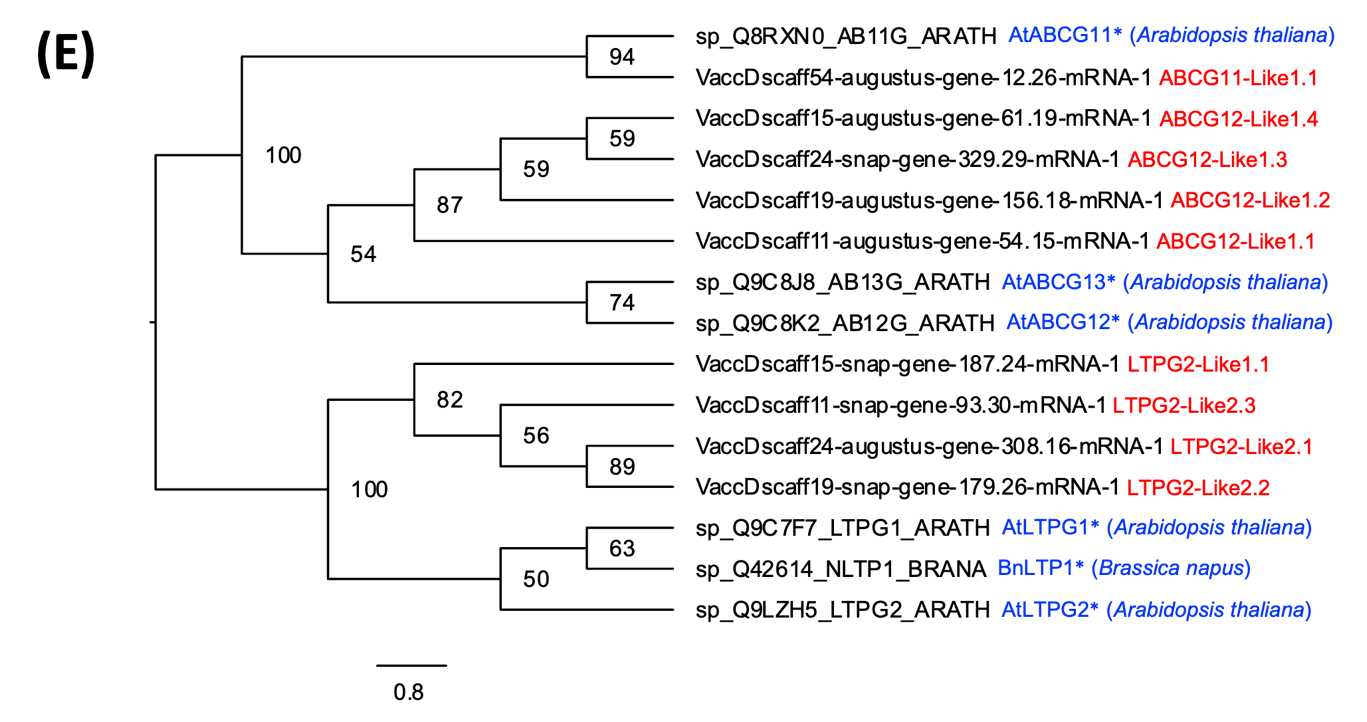

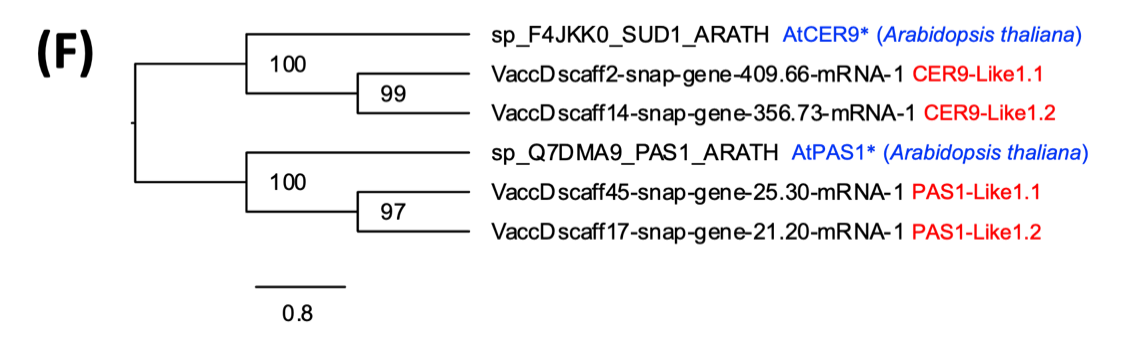

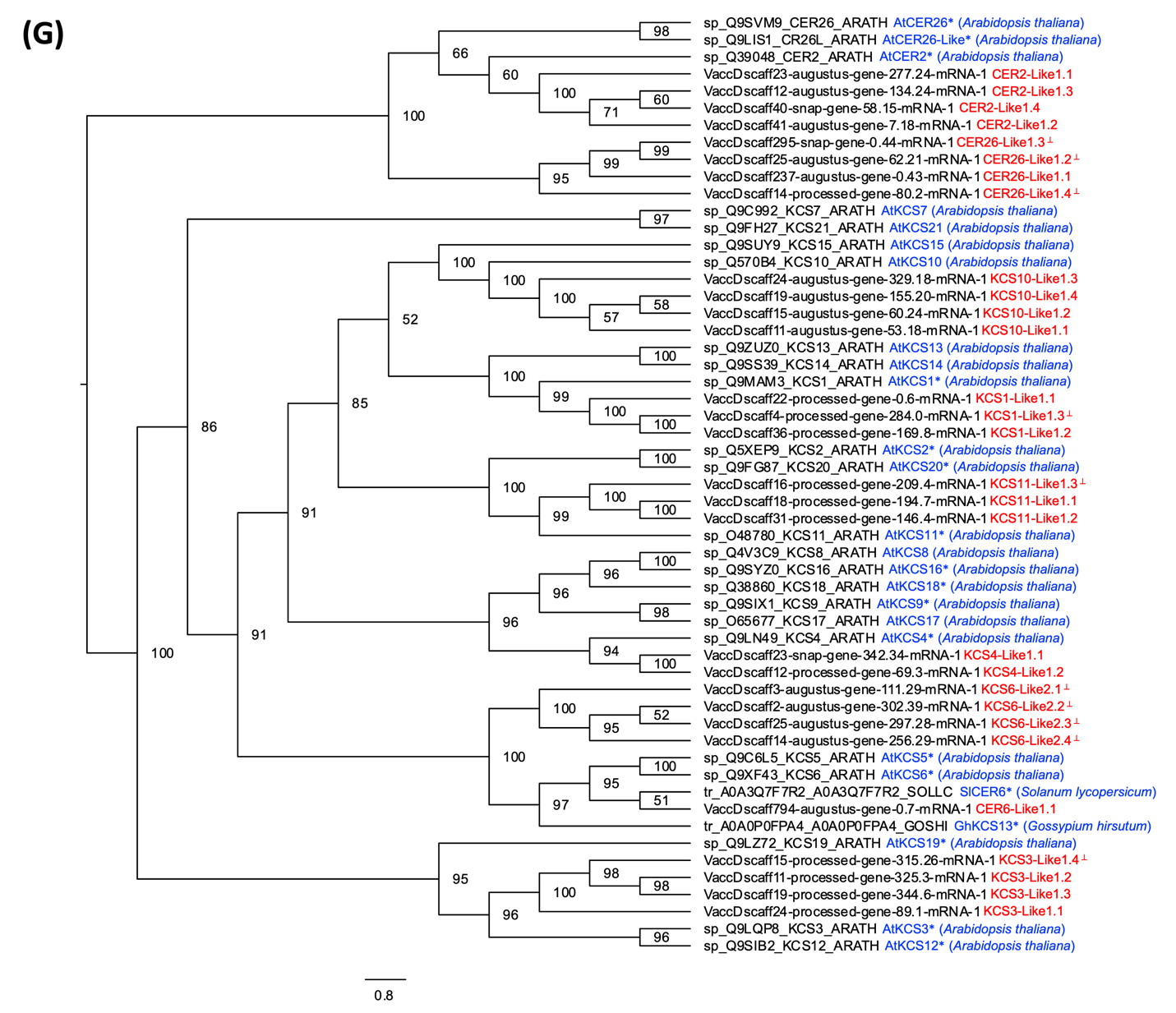

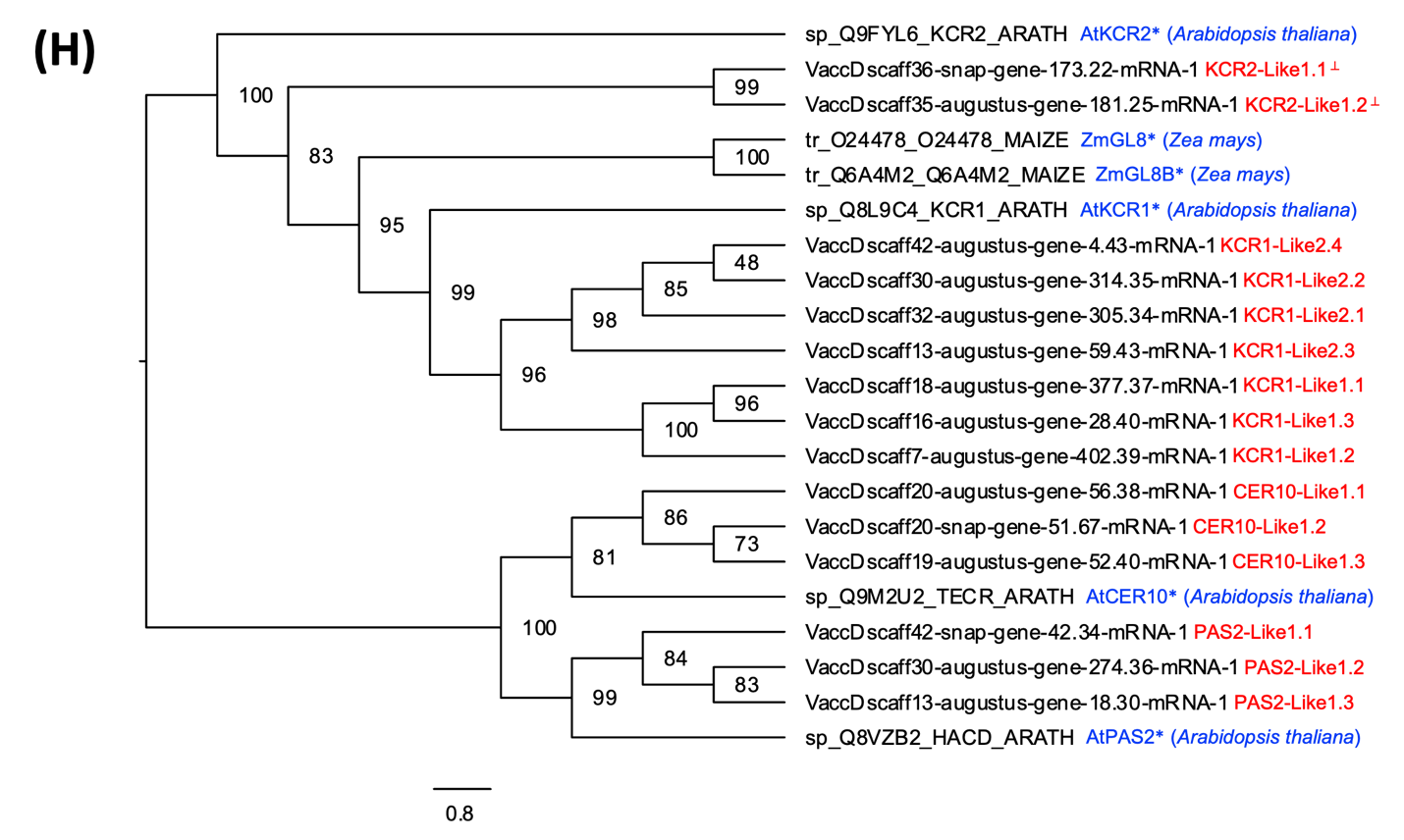

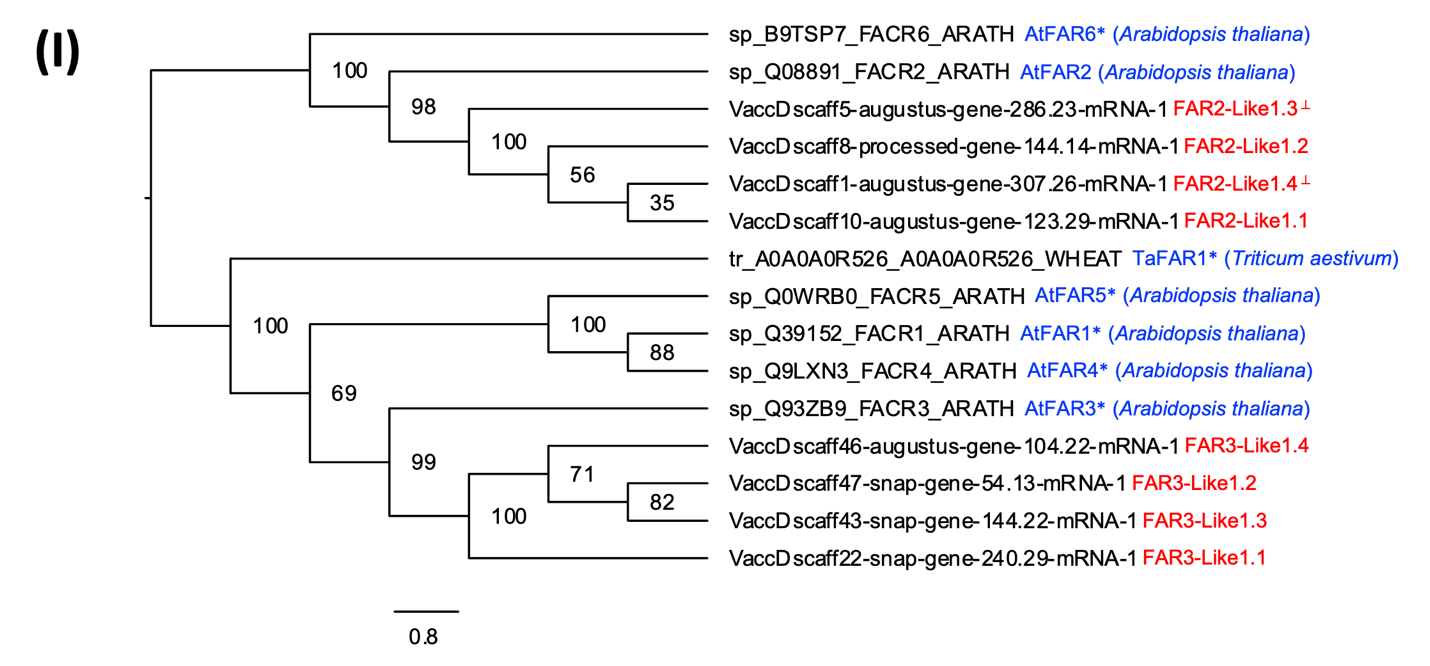

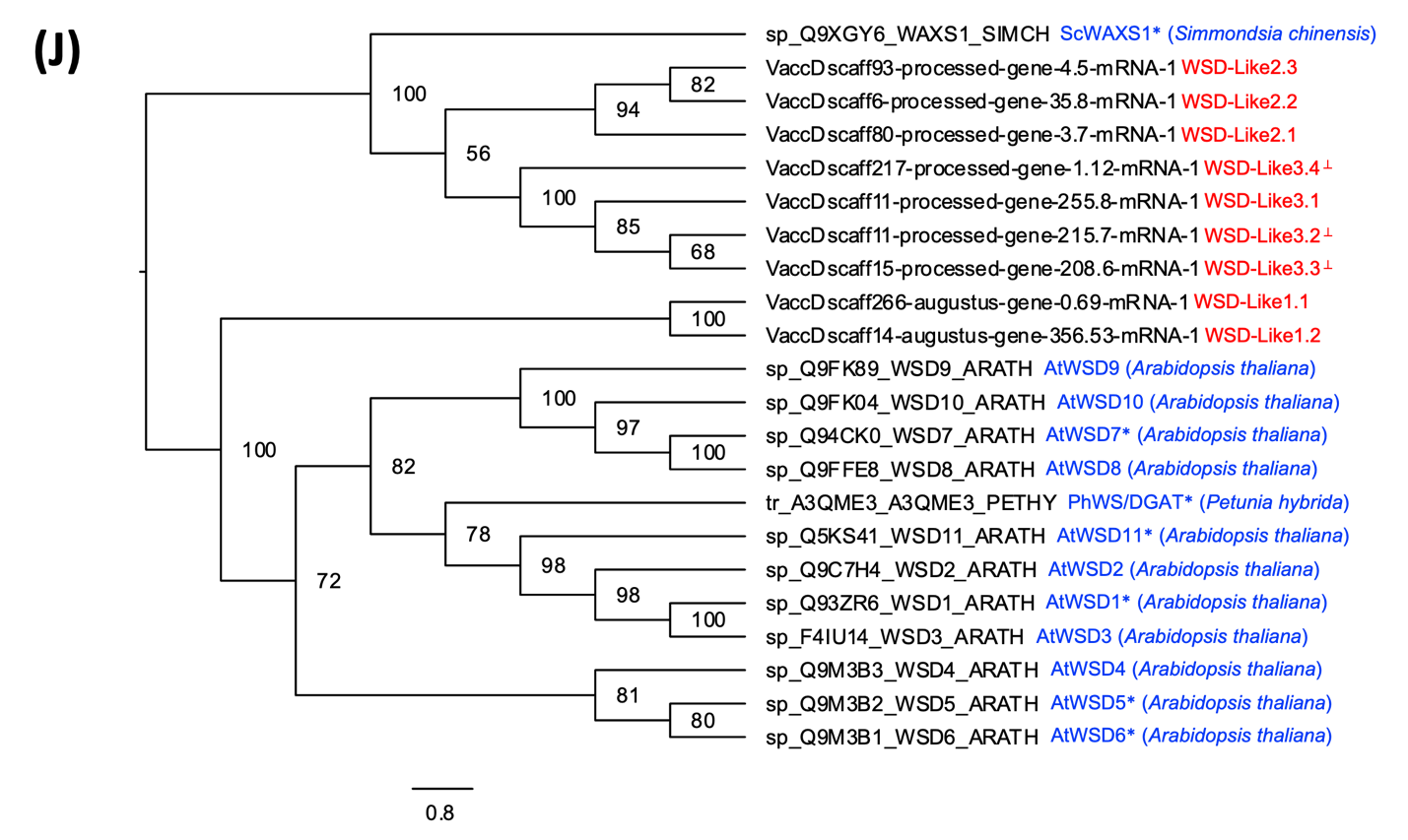

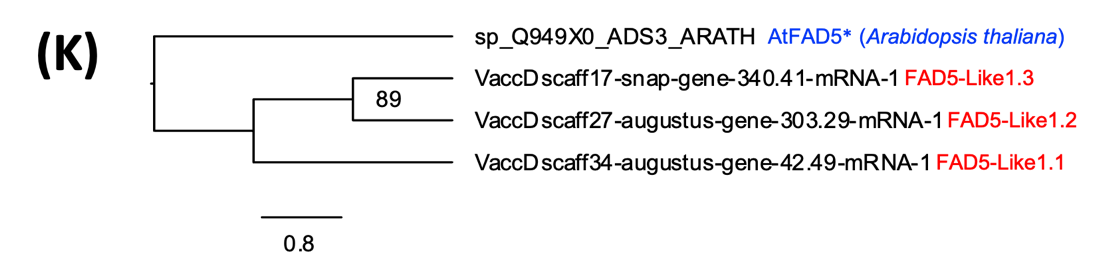

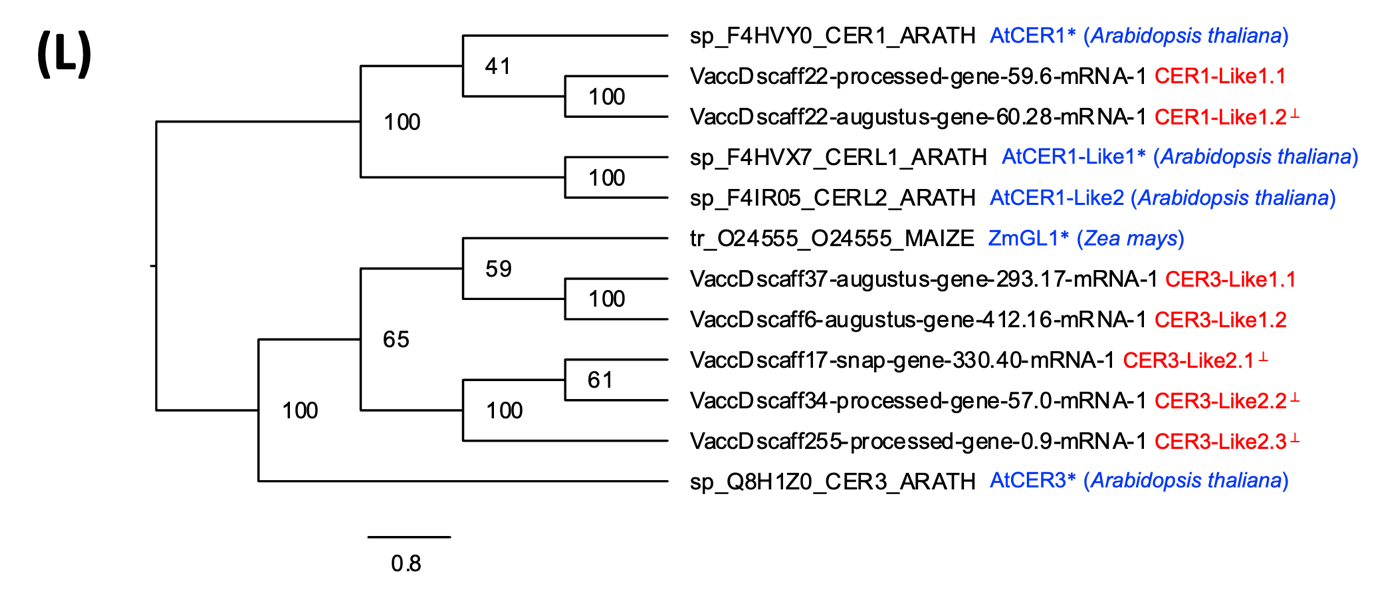

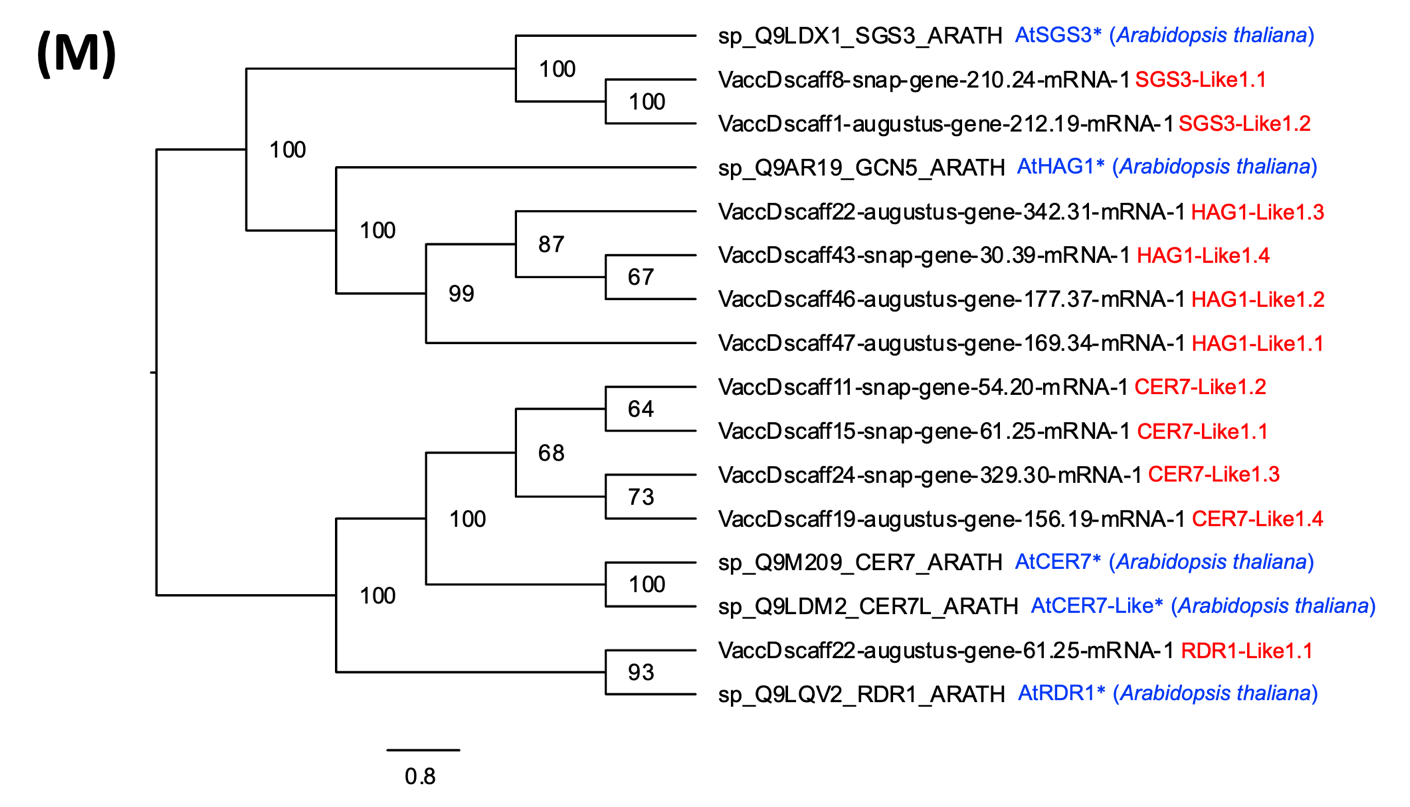

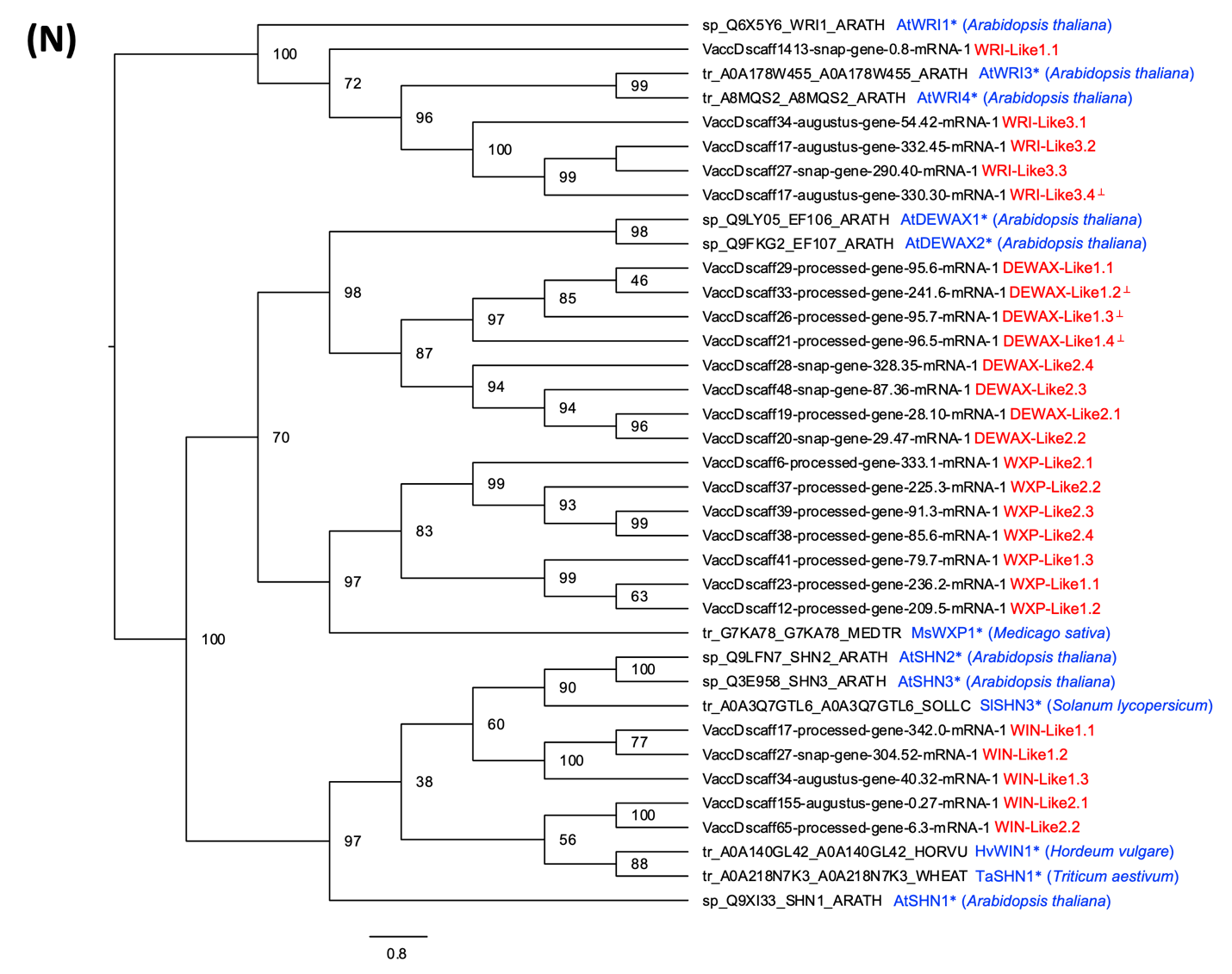

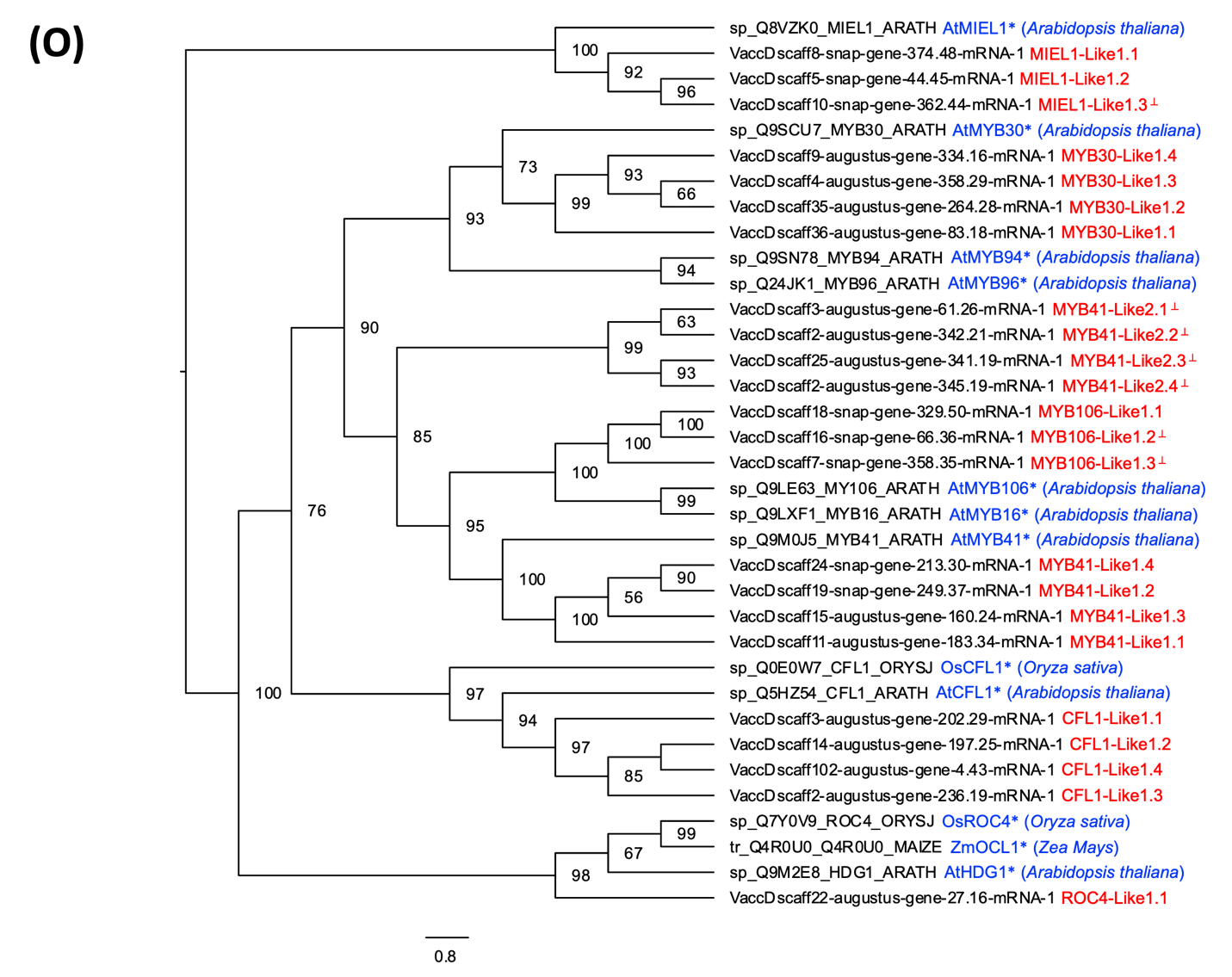

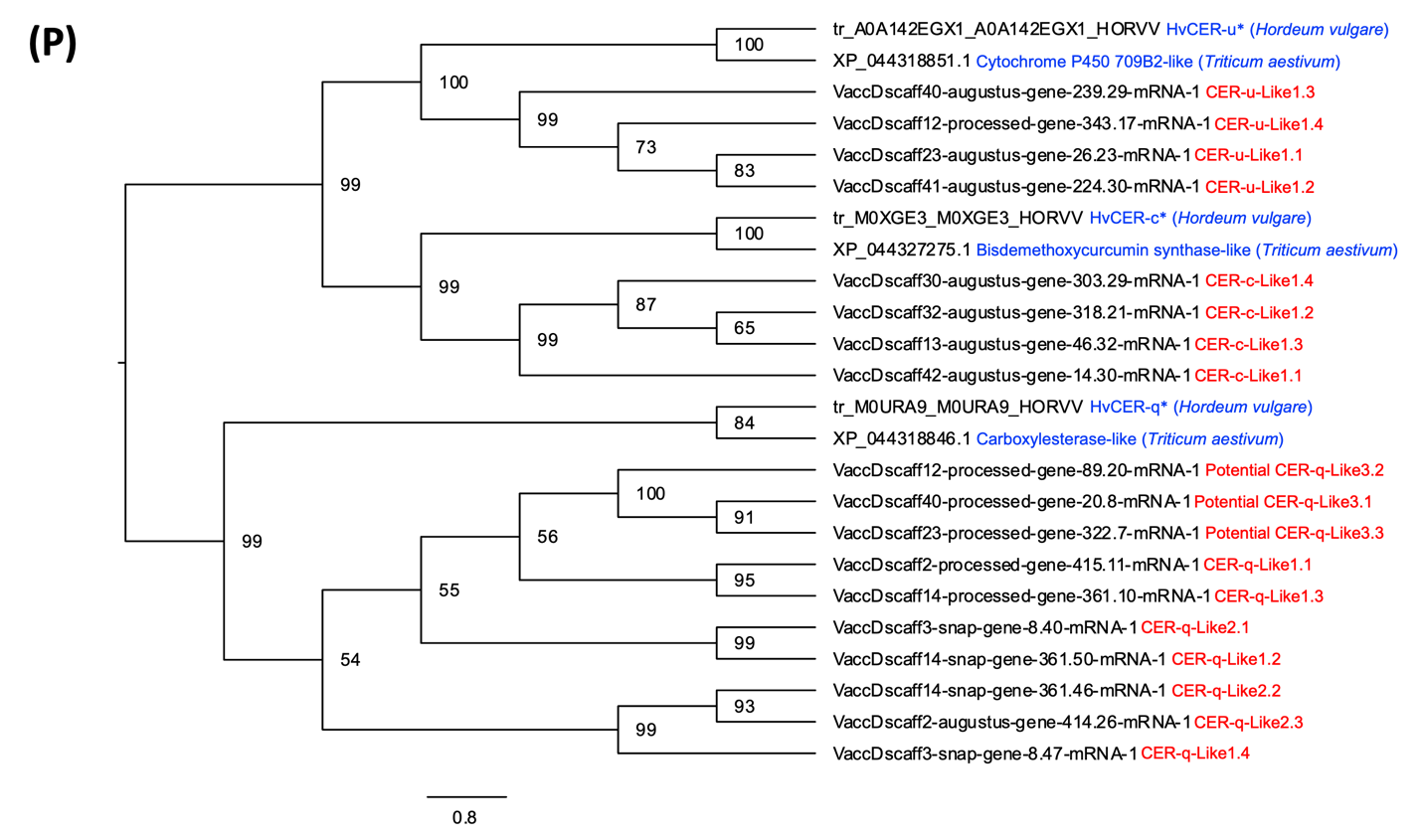

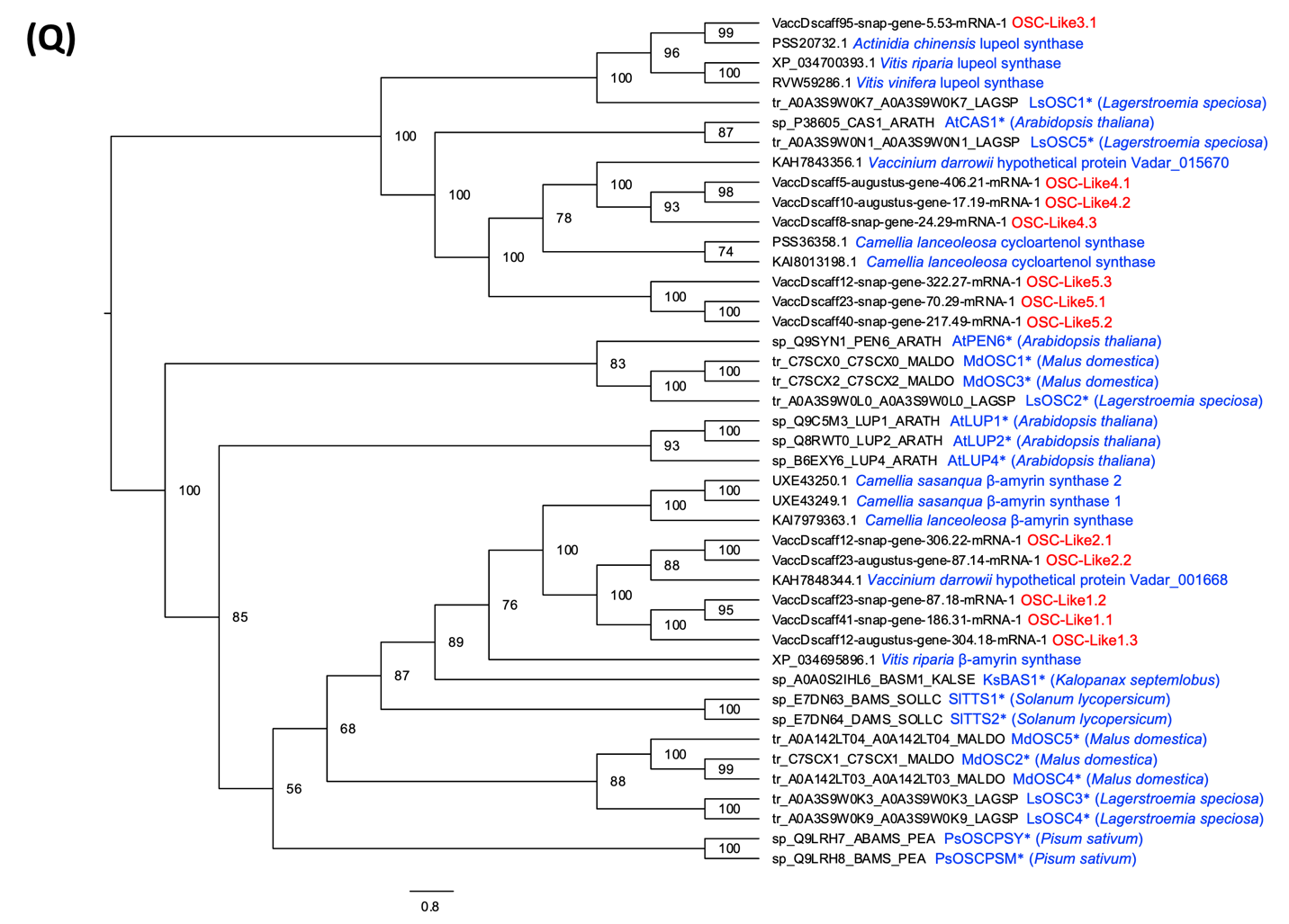

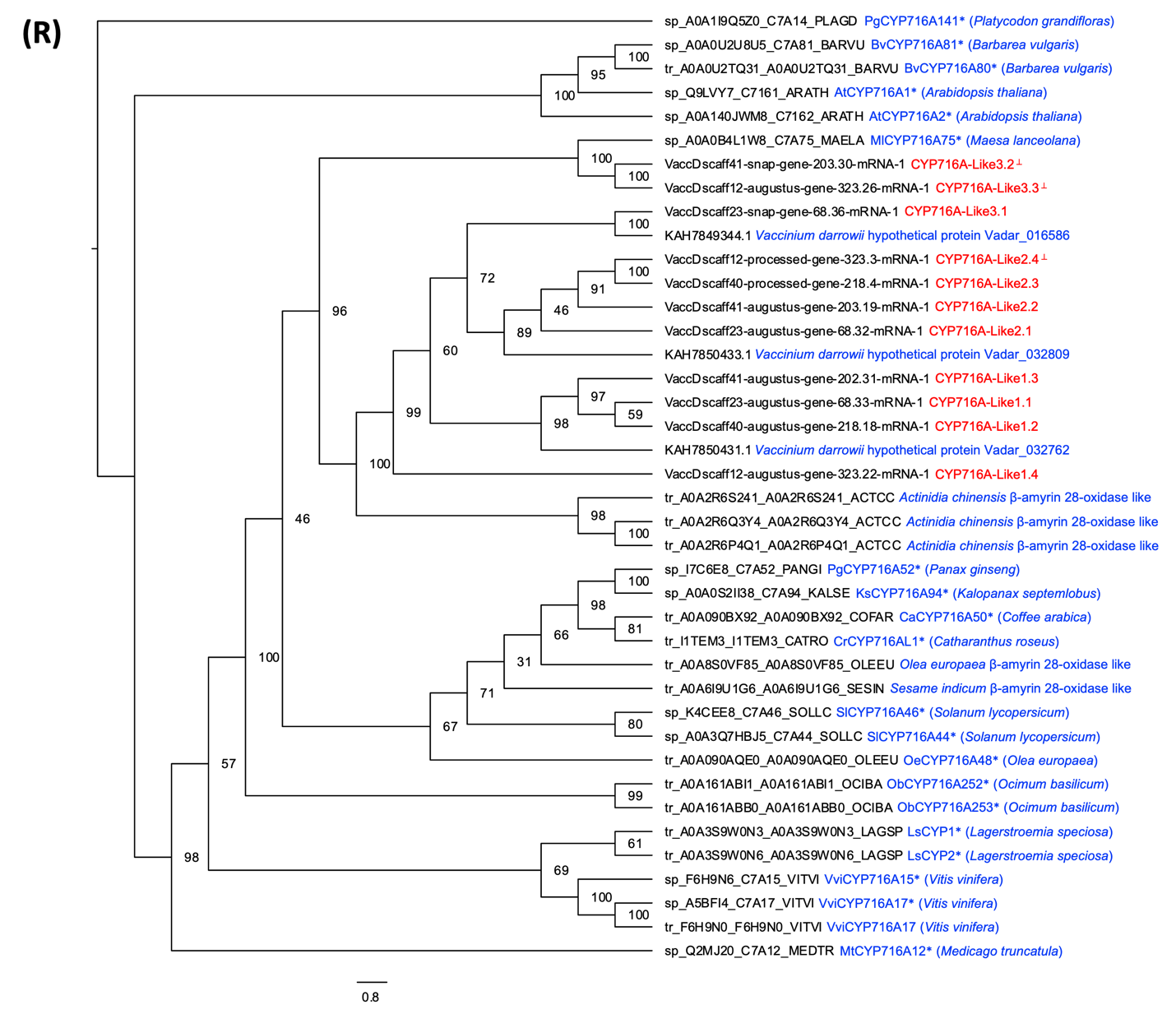

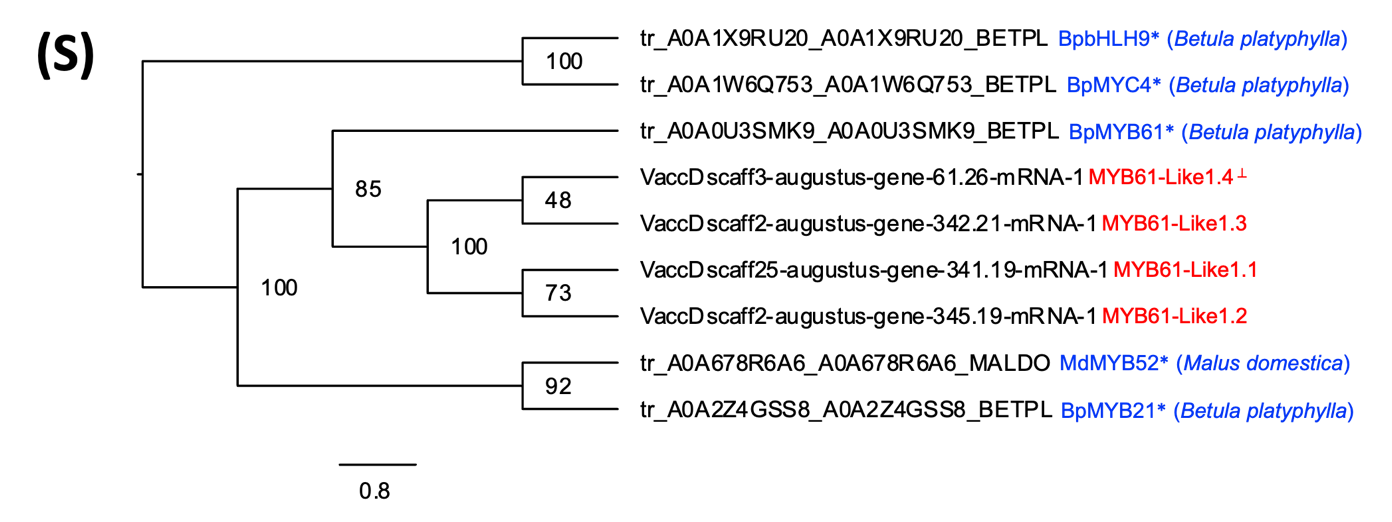

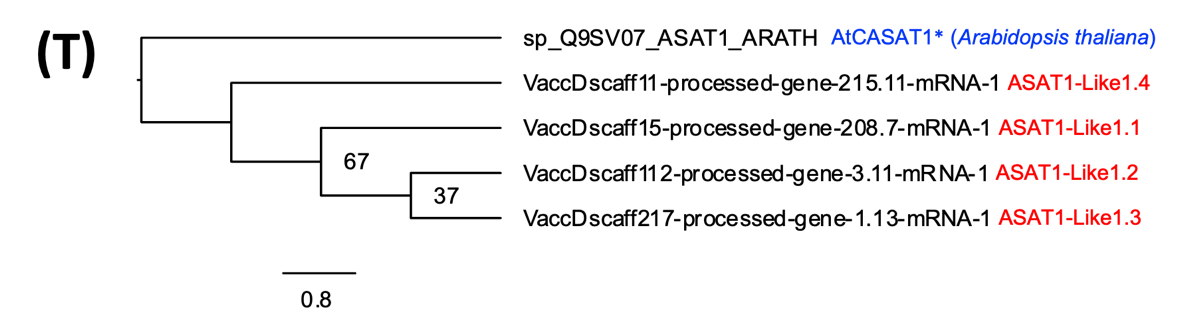


**Figure S4.** Phylogenetic relationships of putative wax-related genes in blueberries (highlighted by red text) and homologs in other species (highlighted by blue text). **(A)** Fatty acid synthase (FAS) complex including β-ketoacyl-ACP synthases (KASs), β-ketoacyl-ACP reductases (KARs), β-hydroxyacyl-ACP dehydrogenases (HADs), and trans-Δ^2^-enoyl-CoA reductases (EARs); **(B)** fatty acyl-ACP thioesterase B (FATB); **(C)** ATP-citrate synthase alpha chain protein 1 (ACLA-1); **(D)** long chain acyl-CoA synthetases (LACSs); **(E)** ABCG and LTPG transporters; **(F)** CER9 and PAS1 regulators; **(G)** β-ketoacyl-CoA synthases (KCSs); **(H)** β-ketoacyl-CoA reductases (KCRs), β-hydroxyacyl-CoA dehydrogenases (HCDs, encoded by *PAS2*), and trans-Δ^2^-enoyl-CoA reductases (ECRs, encoded by *CER10*); **(I)** fatty acyl-CoA reductases (FARs); **(J)** wax ester synthase/diacylglycerol acyltransferases (WSDs); **(K)** fatty acid desaturases (FAD5); **(L)** aldehyde- and alkane-forming genes *CER1* and *CER3*; **(M)** CER3 regulators; **(N)** AP2-domain containing transcription factors (TFs) for aliphatic compound biosynthesis; **(O)** MYB and other TFs for aliphatic compound biosynthesis; **(P)** β-diketone biosynthetic genes *CER-cqu*; **(Q)** oxidosqualene cyclases (OSCs); **(R)** CYP716A subfamily monooxygenases; **(S)** MYB TFs for cyclic compound biosynthesis; **(T)** acyl-CoA cholesterol acyltransferases (ASAT1). Potential *CER-q-Like3.1–3.3* in **(P)** are genes that are not selected as top candidates according to the workflow described in **Supplemental material 2** but have strong correlations with β-diketone contents. Numbers represent the bootstrap values for each node (1000 replicates). The asterisks identify functionally characterized genes in other species; the symbol “⊥” identify non-expressed genes in blueberries that are not presented in **Table S5**. Scale bars are shown in individual panels.


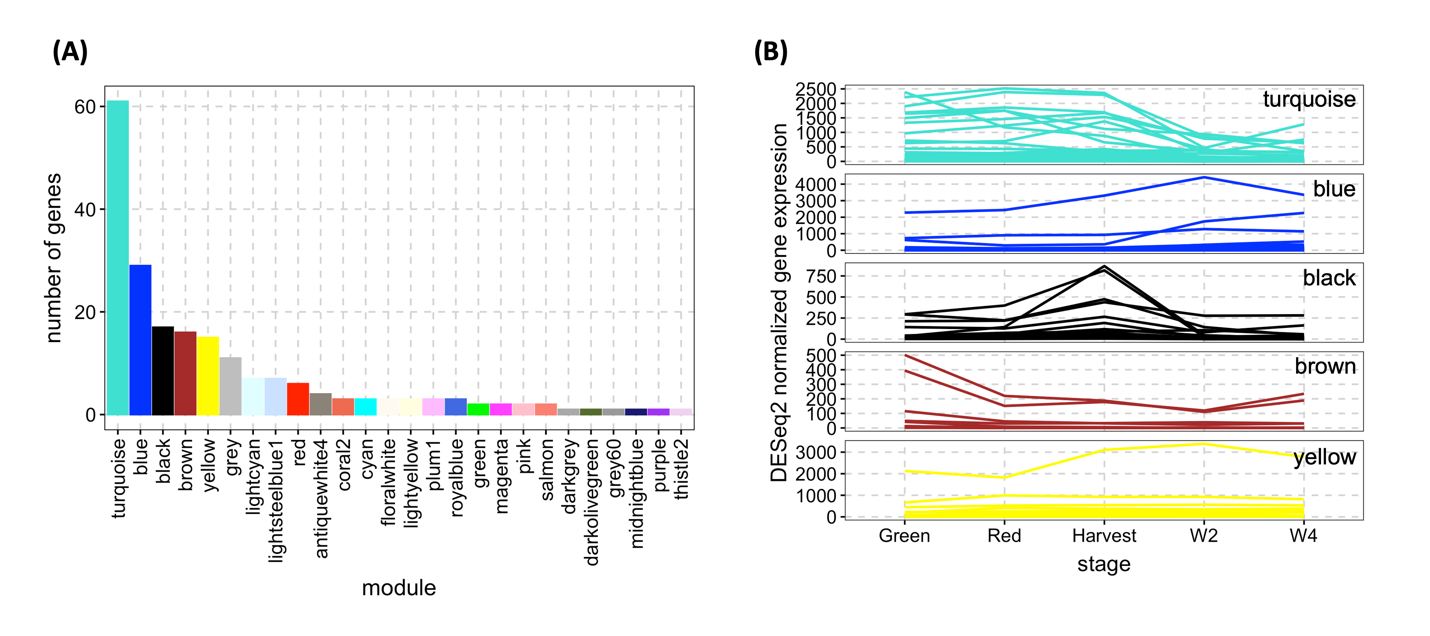


**Figure S5.** Weighted correlation network analysis (WCGNA) for co-expression of putative cuticular wax related genes during fruit ripening and postharvest storage in control (CN) treatment in ‘Calypso’. Each color module stands for a distinct expression pattern. **(A)** Histogram for the number of genes in each module; **(B)** expression patterns of genes in modules with the largest number of genes; data were DESeq2 normalized; the acronyms W2 and W4 stand for two and four weeks after postharvest storage, respectively.


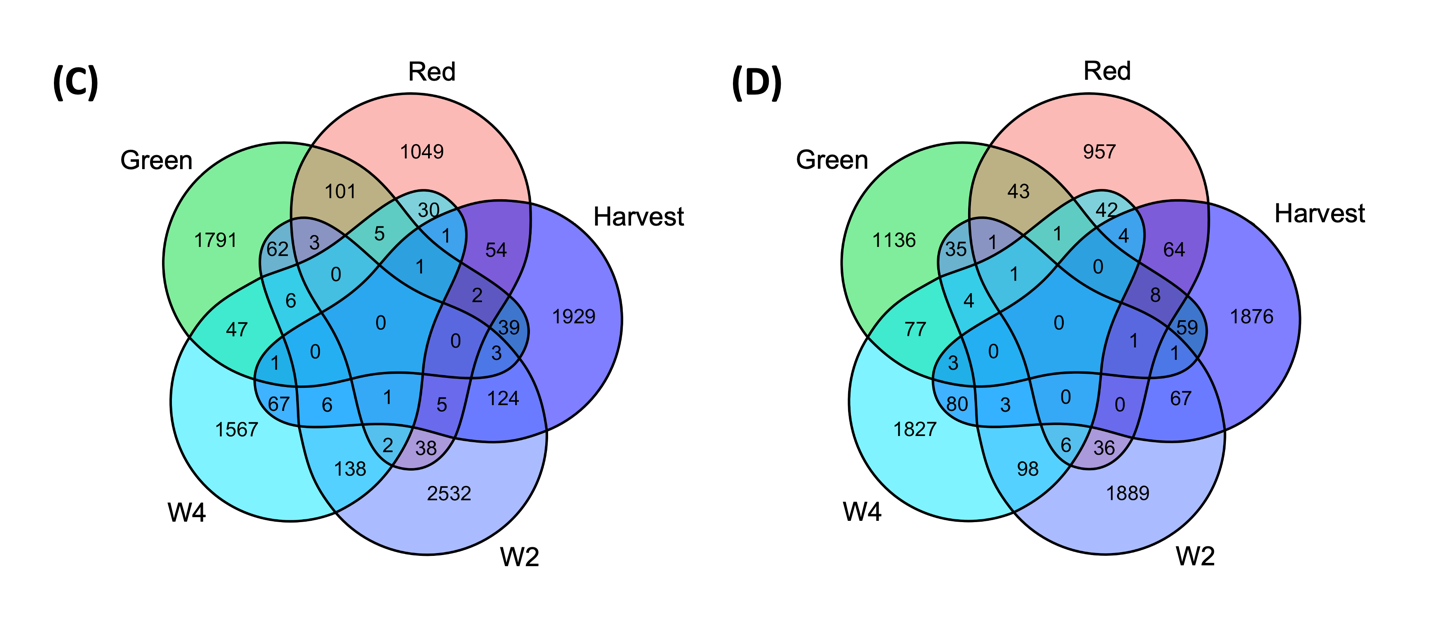


**Figure S6.** Venn diagrams displaying the number of differentially expressed genes (DEGs) between abscisic acid (ABA) and control (CN) treatments in ‘Calypso’ blueberries according to DEseq2 analysis. **(A)** Upregulated genes; **(B)** downregulated genes.


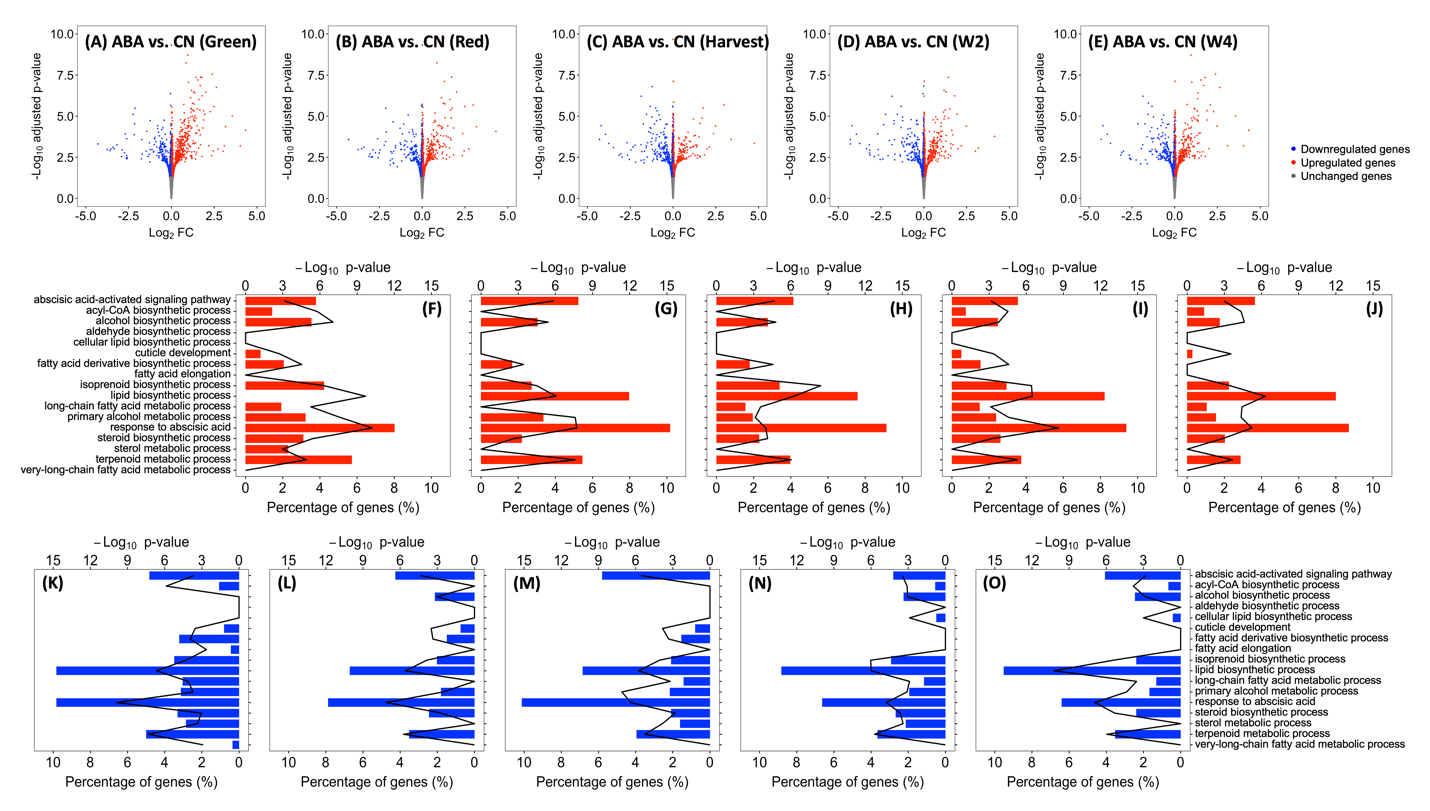


**Figure S7.** Differential gene expression analysis **(A–E)** between control (CN) and abscisic acid (ABA) treatment and enrichment analysis **(F–O)** in ‘Calypso’ blueberries. In each row from left to right, each panel represents the comparison between ABA and CN treatments at Green, Red, Harvest, W2, and W4 stage, respectively. **(A–E)** Volcano plots for differentially expressed genes (DEGs, adjusted *p*-value < 0.05 or log_2_ fold change [FC] < –2 or log_2_ FC > 2) between each comparison; blue dots refer to downregulated genes, red dots refer to upregulated genes, and grey dots refer to unaffected genes. **(F–O)** Enriched (false discovery rate [FDR] < 0.01) wax-related GO terms between each comparison within upregulated (**F–J)** and downregulated **(K–O)** genes; line graph indicates the **–**log_10_ *p*-value of each gene ontology (GO) term, bar graph indicates the percentage of upregulated or downregulated genes in each GO term; **–**log_10_ *p*-value = 0 and percentage of upregulated or downregulated genes = 0 identify non-enriched GO terms (FDR > 0.01).


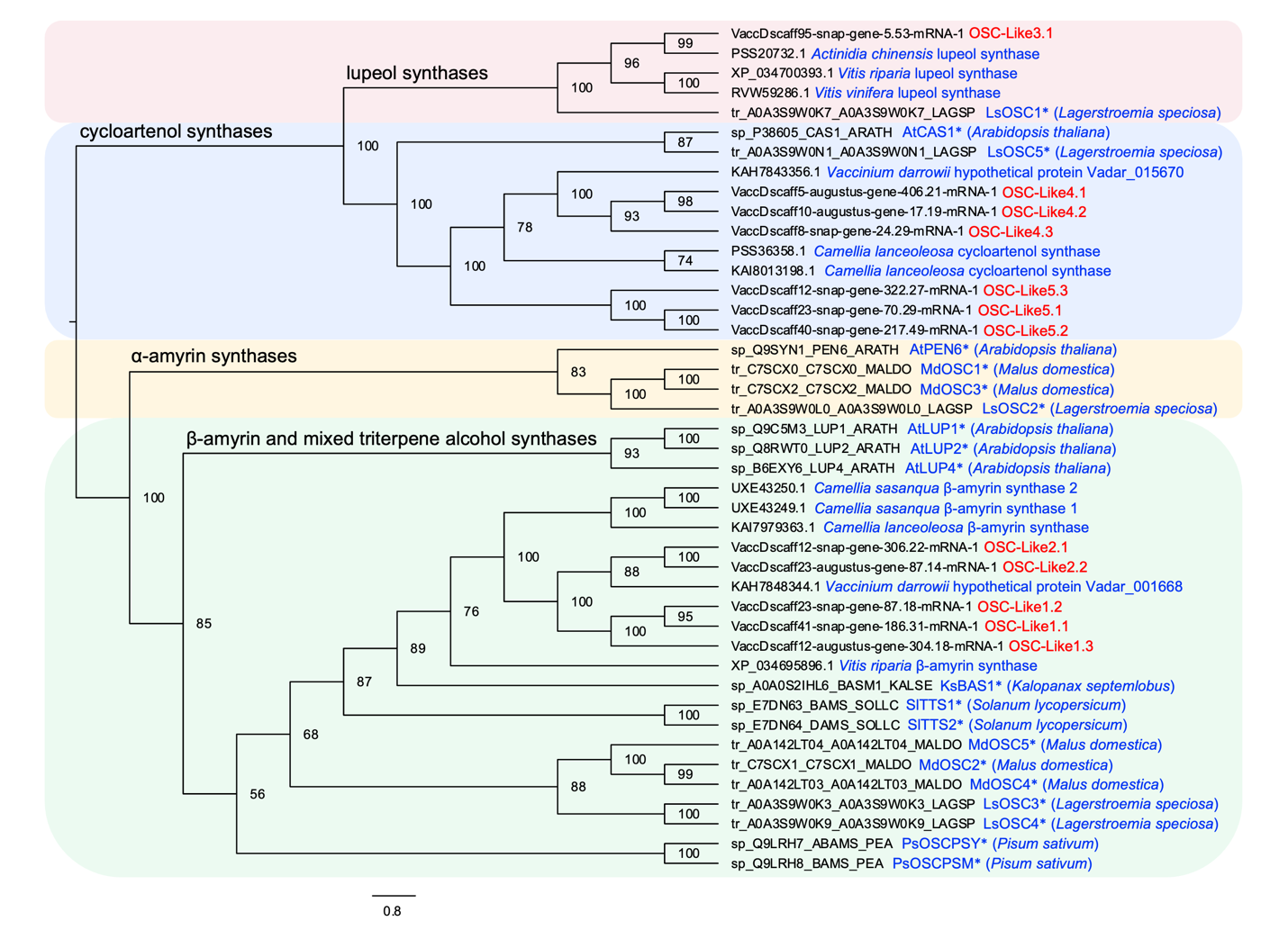


**Figure S8.** Phylogenetic relationships of putative oxidosqualene cyclases (OSCs) in blueberries (highlighted by red text) and functionally characterized or putative OSCs in other species (highlighted by blue text). The catalytic specificities of OSCs are highlighted by different color frames (pink: lupeol synthases; light blue: cycloartenol synthases; light yellow: α-amyrin synthases; light green: β-amyrin and mixed triterpene alcohol synthases). Numbers represent the bootstrap values for each node (1000 replicates). The asterisks indicate functionally characterized genes in other species.


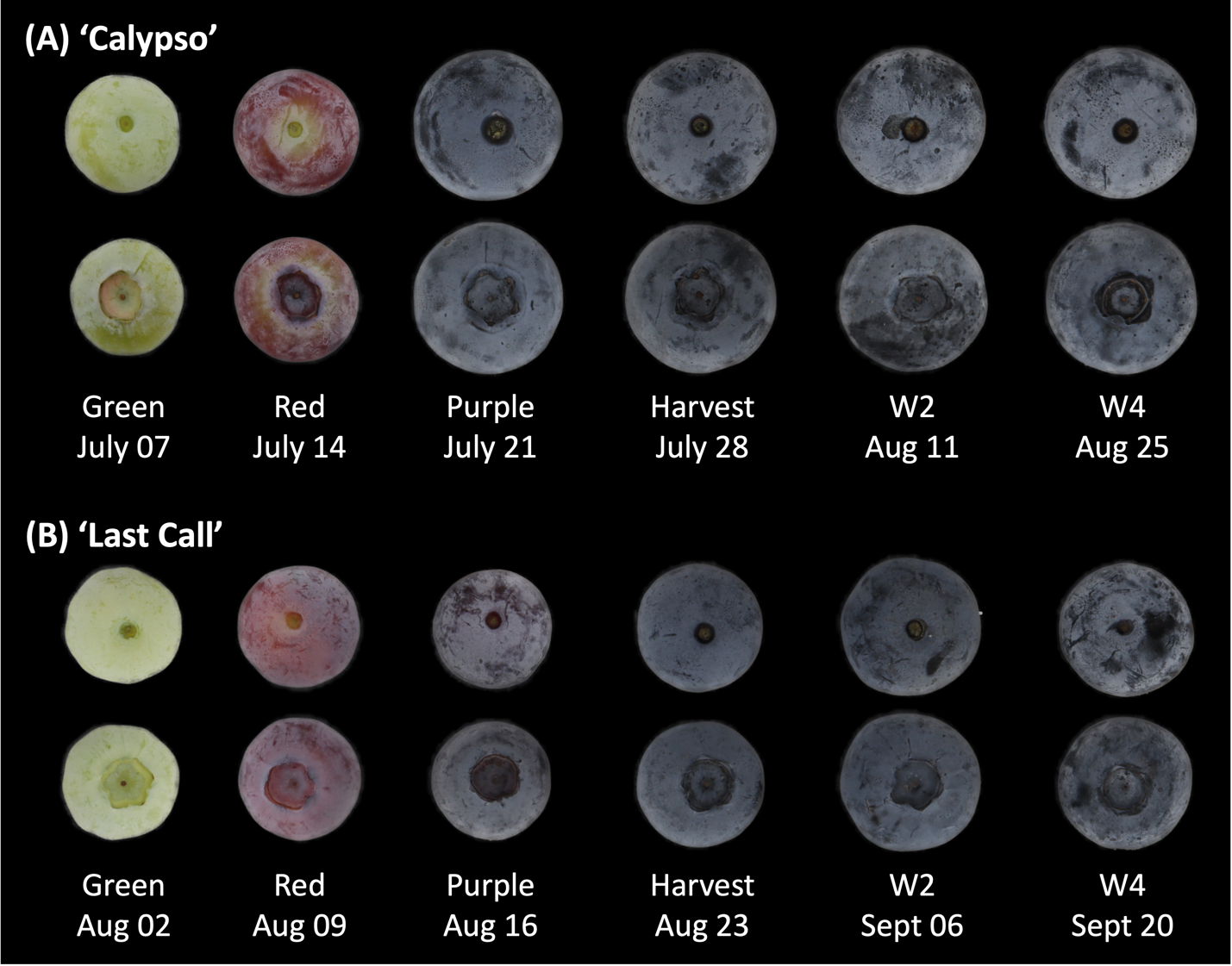


**Figure S9.** Blueberry sampling stages and corresponding dates in 2021 during fruit ripening and postharvest storage in ‘Calypso’ **(A)** and ‘Last Call’ **(B)**. “Green” berries were collected when there were 5–10% purple and blue fruit, and only uniformly green-colored berries were collected; “Red” berries were collected when there were 20–30% purple and blue fruit, and only uniformly red-colored berries were collected; “Purple” berries were collected when there were 40–50% purple and blue fruit, and only uniformly purple- and blue-colored berries were collected; “Harvest” berries were collected at the first commercial pick when there were 50–60% purple and blue fruit, and only uniformly purple- and blue-colored berries were collected. Berries for “W2” and “W4” analysis were collected at “Harvest” and stored at 0.5 °C and 95% RH for 2 and 4 weeks, respectively.
