# Supplementary material for: Cuticular wax biosynthesis in blueberries (Vaccinium corymbosum L.): Transcript and metabolite changes during ripening and storage affect key fruit quality traits
Source: Hortic Res. 2024 Jan 9;11(3):uhae004. doi: 10.1093/hr/uhae004 (PMC10923646; doi:10.1093/hr/uhae004)
Supplement: Web_Material_uhae004 [file web_material_uhae004.zip › Supplemental table 5 & 12.docx]

**Table S5** Annotated wax-related genes in other species.

| **Species** | **Gene id** | **Gene name** | **Protein length** | **Enzyme** | **Function in cuticular wax biosynthesis** | **References** |
| --- | --- | --- | --- | --- | --- | --- |
| *A. thaliana* | AT2G04540 | KAS1 | 461 | β-ketoacyl-ACP synthase | fatty acid biosynthesis | (Ewald et al., 2007)^1^ |
| *A. thaliana* | AT1G74960 | KAS2 | 541 | β-ketoacyl-ACP synthase | fatty acid biosynthesis | (Carlsson et al., 2002)^2^ |
| *A. thaliana* | AT1G62640 | KAS3 | 404 | β-ketoacyl-ACP synthase | fatty acid biosynthesis | (Tai et al., 1994)^3^ |
| *Spinacia oleracea* | SOVF_115730 | KAS1 | 461 | β-ketoacyl-ACP synthase | fatty acid biosynthesis | (Shimakata & Stumpf, 1982a)^4^ |
| *Spinacia oleracea* | SOVF_196450 | KAS2 | 542 | β-ketoacyl-ACP synthase | fatty acid biosynthesis | (Shimakata & Stumpf, 1982a)^4^ |
| *Spinacia oleracea* | SOVF_087070 | KAS3 | 405 | β-ketoacyl-ACP synthase | fatty acid biosynthesis | (Clough et al., 1992; Tai & Jaworski, 1993)^5,6^ |
| *Spinacia oleracea* | SOVF_029040 | KAR | 300 | β-ketoacyl-ACP reductase | fatty acid biosynthesis | (Shimakata & Stumpf, 1982b)^7^ |
| *Spinacia oleracea* | SOVF_091840 | KAR | 321 | β-ketoacyl-ACP reductase | fatty acid biosynthesis | (Shimakata & Stumpf, 1982b)^7^ |
| *Spinacia oleracea* | SOVF_085380 | KAR | 320 | β-ketoacyl-ACP reductase | fatty acid biosynthesis | (Shimakata & Stumpf, 1982b)^7^ |
| *Spinacia oleracea* | SOVF_008870 | EAR | 393 | trans-∆2-enoyl-ACP reductase | fatty acid biosynthesis | (Shimakata & Stumpf, 1982b)^7^ |
| *A. thaliana* | T26I12.190 | KAR | 298 | β-ketoacyl-ACP reductase | fatty acid biosynthesis | NA |
| *A. thaliana* | AT2G22230 | HAD | 220 | β-hydroxyacyl-ACP dehydratase | fatty acid biosynthesis | (Brown et al., 2009)^8^ |
| *A. thaliana* | AT2G05990 | EAR | 390 | trans-∆2-enoyl-ACP reductase | fatty acid biosynthesis | NA |
| *A. thaliana* | AT1G10670 | ACLA-1 | 443 | ATP-citrate synthase | fatty acid biosynthesis | (Chen et al., 2017)^9^ |
| *A. thaliana* | AT1G08510 | FATB | 412 | fatty acyl-ACP thioesterase B | fatty acid biosynthesis | (Bonaventure et al., 2003)^10^ |
| *A. thaliana* | AT2G47240 | LACS1 \| CER8 | 660 | long-chain acyl-CoA synthetase | fatty acid biosynthesis | (Lü et al., 2009; Zhao et al., 2010)^11,12^ |
| *A. thaliana* | AT1G49430 | LACS2 | 665 | long-chain acyl-CoA synthetase | fatty acid biosynthesis | (Schnurr et al., 2004)^13^ |
| *A. thaliana* | AT1G64400 | LACS3 | 665 | long-chain acyl-CoA synthetase | fatty acid biosynthesis | (Shockey et al., 2002)^14^ |
| *A. thaliana* | AT4G23850 | LACS4 | 666 | long-chain acyl-CoA synthetase | fatty acid biosynthesis | (Zhao et al., 2010)^12^ |
| *A. thaliana* | AT3G05970 | LACS6 | 701 | long-chain acyl-CoA synthetase | fatty acid biosynthesis | (Fulda et al., 2002, 2004)^15,16^ |
| *A. thaliana* | AT5G27600 | LACS7 | 700 | long-chain acyl-CoA synthetase | fatty acid biosynthesis | (Fulda et al., 2002, 2004)^15,16^ |
| *A. thaliana* | AT2G04350 | LACS8 | 720 | long-chain acyl-CoA synthetase | fatty acid biosynthesis | (Zhao et al., 2010)^12^ |
| *A. thaliana* | AT1G77590 | LACS9 | 691 | long-chain acyl-CoA synthetase | fatty acid biosynthesis | (Zhao et al., 2010)^12^ |
| *A. thaliana* | AT4G34100 | CER9 | 1108 | E3 ubiquitin ligase interacting with LACS1 | regulator for fatty acid biosynthesis | (Lü et al., 2012)^17^ |
| *A. thaliana* | AT1G01120 | KCS1 | 528 | β-keto acyl-CoA synthase | fatty acid elongation | (Todd et al., 1999)^18^ |
| *A. thaliana* | AT1G04220 | KCS2 | 528 | β-keto acyl-CoA synthase | fatty acid elongation | (Lee et al., 2009b)^19^ |
| *A. thaliana* | AT1G07720 | KCS3 | 478 | β-keto acyl-CoA synthase | fatty acid elongation | (Huang et al., 2023)^20^ |
| *A. thaliana* | AT1G19440 | KCS4 | 516 | β-keto acyl-CoA synthase | fatty acid elongation | (Kim et al., 2021)^21^ |
| *A. thaliana* | AT1G25450 | KCS5 \| CER60 | 492 | β-keto acyl-CoA synthase | fatty acid elongation | (Fiebig et al., 2000)^22^ |
| *A. thaliana* | AT1G68530 | KCS6 \| CER6 \| CUT1 | 497 | β-keto acyl-CoA synthase | fatty acid elongation | (Millar et al., 1999)^23^ |
| *A. thaliana* | AT1G71160 | KCS7 | 460 | β-keto acyl-CoA synthase | fatty acid elongation | NA |
| *A. thaliana* | AT2G15090 | KCS8 | 481 | β-keto acyl-CoA synthase | fatty acid elongation | NA |
| *A. thaliana* | AT2G16280 | KCS9 | 512 | β-keto acyl-CoA synthase | fatty acid elongation | (Kim et al., 2013)^24^ |
| *A. thaliana* | AT2G26250 | KCS10 | 550 | β-keto acyl-CoA synthase | fatty acid elongation | NA |
| *A. thaliana* | AT2G26640 | KCS11 | 509 | β-keto acyl-CoA synthase | fatty acid elongation | NA |
| *A. thaliana* | AT2G28630 | KCS12 | 476 | β-keto acyl-CoA synthase | fatty acid elongation | (Chai et al., 2021; Huang et al., 2023)^20,25^ |
| *A. thaliana* | AT2G46720 | KCS13 | 466 | β-keto acyl-CoA synthase | fatty acid elongation | NA |
| *A. thaliana* | AT3G10280 | KCS14 | 459 | β-keto acyl-CoA synthase | fatty acid elongation | NA |
| *A. thaliana* | AT3G52160 | KCS15 | 451 | β-keto acyl-CoA synthase | fatty acid elongation | NA |
| *A. thaliana* | AT4G34250 | KCS16 | 493 | β-keto acyl-CoA synthase | fatty acid elongation | (Hegebarth et al., 2017)^26^ |
| *A. thaliana* | AT4G34510 | KCS17 | 487 | β-keto acyl-CoA synthase | fatty acid elongation | NA |
| *A. thaliana* | AT4G34520 | KCS18 \| FAE1 | 506 | β-keto acyl-CoA synthase | fatty acid elongation | (Jasinski et al., 2012)^27^ |
| *A. thaliana* | AT5G43760 | KCS19 | 464 | β-keto acyl-CoA synthase | fatty acid elongation | (Huang et al., 2023)^20^ |
| *A. thaliana* | AT5G43760 | KCS20 | 529 | β-keto acyl-CoA synthase | fatty acid elongation | (Lee et al., 2009b)^19^ |
| *A. thaliana* | AT5G49070 | KCS21 | 464 | β-keto acyl-CoA synthase | fatty acid elongation | NA |
| *A. thaliana* | AT4G24510 | CER2 | 421 | β-keto acyl-CoA synthase | fatty acid elongation | (Haslam et al., 2012)^28^ |
| *A. thaliana* | AT4G13840 | CER26 \| CER2-Like1 | 428 | β-keto acyl-CoA synthase | fatty acid elongation | (Pascal et al., 2013)^29^ |
| *A. thaliana* | AT3G23840 | CER26-Like \| CER2-Like2 | 420 | β-keto acyl-CoA synthase | fatty acid elongation | (Pascal et al., 2013)^29^ |
| *Zea mays* | ZEAMMB73_Zm00001d002353 | GL2 | 395 | β-keto acyl-CoA synthase | fatty acid elongation | (Alexander et al., 2020)^30^ |
| *Zea mays* | ZEAMMB73_Zm00001d024317 | GL2-Like | 455 | β-keto acyl-CoA synthase | fatty acid elongation | (Alexander et al., 2020)^30^ |
| *Solanum lycopersicum* | NM_001317822 | CER6 | 496 | β-keto acyl-CoA synthase | fatty acid elongation | (Leide et al., 2007)^31^ |
| *Gossypium hirsutum* | XM_016852015 | KCS13 | 492 | β-keto acyl-CoA synthase | fatty acid elongation | (Qin et al., 2007)^32^ |
| *A. thaliana* | AT1G67730 | KCR1 | 318 | β-keto acyl-CoA reductase | fatty acid elongation | (Beaudoin et al., 2009)^33^ |
| *A. thaliana* | AT1G24470 | KCR2 | 312 | β-keto acyl-CoA reductase | fatty acid elongation | (Beaudoin et al., 2009)^33^ |
| *Zea mays* | ZEAMMB73_Zm00001d017111 | GL8 | 326 | β-keto acyl-CoA reductase | fatty acid elongation | (Dietrich et al., 2005)^34^ |
| *Zea mays* | ZEAMMB73_Zm00001d050992 | GL8B | 326 | β-keto acyl-CoA reductase | fatty acid elongation | (Dietrich et al., 2005)^34^ |
| *A. thaliana* | AT3G54010 | PAS1 | 635 | immunophillin-like protein | regulator for fatty acid elongation | (Carol et al., 2001)^35^ |
| *A. thaliana* | AT5G10480 | PAS2 | 230 | β-hydroxyacyl-CoA dehydratase | fatty acid elongation | (Bach et al., 2008)^36^ |
| *A. thaliana* | AT3G55360 | CER10 | 310 | trans-∆2-enoyl-CoA reductase | fatty acid elongation | (Zheng et al., 2005)^37^ |
| *A. thaliana* | AT5G22500 | FAR1 | 491 | fatty acyl-CoA reductase | acyl-reducation branch | (Kosma et al., 2012)^38^ |
| *A. thaliana* | AT3G11980 | FAR2 | 616 | fatty acyl-CoA reductase | acyl-reducation branch | NA |
| *A. thaliana* | AT4G33790 | FAR3 \| CER4 | 493 | fatty acyl-CoA reductase | acyl-reducation branch | (Rowland et al., 2006)^39^ |
| *A. thaliana* | AT3G44540 | FAR4 | 493 | fatty acyl-CoA reductase | acyl-reducation branch | (Kosma et al., 2012)^38^ |
| *A. thaliana* | AT3G44550 | FAR5 | 496 | fatty acyl-CoA reductase | acyl-reducation branch | (Kosma et al., 2012)^38^ |
| *A. thaliana* | AT3G56700 | FAR6 | 548 | fatty acyl-CoA reductase | acyl-reducation branch | (Gupta et al., 2012)^40^ |
| *Triticum aestivum* | XM_044509396 | FAR1 | 525 | fatty acyl-CoA reductase | acyl-reducation branch | (Wang et al., 2015)^41^ |
| *A. thaliana* | AT5G37300 | WSD1 | 481 | wax ester synthase (fatty acyl-coA: fatty alcohol acyltransferase) | acyl-reducation branch | (Li et al., 2008; Patwari et al., 2019)^42,43^ |
| *A. thaliana* | AT3G49200 | WSD5 | 507 | wax ester synthase (fatty acyl-coA: fatty alcohol acyltransferase) | acyl-reducation branch | (Salewski, 2022)^44^ |
| *A. thaliana* | AT3G49210 | WSD6 | 518 | wax ester synthase (fatty acyl-coA: fatty alcohol acyltransferase) | acyl-reducation branch | (Patwari et al., 2019; Salewski, 2022)^43,44^ |
| *A. thaliana* | AT5G12420 | WSD7 | 480 | wax ester synthase (fatty acyl-coA: fatty alcohol acyltransferase) | acyl-reducation branch | (Patwari et al., 2019; Salewski, 2022)^43,44^ |
| *A. thaliana* | AT5G53390 | WSD11 | 486 | wax ester synthase (fatty acyl-coA: fatty alcohol acyltransferase) | acyl-reducation branch | (Salewski, 2022)^44^ |
| *Petunia hydrida* | DQ093641 | WS/DGAT | 521 | wax ester synthase (fatty acyl-coA: fatty alcohol acyltransferase) | acyl-reducation branch | (King et al., 2007)^45^ |
| *Simmondsia chinensis* | AF149919 | WAXS1 | 352 | wax ester synthase (fatty acyl-coA: fatty alcohol acyltransferase) | acyl-reducation branch | (Lardizabal et al., 2000)^46^ |
| *A. thaliana* | AT1G06350 | CER17 \| ADS4 | 300 | fatty acyl-CoA desaturase | unsaturated alcohol biosynthesis | (Yang et al., 2017)^47^ |
| *A. thaliana* | AT3G15850 | FAD5 \| ADS3 | 371 | fatty acyl-CoA desaturase | unsaturated fatty acid biosynthesis | (Heilmann et al., 2004)^48^ |
| *A. thaliana* | AT5G57800 | CER3 \| WAX2 | 632 | fatty acid reductase | decarbonylation branch | (Hannoufa et al., 1996; Chen et al., 2003; Rowland et al., 2007; Bernard et al., 2012)^49–52^ |
| *Zea mays* | ZEAMMB73_Zm00001d020557 | GL1 | 621 | fatty acid reductase | decarbonylation branch | (Sturaro et al., 2005)^53^ |
| *A. thaliana* | AT3G60500 | CER7 | 438 | 3',5'-exoribonuclease | regulator for decarbonylation branch | (Hooker et al., 2007)^54^ |
| *A. thaliana* | AT5G23570 | SGS3 | 625 | mRNA-mediated gene silencing protein | regulator for decarbonylation branch | (Lam et al., 2012)^55^ |
| *A. thaliana* | AT1G14790 | RDR1 | 1107 | mRNA-mediated gene silencing protein | regulator for decarbonylation branch | (Lam et al., 2012)^55^ |
| *A. thaliana* | AT3G54610 | HAG1 | 568 | histone acylation protein | regulator for decarbonylation branch | (Wang et al., 2018)^56^ |
| *A. thaliana* | AT1G55270 | SAGL1 | 434 | E3 ubiquitin ligase interacting with CER3 | regulator for decarbonylation branch | (Kim et al., 2019)^57^ |
| *A. thaliana* | AT1G02205 | CER1 \| CER22 | 625 | aldehyde decarbonylase | decarbonylation branch | (Bernard et al., 2012; Sakuradani et al., 2013)^50,58^ |
| *A. thaliana* | AT1G02190 | CER1-Like1 | 627 | aldehyde decarbonylase | decarbonylation branch | (Pascal et al., 2019)^59^ |
| *Solanum lycopersicum* | SOLYC03G065250 | CER1 | 374 | aldehyde decarbonylase | decarbonylation branch | (Wu et al., 2022)^60^ |
| *A. thaliana* | AT1G57750 | MAH \| CYP96A15 | 497 | mid-chain alkane hydroxylase | decarbonylation branch | (Greer et al., 2007)^61^ |
| *A. thaliana* | AT1G27950 | LTPG1 | 193 | lipid transfer protein | aliphatic compound transport and secretion | (Lee et al., 2009a)^62^ |
| *A. thaliana* | AT3G43720 | LTPG2 | 193 | lipid transfer protein | aliphatic compound transport and secretion | (Kim et al., 2012)^63^ |
| *A. thaliana* | AT1G17840 | ABCG11 | 703 | adenosine triphosphate binding cassette (ABC) transporter | aliphatic compound transport and secretion | (Bird et al., 2007)^64^ |
| *A. thaliana* | AT1G51500 | ABCG12 \| CER5 | 687 | adenosine triphosphate binding cassette (ABC) transporter | aliphatic compound transport and secretion | (Pighin et al., 2004)^65^ |
| *A. thaliana* | AT1G51460 | ABCG13 | 678 | adenosine triphosphate binding cassette (ABC) transporter | aliphatic compound transport and secretion | (Panikashvili et al., 2011)^66^ |
| *Brassica napus* | BNU22105 | LTP1 | 109 | lipid transfer protein | aliphatic compound transport and secretion | (Liu et al., 2014)^67^ |
| *A. thaliana* | AT1G15360 | SHN1 \| WIN1 | 199 | AP2 domain-containing transcription fator | regulator for aliphatic compound biosynthesis | (Aharoni et al., 2004; Broun et al., 2004; Kannangara et al., 2007)^68–70^ |
| *A. thaliana* | AT5G11190 | SHN2 \| WIN2 | 189 | AP2 domain-containing transcription fator | regulator for aliphatic compound biosynthesis | (Shi et al., 2011)^71^ |
| *A. thaliana* | AT5G25390 | SHN3 \| WIN3 | 189 | AP2 domain-containing transcription fator | regulator for aliphatic compound biosynthesis | (Shi et al., 2011)^71^ |
| *A. thaliana* | AT3G54320 | WRI1 | 430 | AP2 domain-containing transcription fator | regulator for aliphatic compound biosynthesis | (Cernac & Benning, 2004)^72^ |
| *A. thaliana* | AT1G16060 | WRI3 | 345 | AP2 domain-containing transcription fator | regulator for aliphatic compound biosynthesis | (To et al., 2013)^73^ |
| *A. thaliana* | AT1G79700 | WRI4 | 313 | AP2 domain-containing transcription fator | regulator for aliphatic compound biosynthesis | (To et al., 2013; Park et al., 2016)^73,74^ |
| *Hordeum vulgare* | KT946819 | WIN1 \| NUD | 206 | AP2 domain-containing transcription fator | regulator for aliphatic compound biosynthesis | (Taketa et al., 2008)^75^ |
| *Triticum aestivum* | KY807151 | SHN1 | 161 | AP2 domain-containing transcription fator | regulator for aliphatic compound biosynthesis | (Bi et al., 2018)^76^ |
| *Solanum lycopersicum* | NM_001319202.1 | SHN3 | 200 | AP2 domain-containing transcription fator | regulator for aliphatic compound biosynthesis | (Shi et al., 2013)^77^ |
| *Medicago sativa* | MTR_5G062700 | WXP1 | 371 | AP2 domain-containing transcription fator | regulator for aliphatic compound biosynthesis | (Zhang et al., 2005)^78^ |
| *A. thaliana* | AT5G07580 | DEWAX1 | 207 | AP2 domain-containing transcription fator | regulator for aliphatic compound biosynthesis | (Go et al., 2014)^79^ |
| *A. thaliana* | AT5G61590 | DEWAX2 | 201 | AP2 domain-containing transcription fator | regulator for aliphatic compound biosynthesis | (Kim et al., 2018)^80^ |
| *A. thaliana* | AT3G28910 | MYB30 | 323 | MYB transcription factor | regulator for aliphatic compound biosynthesis | (Raffaele et al., 2008)^81^ |
| *A. thaliana* | AT5G18650 | MIEL1 | 267 | E3 ubiquitin ligase inteacting with MYB30 | regulator for aliphatic compound biosynthesis | (Marino et al., 2013)^82^ |
| *A. thaliana* | AT4G28110 | MYB41 | 282 | MYB transcription factor | regulator for aliphatic compound biosynthesis | (Cominelli et al., 2008)^83^ |
| *A. thaliana* | AT3G47600 | MYB94 | 333 | MYB transcription factor | regulator for aliphatic compound biosynthesis | (Lee & Suh, 2015; Lee et al., 2016)^84,85^ |
| *A. thaliana* | AT5G62470 | MYB96 | 352 | MYB transcription factor | regulator for aliphatic compound biosynthesis | (Seo et al., 2009, 2011)^86,87^ |
| *A. thaliana* | AT5G15310 | MYB16 | 326 | MYB transcription factor | regulator for aliphatic compound biosynthesis | (Oshima et al., 2013)^88^ |
| *A. thaliana* | AT3G01140 | MYB106 | 345 | MYB transcription factor | regulator for aliphatic compound biosynthesis | (Oshima et al., 2013)^88^ |
| *Oryza sativa* | LOC_OS02G45780 | DHS | 165 | E3 ubiquitin ligase interacting with ROC4 | regulator for aliphatic compound biosynthesis | (Wang et al., 2018)^89^ |
| *Oryza sativa* | OSNPB_040569100 | ROC4 | 813 | homeodomain-leucine zipper IV protein | regulator for aliphatic compound biosynthesis | (Wang et al., 2018)^89^ |
| *Oryza sativa* | OSNPB_020516400 | CFL1 | 274 | WW domain protein | regulator for aliphatic compound biosynthesis | (Wu et al., 2011)^90^ |
| *A. thaliana* | AT2G33510 | CFL1 | 189 | WW domain protein | regulator for aliphatic compound biosynthesis | (Wu et al., 2011)^90^ |
| *A. thaliana* | AT3G61150 | HDG1 | 808 | homeodomain-leucine zipper IV protein | regulator for aliphatic compound biosynthesis | (Wu et al., 2011)^90^ |
| *Zea mays* | ZEAMMB73_Zm00001d040090 | OCL1 | 803 | homeodomain-leucine zipper IV protein | regulator for aliphatic compound biosynthesis | (Javelle et al., 2010)^91^ |
| *Hordeum vulgare* | MLOC_59804 | Eceriferum-c | 394 | chalcone synthase-like polyketide synthase | β-diketone biosynthesis | (Schneider et al., 2016; Hen-Avivi et al., 2016)^92,93^ |
| *Hordeum vulgare* | MLOC_13397 | Eceriferum-q | 410 | thioesterase and carboxyl transferase | β-diketone biosynthesis | (Schneider et al., 2016; Hen-Avivi et al., 2016)^92,93^ |
| *Hordeum vulgare* | AK373499 | Eceriferum-u | 516 | cytochrome P450 monooxygenase | β-diketone biosynthesis | (Schneider et al., 2016)^92^ |
| *A. thaliana* | AT1G78960 | LUP1 | 757 | oxidosqualene cyclase | cyclic compound biosynthesis (mixed triterpene alcohol or lupanediol) | (Segura et al., 2000; Morlacchi, 2009)^94,95^ |
| *A. thaliana* | AT1G78970 | LUP2 | 763 | oxidosqualene cyclase | cyclic compound biosynthesis (mixed triterpene alcohols) | (Kushiro et al., 2000)^96^ |
| *A. thaliana* | AT1G78950 | LUP4 | 759 | oxidosqualene cyclase | cyclic compound biosynthesis (β-amyrin) | (Shibuya et al., 2009)^97^ |
| *A. thaliana* | AT1G78500 | PEN6 | 767 | oxidosqualene cyclase | cyclic compound biosynthesis (mixed triterpene alcohols) | (Castillo-Rivera, 2014)^98^ |
| *A. thaliana* | AT2G07050 | CAS1 | 759 | oxidosqualene cyclase | cyclic compound biosynthesis (sterol) | (Babiychuk et al., 2008)^99^ |
| *Solanum lycopersicum* | NM_001247675 | TTS1 | 761 | oxidosqualene cyclase | cyclic compound biosynthesis (mixed amyrins) | (Wang et al., 2011)^100^ |
| *Solanum lycopersicum* | NM_001247668 | TTS2 | 763 | oxidosqualene cyclase | cyclic compound biosynthesis (mixed amyrins) | (Wang et al., 2011)^100^ |
| *Malus domestica* | FJ032006 | OSC1 | 760 | oxidosqualene cyclase | cyclic compound biosynthesis (α-amyrin) | (Brendolise et al., 2011)^101^ |
| *Malus domestica* | FJ032007 | OSC2 | 762 | oxidosqualene cyclase | cyclic compound biosynthesis (β-amyrin) | (Brendolise et al., 2011)^101^ |
| *Malus domestica* | FJ032008 | OSC3 | 760 | oxidosqualene cyclase | cyclic compound biosynthesis (α-amyrin) | (Brendolise et al., 2011)^101^ |
| *Malus domestica* | KT383435 | OSC4 | 761 | oxidosqualene cyclase | cyclic compound biosynthesis (germanicol) | (Andre et al., 2016)^102^ |
| *Malus domestica* | KT383436 | OSC5 | 760 | oxidosqualene cyclase | cyclic compound biosynthesis (lupeol) | (Andre et al., 2016)^102^ |
| *Pisum sativum* | KIW84_076878 | OSCPSY | 758 | oxidosqualene cyclase | cyclic compound biosynthesis (β-amyrin) | (Morita et al., 2000)^103^ |
| *Pisum sativum* | KIW84_045880 | OSCPSM | 764 | oxidosqualene cyclase | cyclic compound biosynthesis (mixed amyrins) | (Morita et al., 2000)^103^ |
| *Lagerstroemia speciosa* | AZS32327 | OSC1 | 757 | oxidosqualene cyclase | cyclic compound biosynthesis (lupeol) | (Sandeep et al., 2019)^104^ |
| *Lagerstroemia speciosa* | AZS32328 | OSC2 | 758 | oxidosqualene cyclase | cyclic compound biosynthesis (α-amyrin) | (Sandeep et al., 2019)^104^ |
| *Lagerstroemia speciosa* | AZS32329 | OSC3 | 762 | oxidosqualene cyclase | cyclic compound biosynthesis (β-amyrin) | (Sandeep et al., 2019)^104^ |
| *Lagerstroemia speciosa* | AZS32330 | OSC4 | 762 | oxidosqualene cyclase | cyclic compound biosynthesis (β-amyrin) | (Sandeep et al., 2019)^104^ |
| *Lagerstroemia speciosa* | AZS32331 | OSC5 | 757 | oxidosqualene cyclase | cyclic compound biosynthesis (cycloartenol) | (Sandeep et al., 2019)^104^ |
| *Kalopanax septemlobus* | KT150523 | BAS1 | 763 | oxidosqualene cyclase | cyclic compound biosynthesis (β-amyrin) | (Han et al., 2018)^105^ |
| *A. thaliana* | AT5G36110 | CYP716A1 | 477 | cytochrome P450 monooxygenase (C28 oxidation) | triterpenoid | (Yasumoto et al., 2016)^106^ |
| *A. thaliana* | AT5G36140 | CYP716A2 | 473 | cytochrome P450 monooxygenase (C22 oxidation) | triterpenoid | (Yasumoto et al., 2016)^106^ |
| *Medicago truncatula* | MTR_8G100135 | CYP716A12 | 479 | cytochrome P450 monooxygenase (C28 oxidation) | triterpenoid | (Fukushima et al., 2011)^107^ |
| *Vitis vinifera* | VIT_11s0065g00130 | CYP716A15 | 480 | cytochrome P450 monooxygenase (C28 oxidation) | triterpenoid | (Fukushima et al., 2011)^107^ |
| *Vitis vinifera* | VITISV_041935 | CYP716A17 | 480 | cytochrome P450 monooxygenase (C28 oxidation) | triterpenoid | (Fukushima et al., 2011)^107^ |
| *Vitis vinifera* | VIT_11s0065g00040 | CYP716A17 | 480 | cytochrome P450 monooxygenase (C28 oxidation) | triterpenoid | (Dimopoulos et al., 2020)^108^ |
| *Ocimum basilicum* | JQ958967 | CYP716A252 | 478 | cytochrome P450 monooxygenase (C28 oxidation) | triterpenoid | (Misra et al., 2017)^109^ |
| *Ocimum basilicum* | JQ958968 | CYP716A253 | 477 | cytochrome P450 monooxygenase (C28 oxidation) | triterpenoid | (Misra et al., 2017)^109^ |
| *Solanum lycopersicum* | SOLYC05G021390 | CYP716A44 | 476 | cytochrome P450 monooxygenase (C28 oxidation) | triterpenoid | (Yasumoto et al., 2017)^110^ |
| *Solanum lycopersicum* | SOLYC07G042880 | CYP716A46 | 482 | cytochrome P450 monooxygenase (C28 oxidation) | triterpenoid | (Yasumoto et al., 2017)^110^ |
| *Panax ginseng* | JX036032 | CYP716A52 | 481 | cytochrome P450 monooxygenase (C28 oxidation) | triterpenoid | (Han et al., 2013)^111^ |
| *Kalopanax septemlobus* | KT150521 | CYP716A94 | 481 | cytochrome P450 monooxygenase (C28 oxidation) | triterpenoid | (Han et al., 2018)^105^ |
| *Barbarea vulgaris* | KP795926 | CYP716A80 | 475 | cytochrome P450 monooxygenase (C28 oxidation) | triterpenoid | (Khakimov et al., 2015)^112^ |
| *Barbarea vulgaris* | KP795925 | CYP716A81 | 475 | cytochrome P450 monooxygenase (C28 oxidation) | triterpenoid | (Khakimov et al., 2015)^112^ |
| *Maesa lanceolata* | KF318733 | CYP716A75 | 487 | cytochrome P450 monooxygenase (C28 oxidation) | triterpenoid | (Moses et al., 2015)^113^ |
| *Coffea arabica* | AB706296 | CYP716A50 | 479 | cytochrome P450 monooxygenase (C28 oxidation) | triterpenoid | (Suzuki et al., 2018)^114^ |
| *Aquilegia coerulea* | KY047600 | CYP716A111 | 495 | cytochrome P450 monooxygenase (C28 oxidation) | triterpenoid | (Miettinen et al., 2017)^115^ |
| *Platycodon grandiflorus* | KU878855 | CYP716A141 | 481 | cytochrome P450 monooxygenase (C16 oxidation) | triterpenoid | (Miettinen et al., 2017)^115^ |
| *Catharanthus roseus* | JN565975 | CYP716AL1 | 480 | cytochrome P450 monooxygenase (C28 oxidation) | triterpenoid | (Huang et al., 2012)^116^ |
| *Lagerstroemia speciosa* | MG708187 | CYP716A265 (CYP1) | 480 | cytochrome P450 monooxygenase (C28 oxidation) | triterpenoid | (Sandeep et al., 2019)^104^ |
| *Lagerstroemia speciosa* | MG708188 | CYP716A266 (CYP2) | 497 | cytochrome P450 monooxygenase (C28 oxidation) | triterpenoid | (Sandeep et al., 2019)^104^ |
| *A. thaliana* | AT3G51970 | ASAT1 | 345 | acyl-CoA-sterol O-acyltransferase | cycloartenol esterification | (Bouvier-Navé et al., 2010)^117^ |
| *Malus domestica* | MG099795 | MYB52 | 217 | MYB transcription factor | regulator for cyclic compound biosynthesis | (Falginella et al., 2021)^118^ |
| *Betula platyphylla Suk.* | MF574045 | MYB21 | 337 | MYB transcription factor | regulator for cyclic compound biosynthesis | (Yin et al., 2020)^119^ |
| *Betula platyphylla Suk.* | KT344120 | MYB61 | 400 | MYB transcription factor | regulator for cyclic compound biosynthesis | (Yin et al., 2020)^119^ |
| *Betula platyphylla Suk.* | KX494862 | MYC4 | 483 | bHLH transcription factor | regulator for cyclic compound biosynthesis | (Yin et al., 2017)^120^ |
| *Betula platyphylla Suk.* | KX518840 | bHLH9 | 379 | bHLH transcription factor | regulator for cyclic compound biosynthesis | (Yin et al., 2017)^120^ |

**Table S12** Packages and software used for data processing in this study.

| **M&M section** | **Analysis performed** | **Packages or software used** | **References** |
| --- | --- | --- | --- |
| section 4.5 | quality control | Fastqc v0.11.9 | (Andrews, 2010)^121^ |
| section 4.5 | quality control | MultiQC v1.14 | (Ewels et al., 2016)^122^ |
| section 4.5 | quality control | FastP v0.20.1 | (Chen et al., 2018)^123^ |
| section 4.5 | quality control | BioBloomTools v2.3.3 | (Chu et al., 2014)^124^ |
| section 4.5 | read alignment | STAR v2.7.10a | (Dobin et al., 2013)^125^ |
| section 4.5 | differential expression analysis | DESeq2 v1.36.0 (R) | (Love et al., 2014)^126^ |
| section 4.5 | differential expression analysis | ggVennDiagram v1.2.2 (R) | (Gao et al., 2021)^127^ |
| section 4.5 | gene ontology enrichment analysis | dcGO | (Fang and Gough, 2013)^128^ |
| section 4.5 | gene co-expression network analysis | WGCNA v1.71 (R) | (Langfelder and Horvath, 2008)^129^ |
| section 4.6 | protein domain identification | InterProScan v5.53-87.0 | (Jones et al., 2014)^130^ |
| section 4.6 | reciprocal best hit blast analysis | MMseqs2 v12.113e3 | (Steinegger and Söding, 2017)^131^ |
| section 4.6 | phylogenetic analysis (multi alignment) | Clustal Omega | (Sievers and Higgins, 2014)^132^ |
| section 4.6 | phylogenetic analysis (tree construction) | IQTREE | (Nguyen et al., 2015)^133^ |
| section 4.6 | phylogenetic analysis (tree visualization) | FigTree v1.4.4 | (Rambaut, 2009)^134^ |
| section 4.7 | principal component analysis | factoextra v1.0.7 and gridExtra v2.0.0 (R) | (Kassambara and Mundt, 2020; Augie, 2015)^135,136^ |
| section 4.7 | Pearson correlation analsyis | Hmisc v4.7.1 (R) | (Harrell Jr and Dupont, 2022)^137^ |
| section 4.7 | Spearman correlation analysis | Hmisc v4.7.1 (R) | (Harrell Jr and Dupont, 2022)^137^ |

**References**

1 Ewald R, Kolukisaoglu Ü, Bauwe U, Mikkat S, Bauwe H. Mitochondrial protein lipoylation does not exclusively depend on the mtKAS pathway of de novo fatty acid synthesis in arabidopsis. *Plant Physiol* 2007; **145**: 41–48.

2 Carlsson AS, LaBrie ST, Kinney AJ, Von Wettstein-Knowles P, Browse J. A KAS2 cDNA complements the phenotypes of the Arabidopsis fab1 mutant that differs in a single residue bordering the substrate binding pocket. *Plant J* 2002; **29**: 761–770.

3 Tai H, Post-Beittenmiller D, Jaworski JG. Cloning of a cDNA encoding 3-ketoacyl-acyl carrier protein synthase III from Arabidopsis. *Plant Physiol* 1994; **106**: 801–802.

4 Shimakata T, Stumpf PK. Isolation and function of spinach leaf β-ketoacyl-[acyl-carrier-protein] synthases. *Proc Natl Acad Sci* 1982; **79**: 5808–5812.

5 Clough RC, Matthis AL, Barnums SR *et al.* Purification and characterization of 3-ketoacyl-acyl carrier protein synthase III from spinach. A condensing enzyme utilizing acetyl-coenzyme A to initiate fatty acid synthesis. *J Biol Chem* 1992; **267**: 20992–20998.

6 Tai H, Jaworski JG. 3-Ketoacyl-acyl carrier protein synthase III from spinach (Spinacia oleracea) is not similar to other condensing enzymes of fatty acid synthase. *Plant Physiol* 1993; **103**: 1361–1367.

7 Shimakata K, Stumpf PK. Purification and characterizations of β-ketoacyl-[acyl-carrier-protein] reductase, β-hydroxyacyl-[acyl-carrier-protein] dehydrase, and enoyl-[acyl-carrier-protein] reductase from Spinacia oleracea leaves. *Arch Biochem Biophys* 1982; **218**: 77–91.

8 Brown A, Affleck V, Kroon J, Slabas A. Proof of function of a putative 3-hydroxyacyl-acyl carrier protein dehydratase from higher plants by mass spectrometry of product formation. *FEBS Lett* 2009; **583**: 363–368.

9 Chen C, Li C, Wang Y *et al.* Cytosolic acetyl-CoA promotes histone acetylation predominantly at H3K27 in Arabidopsis. *Nat Plants* 2017; **3**: 814–824.

10 Bonaventure G, Salas JJ, Pollard MR, Ohlrogge JB. Disruption of the FATB gene in Arabidopsis demonstrates an essential role of saturated fatty acids in plant growth. *Plant Cell* 2003; **15**: 1020–1033.

11 Lü S, Song T, Kosma DK, Parsons EP, Rowland O, Jenks MA. Arabidopsis CER8 encodes LONG-CHAIN ACYL-COA SYNTHETASE 1 (LACS1) that has overlapping functions with LACS2 in plant wax and cutin synthesis. *Plant J* 2009; **59**: 553–564.

12 Zhao L, Katavic V, Li F, Haughn GW, Kunst L. Insertional mutant analysis reveals that long-chain acyl-CoA synthetase 1 (LACS1), but not LACS8, functionally overlaps with LACS9 in Arabidopsis seed oil biosynthesis. *Plant J* 2010; **64**: 1048–1058.

13 Schnurr J, Shockey J, Browse J. The Acyl-CoA synthetase encoded by LACS2 is essential for normal cuticle development in arabidopsis. *Plant Cell* 2004; **16**: 629–642.

14 Shockey JM, Fulda MS, Browse JA. Arabidopsis contains nine long-chain acyl-coenzyme A synthetase genes that participate in fatty acid and glycerolipid metabolism. *Plant Physiol* 2002; **129**: 1710–1722.

15 Fulda M, Schnurr J, Abbadi A, Heinz E, Browse J. Peroxisomal acyl-CoA synthetase activity is essential for seedling development in Arabidopsis thaliana. *Plant Cell* 2004; **16**: 394–405.

16 Fulda M, Shockey J, Werber M, Wolter FP, Heinz E. Two long-chain acyl-CoA synthetases from Arabidopsis thaliana involved in peroxisomal fatty acid β-oxidation. *Plant J* 2002; **32**: 93–103.

17 Lü S, Zhao H, Des Marais DL *et al.* Arabidopsis ECERIFERUM9 involvement in cuticle formation and maintenance of plant water status. *Plant Physiol* 2012; **159**: 930–944.

18 Todd J, Post-Beittenmiller D, Jaworski JG. KCS1 encodes a fatty acid elongase 3-ketoacyl-CoA synthase affecting wax biosynthesis in Arabidopsis thaliana. *Plant J* 1999; **17**: 119–130.

19 Lee SB, Jung SJ, Go YS *et al.* Two Arabidopsis 3-ketoacyl CoA synthase genes, KCS20 and KCS2/DAISY, are functionally redundant in cuticular wax and root suberin biosynthesis, but differentially controlled by osmotic stress. *Plant J* 2009; **60**: 462–475.

20 Huang H, Yang X, Zheng M *et al.* An ancestral role for 3-KETOACYL-COA SYNTHASE3 as a negative regulator of plant cuticular wax synthesis. *Plant Cell* 2023; : 1–20.

21 Kim J, Lee SB, Suh MC. Arabidopsis 3-ketoacyl-CoA synthase 4 is essential for root and pollen tube growth. *J Plant Biol* 2021; **64**: 155–165.

22 Fiebig A, Mayfield JA, Miley NL, Chau S, Fischer RL, Preuss D. Alterations in CER6, a gene identical to CUT1, differentially affect long-chain lipid content on the surface of pollen and stems. *Plant Cell* 2000; **12**: 2001–2008.

23 Millar AA, Clemens S, Zachgo S, Giblin EM, Taylor DC, Kunst L. CUT1, an Arabidopsis gene required for cuticular wax biosynthesis and pollen fertility, encodes a very-long-chain fatty acid condensing enzyme. *Plant Cell* 1999; **11**: 825–838.

24 Kim J, Jung JH, Lee SB *et al.* Arabidopsis 3-ketoacyl-coenzyme a synthase9 is involved in the synthesis of tetracosanoic acids as precursors of cuticular waxes, suberins, sphingolipids, and phospholipids. *Plant Physiol* 2013; **162**: 567–580.

25 Chai M, Castillo IQ, Sonntag A *et al.* A seed coat-specific b-ketoacyl-CoA synthase, KCS12, is critical for preserving seed physical dormancy. *Plant Physiol* 2021; **186**: 1606–1615.

26 Hegebarth D, Buschhaus C, Joubès J, Thoraval D, Bird D, Jetter R. Arabidopsis ketoacyl-CoA synthase 16 (KCS16) forms C36/C38 acyl precursors for leaf trichome and pavement surface wax. *Plant Cell Environ* 2017; **40**: 1761–1776.

27 Jasinski S, Lécureuil A, Miquel M *et al.* Natural variation in seed very long chain fatty acid content is controlled by a new isoform of KCS18 in Arabidopsis thaliana. *PLoS One* 2012; **7**: e49261.

28 Haslam TM, Mañas-Fernández A, Zhao L, Kunst L. Arabidopsis ECERIFERUM2 Is a component of the fatty acid elongation machinery required for fatty acid extension to exceptional lengths. *Plant Physiol* 2012; **160**: 1164–1174.

29 Pascal S, Bernard A, Sorel M *et al.* The Arabidopsis cer26 mutant, like the cer2 mutant, is specifically affected in the very long chain fatty acid elongation process. *Plant J* 2013; **73**: 733–746.

30 Alexander LE, Okazaki Y, Schelling MA *et al.* Maize glossy2 and glossy2-like genes have overlapping and distinct functions in cuticular lipid deposition. *Plant Physiol* 2020; **183**: 840–853.

31 Leide J, Hildebrandt U, Reussing K, Riederer M, Vogg G. The developmental pattern of tomato fruit wax accumulation and its impact on cuticular transpiration barrier properties: Effects of a deficiency in a β-ketoacyl-coenzyme A synthase (LeCER6). *Plant Physiol* 2007; **144**: 1667–1679.

32 Qin YM, Hu CY, Pang Y, Kastaniotis AJ, Hiltunen JK, Zhu YX. Saturated very-long-chain fatty acids promote cotton fiber and Arabidopsis cell elongation by activating ethylene biosynthesis. *Plant Cell* 2007; **19**: 3692–3704.

33 Beaudoin F, Wu X, Li F *et al.* Functional characterization of the Arabidopsis β-ketoacyl-coenzyme a reductase candidates of the fatty acid elongase. *Plant Physiol* 2009; **150**: 1174–1191.

34 Dietrich CR, Perera MADN, Yandeau-Nelson MD, Meeley RB, Nikolau BJ, Schnable PS. Characterization of two GL8 paralogs reveals that the 3-ketoacyl reductase component of fatty acid elongase is essential for maize (Zea mays L.) development. *Plant J* 2005; **42**: 844–861.

35 Carol RJ, Breiman A, Erel N, Vittorioso P, Bellini C. PASTICCINO1 (AtFKBP70) is a nuclear-localised immunophilin required during Arabidopsis thaliana embryogenesis. *Plant Sci* 2001; **161**: 527–535.

36 Bach L, Michaelson L V., Haslam R *et al.* The very-long-chain hydroxy fatty acyl-CoA dehydratase PASTICCINO2 is essential and limiting for plant development. *Proc Natl Acad Sci U S A* 2008; **105**: 14727–14731.

37 Zheng H, Rowland O, Kunst L. Disruptions of the Arabidopsis enoyl-CoA reductase gene reveal an essential role for very-long-chain fatty acid synthesis in cell expansion during plant morphogenesis. *Plant Cell* 2005; **17**: 1467–1481.

38 Kosma DK, Molina I, Ohlrogge JB, Pollard M. Identification of an Arabidopsis fatty alcohol: Caffeoyl-Coenzyme a acyltransferase required for the synthesis of alkyl hydroxycinnamates in root waxes. *Plant Physiol* 2012; **160**: 237–248.

39 Rowland O, Zheng H, Hepworth SR, Lam P, Jetter R, Kunst L. CER4 encodes an alcohol-forming fatty acyl-coenzyme A reductase involved in cuticular wax production in Arabidopsis. *Plant Physiol* 2006; **142**: 866–877.

40 Gupta NC, Jain PK, Bhat SR, Srinivasan R. Upstream sequence of fatty acyl-CoA reductase (FAR6) of Arabidopsis thaliana drives wound-inducible and stem-specific expression. *Plant Cell Rep* 2012; **31**: 839–850.

41 Wang Y, Wang M, Sun Y *et al.* Molecular characterization of TaFAR1 involved in primary alcohol biosynthesis of cuticular wax in hexaploid wheat. *Plant Cell Physiol* 2015; **56**: 1944–1961.

42 Li F, Wu X, Lam P *et al.* Identification of the wax ester synthase/acyl-coenzyme a:diacylglycerol acyltransferase WSD1 required for stem wax ester biosynthesis in Arabidopsis. *Plant Physiol* 2008; **148**: 97–107.

43 Patwari P, Salewski V, Gutbrod K *et al.* Surface wax esters contribute to drought tolerance in Arabidopsis. *Plant J* 2019; **98**: 727–744.

44 Salewski V. *Wax Ester Synthases/Acyl-CoA:Diacylglycerol Acyltransferases (WS/DGATs) from Arabidopsis thaliana*. 2022.https://bonndoc.ulb.uni-bonn.de/xmlui/handle/20.500.11811/9897.

45 King A, Nam JW, Han J, Hilliard J, Jaworski JG. Cuticular wax biosynthesis in petunia petals: Cloning and characterization of an alcohol-acyltransferase that synthesizes wax-esters. *Planta* 2007; **226**: 381–394.

46 Lardizabal KD, Metz JG, Sakamoto T, Hutton WC, Pollard MR, Lassner MW. Purification of a jojoba embryo wax synthase, cloning of its cDNA, and production of high levels of wax in seeds of transgenic Arabidopsis. *Plant Physiol* 2000; **122**: 645–655.

47 Yang X, Zhao H, Kosma DK *et al.* The Acyl desaturase CER17 is involved in producing Wax unsaturated primary Alcohols and cutin monomers. *Plant Physiol* 2017; **173**: 1109–1124.

48 Heilmann I, Mekhedov S, King B, Browse J, Shanklin J. Identification of the Arabidopsis palmitoyl-monogalactosyldiacylglycerol delta7-desaturase gene FAD5, and effects of plastidial retargeting of Arabidopsis desaturases on the fad5 mutant phenotype. *Plant Physiol* 2004; **136**: 4237–4245.

49 Chen X, Goodwin SM, Boroff VL, Liu X, Jenks MA. Cloning and characterization of the WAX2 gene of Arabidopsis involved in cuticle membrane and wax production. *Plant Cell* 2003; **15**: 1170–1185.

50 Bernard A, Domergue F, Pascal S *et al.* Reconstitution of plant alkane biosynthesis in yeast demonstrates that Arabidopsis ECERIFERUM1 and ECERIFERUM3 are core components of a very-long-chain alkane synthesis complex. *Plant Cell* 2012; **24**: 3106–3118.

51 Rowland O, Lee R, Franke R, Schreiber L, Kunst L. The CER3 wax biosynthetic gene from Arabidopsis thaliana is allelic to WAX2/YRE/FLP1. *FEBS Lett* 2007; **581**: 3538–3544.

52 Hannoufa A, Negruk V, Eisner G, Lemieux B. The CER3 gene of Arabidopsis thaliana is expressed in leaves, stems, roots, flowers, and aplical meristems. *Plant J* 1996; **10**: 459–467.

53 Sturaro M, Hartings H, Schmelzer E, Velasco R, Salamini F, Motto M. Cloning and characterization of GLOSSY1, a maize gene involved in cuticle membrane and wax production. *Plant Physiol* 2005; **138**: 478–489.

54 Hooker TS, Lam P, Zheng H, Kunst L. A core subunit of the RNA-processing/degrading exosome specifically influences cuticular wax biosynthesis in Arabidopsis. *Plant Cell* 2007; **19**: 904–913.

55 Lam P, Zhao L, McFarlane HE *et al.* RDR1 and SGS3, components of RNA-mediated gene silencing, are required for the regulation of cuticular wax biosynthesis in developing inflorescence stems of arabidopsis. *Plant Physiol* 2012; **159**: 1385–1395.

56 Wang T, Xing J, Liu X *et al.* GCN5 contributes to stem cuticular wax biosynthesis by histone acetylation of CER3 in Arabidopsis. *J Exp Bot* 2018; **69**: 2911–2922.

57 Kim H, Yu S in, Jung SH, Lee B ha, Suh MC. The F-box protein SAGL1 and ECeRIFERUM3 regulate cuticular wax biosynthesis in response to changes in humidity in Arabidopsis. *Plant Cell* 2019; **31**: 2223–2240.

58 Sakuradani E, Zhao L, Haslam TM, Kunst L. The CER22 gene required for the synthesis of cuticular wax alkanes in Arabidopsis thaliana is allelic to CER1. *Planta* 2013; **237**: 731–738.

59 Pascal S, Bernard A, Deslous P *et al.* Arabidopsis CER1-LIKE1 functions in a cuticular very-long-chain alkane-forming complex. *Plant Physiol* 2019; **179**: 415–432.

60 Wu H, Liu L, Chen Y *et al.* Tomato SlCER1-1 catalyzes the synthesis of wax alkanes, increasing drought tolerance and fruit storability. *Hortic Res* 2022; **9**: 1–14.

61 Greer S, Wen M, Bird D *et al.* The cytochrome P450 enzyme CYP96A15 is the midchain alkane hydroxylase responsible for formation of secondary alcohols and ketones in stem cuticular wax of arabidopsis. *Plant Physiol* 2007; **145**: 653–667.

62 Lee SB, Go YS, Bae HJ *et al.* Disruption of glycosylphosphatidylinositol-anchored lipid transfer protein gene altered cuticular lipid composition, increased plastoglobules, and enhanced susceptibility to infection by the fungal pathogen alternaria brassicicola. *Plant Physiol* 2009; **150**: 42–54.

63 Kim H, Lee SB, Kim HJ, Min MK, Hwang I, Suh MC. Characterization of glycosylphosphatidylinositol-anchored lipid transfer protein 2 (LTPG2) and overlapping function between LTPG/LTPG1 and LTPG2 in cuticular wax export or accumulation in arabidopsis thaliana. *Plant Cell Physiol* 2012; **53**: 1391–1403.

64 Bird D, Beisson F, Brigham A *et al.* Characterization of Arabidopsis ABCG11/WBC11, an ATP binding cassette (ABC) transporter that is required for cuticular lipid secretion. *Plant J* 2007; **52**: 485–498.

65 Pighin JA, Zheng H, Balakshin LJ *et al.* Plant cuticular lipid export requires an ABC transporter. *Science (80- )* 2004; **306**: 702–704.

66 Panikashvili D, Shi JX, Schreiber L, Aharoni A. The Arabidopsis ABCG13 transporter is required for flower cuticle secretion and patterning of the petal epidermis. *New Phytol* 2011; **190**: 113–124.

67 Liu F, Xiong X, Wu L *et al.* BraLTP1, a lipid transfer protein gene involved in epicuticular wax deposition, cell proliferation and flower development in Brassica napus. *PLoS One* 2014; **9**: 1–12.

68 Kannangara R, Branigan C, Liu Y *et al.* The transcription factor WIN1/SHN1 regulates cutin biosynthesis in Arabidopsis thaliana. *Plant Cell* 2007; **19**: 1278–1294.

69 Aharoni A, Dixit S, Jetter R, Thoenes E, Van Arkel G, Pereira A. The SHINE clade of AP2 domain transcription factors activates wax biosynthesis, alters cuticle properties, and confers drought tolerance when overexpressed in Arabidopsis. *Plant Cell* 2004; **16**: 2463–2480.

70 Broun P, Poindexter P, Osborne E, Jiang C-Z, Riechmann JL. WIN1, a transcriptional activator of epidermal wax accumulation in Arabidopsis. *Proc Natl Acedemy Sci* 2004; **101**: 4706–4711.

71 Shi JX, Malitsky S, de Oliveira S *et al.* SHINE transcription factors act redundantly to pattern the archetypal surface of arabidopsis flower organs. *PLoS Genet* 2011; **7**. doi:10.1371/journal.pgen.1001388.

72 Cernac A, Benning C. WRINKLED1 encodes an AP2/EREB domain protein involved in the control of storage compound biosynthesis in Arabidopsis. *Plant J* 2004; **40**: 575–585.

73 To A, Joubès J, Barthole G *et al.* WRINKLED transcription factors orchestrate tissue-specific regulation of fatty acid biosynthesis in Arabidopsis. *Plant Cell* 2013; **24**: 5007–5023.

74 Park CS, Go YS, Suh MC. Cuticular wax biosynthesis is positively regulated by WRINKLED4, an AP2/ERF-type transcription factor, in Arabidopsis stems. *Plant J* 2016; **88**: 257–270.

75 Taketa S, Amano S, Tsujino Y *et al.* Barley grain with adhering hulls is controlled by an ERF family transcription factor gene regulating a lipid biosynthesis pathway. *Proc Natl Acad Sci* 2008; **105**: 4062–4067.

76 Bi H, Shi J, Kovalchuk N *et al.* Overexpression of the TaSHN1 transcription factor in bread wheat leads to leaf surface modifications, improved drought tolerance, and no yield penalty under controlled growth conditions. *Plant Cell Environ* 2018; **41**: 2549–2566.

77 Shi JX, Adato A, Alkan N *et al.* The tomato SlSHINE3 transcription factor regulates fruit cuticle formation and epidermal patterning. *New Phytol* 2013; **197**: 468–480.

78 Zhang JY, Broeckling CD, Blancaflor EB, Sledge MK, Sumner LW, Wang ZY. Overexpression of WXP1, a putative Medicago truncatula AP2 domain-containing transcription factor gene, increases cuticular wax accumulation and enhances drought tolerance in transgenic alfalfa (Medicago sativa). *Plant J* 2005; **42**: 689–707.

79 Go YS, Kim H, Kim HJ, Suh MC. Arabidopsis cuticular wax biosynthesis is negatively regulated by the DEWAX gene encoding an AP2/ERF-type transcription factor. *Plant Cell* 2014; **26**: 1666–1680.

80 Kim H, Go YS, Suh MC. DEWAX2 transcription factor negatively regulates cuticular wax biosynthesis in Arabidopsis leaves. *Plant Cell Physiol* 2018; **59**: 966–977.

81 Raffaele S, Vailleau F, Léger A *et al.* A MYB transcription factor regulates very-long-chain fatty acid biosynthesis for activation of the hypersensitive cell death response in Arabidopsis. *Plant Cell* 2008; **20**: 752–767.

82 Marino D, Froidure S, Canonne J *et al.* Arabidopsis ubiquitin ligase MIEL1 mediates degradation of the transcription factor MYB30 weakening plant defence. *Nat Commun* 2013; **4**: 1476.

83 Cominelli E, Sala T, Calvi D, Gusmaroli G, Tonelli C. Over-expression of the Arabidopsis AtMYB41 gene alters cell expansion and leaf surface permeability. *Plant J* 2008; **53**: 53–64.

84 Lee SB, Suh MC. Cuticular wax biosynthesis is up-regulated by the MYB94 transcription factor in arabidopsis. *Plant Cell Physiol* 2015; **56**: 48–60.

85 Lee SB, Kim HU, Suh MC. MYB94 and MYB96 additively activate cuticular wax biosynthesis in Arabidopsis. *Plant Cell Physiol* 2016; **57**: 2300–2311.

86 Seo PJ, Xiang F, Qiao M *et al.* The MYB96 transcription factor mediates abscisic acid signaling during drought stress response in Arabidopsis. *Plant Physiol* 2009; **151**: 275–289.

87 Seo PJ, Lee SB, Suh MC, Park MJ, Park CM. The MYB96 transcription factor regulates cuticular wax biosynthesis under drought conditions in arabidopsis. *Plant Cell* 2011; **23**: 1138–1152.

88 Oshima Y, Shikata M, Koyama T, Ohtsubo N, Mitsuda N, Ohme-Takagi M. MIXTA-like transcription factors and WAX INDUCER1/SHINE1 coordinately regulate cuticle development in Arabidopsis and Torenia fournieri. *Plant Cell* 2013; **25**: 1609–1624.

89 Wang Z, Tian X, Zhao Q *et al.* The E3 ligase drought hypersensitive negatively regulates cuticular wax biosynthesis by promoting the degradation of transcription factor ROC4 in rice. *Plant Cell* 2018; **30**: 228–244.

90 Wu R, Li S, He S *et al.* CFL1, a WW domain protein, regulates cuticle development by modulating the function of HDG1, a class IV homeodomain transcription factor, in rice and arabidopsis. *Plant Cell* 2011; **23**: 3392–3411.

91 Javelle M, Vernoud V, Depège-Fargeix N *et al.* Overexpression of the epidermis-specific homeodomain-leucine zipper IV transcription factor OUTER CELL LAYER1 in maize identifies target genes involved in lipid metabolism and cuticle biosynthesis. *Plant Physiol* 2010; **154**: 273–286.

92 Schneider LM, Adamski NM, Christensen CE *et al.* The Cer-cqu gene cluster determines three key players in a β-diketone synthase polyketide pathway synthesizing aliphatics in epicuticular waxes. *J Exp Bot* 2016; **67**: 2715–2730.

93 Hen-Avivi S, Savin O, Racovita RC *et al.* A metabolic gene cluster in the wheat W1 and the barley Cer-cqu loci determines β-diketone biosynthesis and glaucousness. *Plant Cell* 2016; **28**: 1440–1460.

94 Segura MJR, Meyer MM, Matsuda SPT. Arabidopsis thaliana LUP1 converts oxidosqualene to multiple triterpene alcohols and a triterpene diol. *Org Lett* 2000; **2**: 2257–2259.

95 Morlacchi P. *Triterpenoid biosynthesis in plants: Oxidosqualene cyclization in Arabidopsis thaliana*. 2009. doi:10.1016/j.jaci.2012.05.050.

96 Kushiro T, Shibuya M, Masuda K, Ebizuka Y. Mutational studies on triterpene synthases: Engineering lupeol synthase into β-amyrin synthase. *J Am Chem Soc* 2000; **122**: 6816–6824.

97 Shibuya M, Katsube Y, Otsuka M *et al.* Identification of a product specific β-amyrin synthase from Arabidopsis thaliana. *Plant Physiol Biochem* 2009; **47**: 26–30.

98 Castillo-Rivera DA. *A Journey through the Arabidopsis thaliana genome: Discovering the origins of novel triterpene metabolites*. 2014.

99 Babiychuk E, Bouvier-Nave P, Compagnon V *et al.* Allelic mutant series reveal distinct functions for Arabidopsis cycloartenol synthase 1 in cell viability and plastid biogenesis. *Proc Natl Acad Sci U S A* 2008; **105**: 3163–3168.

100 Wang Z, Guhling O, Yao R *et al.* Two oxidosqualene cyclases responsible for biosynthesis of tomato fruit cuticular triterpenoids. *Plant Physiol* 2011; **155**: 540–552.

101 Brendolise C, Yauk YK, Eberhard ED *et al.* An unusual plant triterpene synthase with predominant α-amyrin- producing activity identified by characterizing oxidosqualene cyclases from Malus × domestica. *FEBS J* 2011; **278**: 2485–2499.

102 Andre CM, Legay S, Deleruelle A *et al.* Multifunctional oxidosqualene cyclases and cytochrome P450 involved in the biosynthesis of apple fruit triterpenic acids. *New Phytol* 2016; **211**: 1279–1294.

103 Morita M, Shibuya M, Kushiro T, Masuda K, Ebizuka Y. Molecular cloning and functional expression of triterpene synthases from pea (Pisum sativum): New α-amyrin-producing enzyme is a multifunctional triterpene synthase. *Eur J Biochem* 2000; **267**: 3453–3460.

104 Sandeep, Misra RC, Chanotiya CS, Mukhopadhyay P, Ghosh S. Oxidosqualene cyclase and CYP716 enzymes contribute to triterpene structural diversity in the medicinal tree banaba. *New Phytol* 2019; **222**: 408–424.

105 Han JY, Chun JH, Oh SA *et al.* Transcriptomic analysis of Kalopanax septemlobus and characterization of KsBAS, CYP716A94 and CYP72A397 genes involved in hederagenin saponin biosynthesis. *Plant Cell Physiol* 2018; **59**: 319–330.

106 Yasumoto S, Fukushima EO, Seki H, Muranaka T. Novel triterpene oxidizing activity of Arabidopsis thaliana CYP716A subfamily enzymes. *FEBS Lett* 2016; **590**: 533–540.

107 Fukushima EO, Seki H, Ohyama K *et al.* CYP716A subfamily members are multifunctional oxidases in triterpenoid biosynthesis. *Plant Cell Physiol* 2011; **52**: 2050–2061.

108 Dimopoulos N, Tindjau R, Wong DCJ *et al.* Drought stress modulates cuticular wax composition of the grape berry. *J Exp Bot* 2020; **71**: 3126–3141.

109 Misra RC, Sharma S, Sandeep S, Garg A, Chanotiya CS, Ghosh S. Two CYP716A subfamily cytochrome P450 monooxygenases of sweet basil play similar but nonredundant roles in ursane- and oleanane-type pentacyclic triterpene biosynthesis. *New Phytol* 2017; **214**: 706–720.

110 Yasumoto S, Seki H, Shimizu Y, Fukushima EO, Muranaka T. Functional characterization of CYP716 family P450 enzymes in triterpenoid biosynthesis in tomato. *Front Plant Sci* 2017; **8**: 21.

111 Han JY, Kim MJ, Ban YW, Hwang HS, Choi YE. The involvement of β-amyrin 28-oxidase (CYP716A52v2) in oleanane-type ginsenoside biosynthesis in Panax ginseng. *Plant Cell Physiol* 2013; **54**: 2034–2046.

112 Khakimov B, Kuzina V, Erthmann P *et al.* Identification and genome organization of saponin pathway genes from a wild crucifer, and their use for transient production of saponins in Nicotiana benthamiana. *Plant J* 2015; **84**: 478–490.

113 Moses T, Pollier J, Faizal A *et al.* Unraveling the triterpenoid saponin biosynthesis of the african shrub maesa lanceolata. *Mol Plant* 2015; **8**: 122–135.

114 Suzuki H, Fukushima EO, Umemoto N, Ohyama K, Seki H, Muranaka T. Comparative analysis of CYP716A subfamily enzymes for the heterologous production of C-28 oxidized triterpenoids in transgenic yeast. *Plant Biotechnol* 2018; **35**: 131–139.

115 Miettinen K, Pollier J, Buyst D *et al.* The ancient CYP716 family is a major contributor to the diversification of eudicot triterpenoid biosynthesis. *Nat Commun* 2017; **8**: 14153.

116 Huang L, Li J, Ye H *et al.* Molecular characterization of the pentacyclic triterpenoid biosynthetic pathway in Catharanthus roseus. *Planta* 2012; **236**: 1571–1581.

117 Bouvier-Navé P, Berna A, Noiriel A *et al.* Involvement of the Phospholipid sterol Acyltransferase1 in plant sterol homeostasis and leaf senescence. *Plant Physiol* 2010; **152**: 107–119.

118 Falginella L, Andre CM, Legay S *et al.* Differential regulation of triterpene biosynthesis induced by an early failure in cuticle formation in apple. *Hortic Res* 2021; **8**. doi:10.1038/s41438-021-00511-4.

119 Yin J, Sun L, Li Y *et al.* Functional identification of BpMYB21 and BpMYB61 transcription factors responding to MeJA and SA in birch triterpenoid synthesis. *BMC Plant Biol* 2020; **20**: 1–22.

120 Yin J, Li X, Zhan Y *et al.* Cloning and expression of BpMYC4 and BpbHLH9 genes and the role of BpbHLH9 in triterpenoid synthesis in birch. *BMC Plant Biol* 2017; **17**: 1–14.

121 Andrews S. FastQC: a quality control tool for high throughput sequence data. 2010. http://www.bioinformatics.babraham.ac.uk/projects/fastqc

122 Ewels P, Magnusson M, Lundin S, Käller M. MultiQC: Summarize analysis results for multiple tools and samples in a single report. *Bioinformatics* 2016; **32**: 3047–3048.

123 Chen S, Zhou Y, Chen Y, Gu J. Fastp: An ultra-fast all-in-one FASTQ preprocessor. *Bioinformatics* 2018; **34**: i884–i890.

124 Chu J, Sadeghi S, Raymond A *et al.* BioBloom tools: Fast, accurate and memory-efficient host species sequence screening using bloom filters. *Bioinformatics* 2014; **30**: 3402–3404.

125 Dobin A, Davis CA, Schlesinger F *et al.* STAR: Ultrafast universal RNA-seq aligner. *Bioinformatics* 2013; **29**: 15–21.

126 Love MI, Huber W, Anders S. Moderated estimation of fold change and dispersion for RNA-seq data with DESeq2. *Genome Biol* 2014; **15**: 1–21.

127 Gao CH, Yu G, Cai P. ggVennDiagram: An Intuitive, Easy-to-Use, and Highly Customizable R Package to Generate Venn Diagram. *Front Genet* 2021; **12**: 1598.

128 Fang H, Gough J. DcGO: Database of domain-centric ontologies on functions, phenotypes, diseases and more. *Nucleic Acids Res* 2013; **41**: D536–D564.

129 Langfelder P, Horvath S. WGCNA: An R package for weighted correlation network analysis. *BMC Bioinformatics* 2008; **9**: 599.

130 Jones P, Binns D, Chang HY *et al.* InterProScan 5: Genome-scale protein function classification. *Bioinformatics* 2014; **30**: 1236–1240.

131 Steinegger M, Söding J. MMseqs2 enables sensitive protein sequence searching for the analysis of massive data sets. *Nat Biotechnol* 2017; **35**: 1026–1028.

132 Sievers F, Higgins DG. Clustal omega. *Curr Protoc Bioinforma* 2014; **48**: 3–13.

133 Nguyen LT, Schmidt HA, Von Haeseler A, Minh BQ. IQ-TREE: A fast and effective stochastic algorithm for estimating maximum-likelihood phylogenies. *Mol Biol Evol* 2015; **32**: 268–274.

134 Rambaut A. FigTree v1.3.1. Institute of Evolutionary Biology, University of Edinburgh, Edinburgh. 2010. http://tree.bio.ed.ac.uk/software/figtree/

135 Kassambara A, Mundt F. Factoextra: Extract and Visualize the Results of Multivariate Data Analyses. R Package Version 1.0.7. 2020. https://CRAN.R-project.org/package=factoextra

136 Augie B. gridExtra: Miscellaneous functions for “Grid” graphics. R Package Version 2.0.0. 2015. http://CRAN.R-project.org/package=gridExtra

137 Harrell Jr F, Dupont C. Hmisc: Harrell Mmscellaneous. R Package Version 4.7.1. 2022. https://CRAN.R-project.org/package=Hmisc.
