# Supplementary material for: Cuticular wax biosynthesis in blueberries (Vaccinium corymbosum L.): Transcript and metabolite changes during ripening and storage affect key fruit quality traits
Source: Hortic Res. 2024 Jan 9;11(3):uhae004. doi: 10.1093/hr/uhae004 (PMC10923646; doi:10.1093/hr/uhae004)
Supplement: Web_Material_uhae004 [file web_material_uhae004.zip › Supplementary meterial.docx]

**Supplemental material 1.** Workflow for the identification of wax-related genes in blueberries


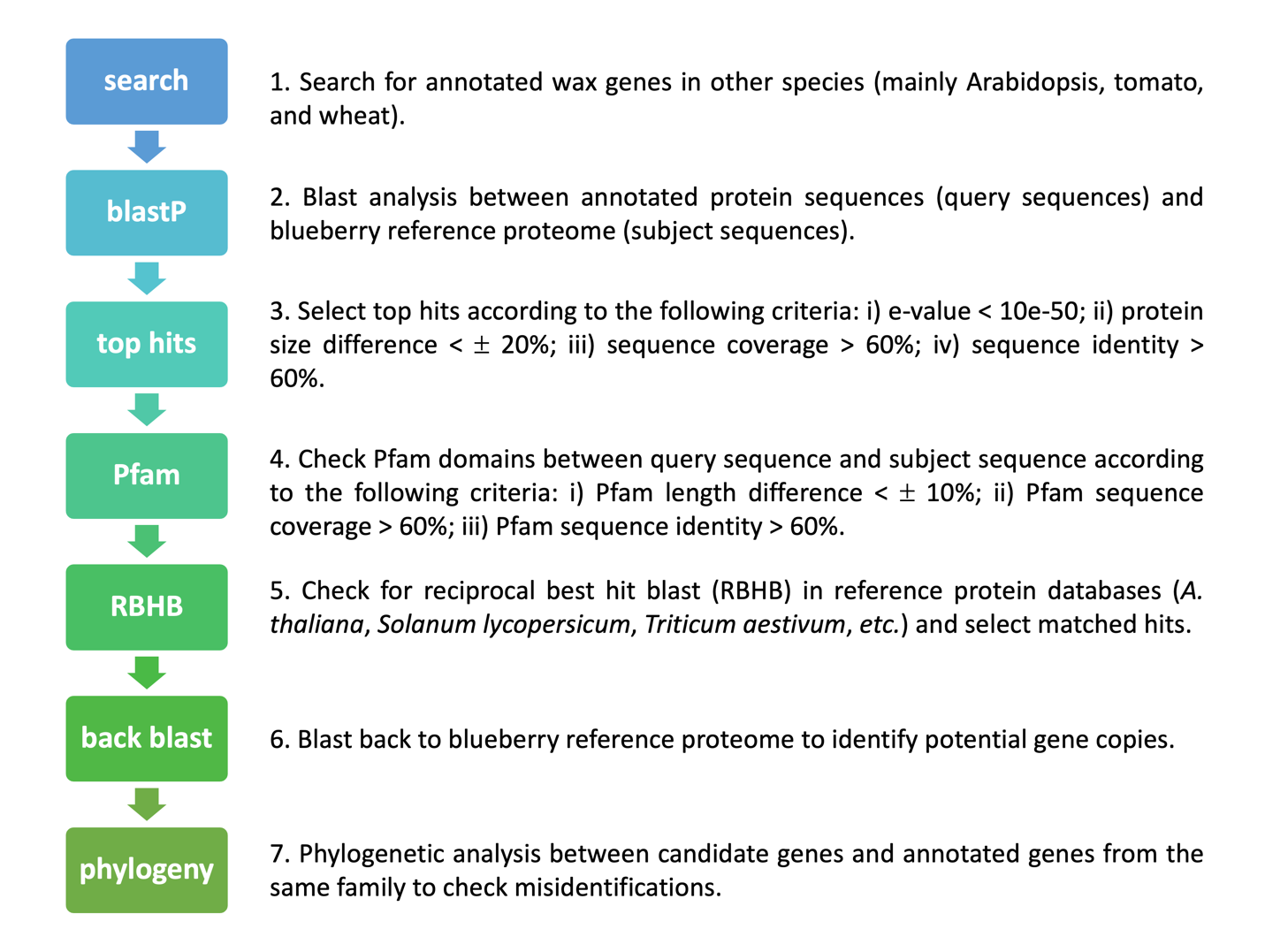


**Supplemental material 2A.** Scripts used for quality check of the transcriptome data using Fastqc v0.11.9

| fastp -i $fastq1 -I $fastq2 \  -o ${nam1}fastp.fq.gz -O ${nam2}fastp.fq.gz \  -w 24 2> fastp.log |
| --- |

**Supplemental material 2B.** Scripts used for checking the contamination from any microorganisms using BioBloomTools v2.3.3

| biobloomcategorizer -p cont_BBT -t 24 \  -e \  -i \  -f "aphids.bf mites.bf fungi.bf archaea.bf bacteria.bf protozoa.bf thrips.bf univec.bf viral.bf" \  $fastq1 $fastq2 |
| --- |

**Supplemental material 2C.** Scripts used for read mapping and quantification using STAR v2.7.10a

| STAR --runThreadN 24 \  --runMode alignReads \  --readFilesCommand zcat \  --sjdbScore 2 \  --sjdbOverhang 149 \  --limitSjdbInsertNsj 1000000 \  --outFilterMultimapNmax 10 \  --alignSJoverhangMin 8 \  --alignSJDBoverhangMin 1 \  --outFilterMismatchNmax 999 \  --outFilterMismatchNoverReadLmax 0.04 \  --alignIntronMin 20 \  --alignIntronMax 1000000 \  --alignMatesGapMax 1000000 \  --outSAMunmapped Within \  --outFilterType BySJout \  --outSAMattributes NH HI AS NM MD \  --outSAMtype BAM SortedByCoordinate \  --quantMode GeneCounts \  --twopassMode Basic \  --outTmpDir _STARtmp \  --outFileNamePrefix $my_dir \  --genomeDir starIndex \  --sjdbGTFfile V_corymbosum_Draper_v1.0-genesTruncated.gtf \  --readFilesIn $fastq1 $fastq2 |
| --- |
